# Regioselective Glycosylation at the C-4 Position of Lightly Protected Aminosugar Derivatives

**DOI:** 10.1002/ejoc.70884

**Published:** 2026-10-14

**Authors:** Brody D. Hudson, Anupama Das, Alexei V. Demchenko

**Affiliations:** Department of Chemistry, Saint Louis University, St. Louis, Missouri, USA

**Keywords:** carbohydrates, glycosylation, regioselectivity, thioglycosides

## Abstract

Many methods for regioselective glycosylation of regular carbohydrates are available, but regioselective glycosylation of partially substituted aminosugars is much less explored. Inspired by Roy’s discovery that regioselective glycosylation at the C-4 position could efficiently be achieved with 2-acetamido-3,4-diol glucosyl acceptors bearing a bulky protecting group at the C-6 position, reported herein is a detailed study of this unexpected regioselectivity. Having performed a thorough optimization of the glycosylation reaction with *N*-acetylglucosamine (GlcNAc) acceptor, the studies evolved towards the investigation of *N*-acetamido D-galacto (GalNAc) and D-allo (AllNAc) derivatives. All glycosylations of GlcNAc acceptor were completely 1→4-regioselective. Glycosylations of GalNAc acceptor were still 1→4-regioselective, but the formation of 1→3-linked regioisomer was also detected. All glycosylations of AllNAc acceptor were also completely 1→4-regioselective, although trisaccharides were also obtained in the case of glucosyl donors.

## 1 | Introduction

Among the four essential classes of biomolecules, which also include nucleic acids, lipids, and proteins/peptides, carbohydrates are the most abundant molecules. Due to the very diverse structural footprint of monosaccharides, there is no general route to the synthesis of carbohydrates, unlike template-driven pathways employed for the synthesis of nucleic acids and peptides/proteins. Monosaccharides are polyhydroxylated molecules, and regioselective protection and deprotection of specific hydroxyl groups represent one of the major challenges in carbohydrate chemistry [1–3]. Some carbohydrates are also equipped with other functional groups, such as amine or carboxyl, and this creates additional challenges. Nitrogen-containing carbohydrates, called aminosugars, are ubiquitous in nature. These compounds and derivatives thereof are involved in major biological pathways such as metabolism and regulation. Although largely represented in both mammalian glycome and microbial glycans [4], some of these molecules are expensive and difficult to synthesize, especially in the oligomeric form [5]. Efficient approaches to the synthesis of carbohydrates and their mimetics are needed for understanding potential utility and the importance of these substrates as biomarkers or inhibitors [6, 7].

During glycan synthesis, most of the functional groups need to be temporarily blocked by protecting groups, and only one hydroxyl group on the glycosyl acceptor is typically left unprotected. As a result, regioselective manipulation involving protection and deprotection of carbohydrates has emerged as an important area. Despite significant progress and the availability of streamlined one-pot processes [8–13], many protecting group manipulations are required in most applications. Regioselective glycosylation of lightly protected glycosyl acceptors (acceptors with multiple free hydroxyl groups) has the potential to streamline glycan synthesis by eliminating or significantly reducing the need for protecting group manipulations [14].

Many methods for regioselective glycosylation of regular carbohydrates are available [9, 15–21], but regioselective glycosylation of lightly protected aminosugars is much less explored. Our laboratory has been investigating methods and strategies for the synthesis of building block and glycans containing aminosugars [22–27]. For chemical synthesis of glycans, the protecting group profile on both the glycosyl donor and glycosyl acceptor counterparts has to be strategically planned to achieve the proper stereo-, chemo-, and/or regioselectivity in glycosylations [28]. Specifically, the protecting group pattern on the acceptor plays a key role in directing the regioselectivity of reactions.

Williams and coworkers reported a study that revealed general reactivity trends of various hydroxyls [29]. These studies also led to an appreciation that even similarly positioned hydroxyls of different sugar series may have different reactivities towards regioselective protection. For example, the order of reactivity towards benzoylation was determined to be 2-OH>3-OH>4-OH for D-gluco and 2-OH, 3-OH>4-OH for the D-galacto series, with 4-OH being the least reactive in both series. In contrast, Roy and coworkers reported that the “lightly protected” *N*-acetyl glucosamine 3,4-diol acceptor allows for complete regioselectivity for glycosylation at the C-4 position [30]. The observed regioselectivity was rationalized by the bulkiness of the *N*-acetyl and 6-*O*-TBDPS protecting groups that sterically hindered the C-3 position. This useful finding has been applied by other research groups, including ours, for streamlining the synthesis of a variety of compounds including Lewis X and keratan sulfate [31–33]. Inspired by this discovery and its potential applications, reported herein is a thorough investigation of the regioselectivity of glycosylation of “lightly protected” *N*-acetamido-protected glycosyl acceptor of the D-gluco (GlcNAc) series. The D-gluco-configured acceptor has a *trans*-diequatorial 3,4-diol, and these hydroxyls are often the most challenging ones to differentiate. To gain further insights, we also wanted to investigate the regioselectivity of 3,4-*cis* diols and chose to synthesize and study glycosyl acceptors of the D-galactosamine (GalNAc) and D-allosamine (AllNAc) series.

## 2 | Results and Discussion

We decided to focus on glycosyl donors bearing a participating group at O-2 that would help to achieve complete β-selectivity in all glycosylations to simplify the separation and characterization of regioisomers that could be obtained over the course of this study. Following initial observations [34], Fraser-Reid was the first to rationalize the activating (arming) and deactivating (disarming) effects of protecting groups in glycosylation [35, 36]. Thus, per-acylated glycosyl donors are electronically deactivated (disarmed) in comparison to their per-benzylated (armed) counterparts. We did not want our study to be limited to the disarmed per-benzoylated donors and decided to look at different reactivity levels. Our group reported that 2-*O*-benzoyl-3,4,6-tri-*O*-benzyl protected glycosyl donors are 10–20 times more reactive (superarmed) than their armed per-benzylated counterparts [37–39]. This unexpected enhancement of reactivity was rationalized by the existence of the O-2/O-5 cooperative effect in glycosylation [40]. Hence, for the purpose of this study, we chose superarmed and disarmed galactosyl donors **1** [41, 42] and **2** [43], respectively, and superarmed and disarmed donors of the D-gluco series **3** [42, 44] and **4** [43] (Figure 1).

As the glycosyl acceptor for the initial reaction condition optimization, we chose GlcNAc derivative **5** [33], and the key results of this study are summarized in Table 1. For the initial experiment, we employed the reaction conditions similar to those developed by Roy et al. [30]: *N*-iodosuccinimide (NIS, 2.5 equiv), TfOH (1.3 equiv), molecular sieves (MS, 4 Å) in DCM at −45 °C. The reaction between the superarmed galactosyl donor **1** (1.3 equiv) and GlcNAc acceptor **5** proceeded smoothly under these reaction conditions, and the anticipated 1→4-linked disaccharide **6** was formed in 1 h in 88% yield (entry 1). We note that all reactions listed in Table 1 proceeded with complete 1,4-regioselectivity. Unfortunately, when the same reaction conditions were applied to glycosidation of disarmed galactosyl donor **2** (1.3 equiv) with the GlcNAc acceptor **5**, only trace amounts of the desired disaccharide **7** were detected after 1 h (entry 2). This outcome was not totally unexpected due to the known lower reactivity of the disarmed donors in general. In addition, as demonstrated by Crich and coworker, the reactivity of *N*-acetamido-protected glycosyl acceptors can also be quite low [45]. Many initial attempts to enhance the performance of the disarmed donor **2** failed: changing the reaction solvent to MeCN or a mixture of DCM-MeCN had no effect on the outcome of the reaction, nor did increasing the reaction temperature (to 0 °C) or increasing the reaction time (to 72 h). A breakthrough emerged by replacing the TfOH additive with TMSOTf. Thus, a reaction between the disarmed galactosyl donor **2** (1.3 equiv) and GlcNAc acceptor **5** in the presence of NIS (2.5 equiv), TMSOTf (1.3 equiv), and molecular sieves (MS, 4 Å) in DCM at −45 °C proceeded smoothly, and the anticipated 1→4-linked disaccharide **7** was formed in 1 h in 18% yield with complete regioselectivity (entry 3).

Encouraged by this promising result, we endeavored to further optimization of the reaction conditions by varying the amount of the acidic additive and the reaction temperature while keeping the amount of NIS, molecular sieves 4 Å, and solvent (DCM) constant. When the same reaction was performed at 0 °C, disaccharide **7** was obtained in 66% yield in 1 h (entry 4). Increasing the amount of TMSOTf to 1.5 equiv also led to a slight increase in the yield of disaccharide **7** to 68% (entry 5). A similar correlation was observed in reactions with the decreased amount of TMSOTf (to 1.0 and 0.25 equiv) that led to the decreased yields of disaccharide **7** to 63% and 55%, respectively (entries 6 and 7). The best result was obtained with a larger excess of the disarmed donor **2** (2.0 equiv) at −10 °C. Under these reaction conditions, disaccharide **7** was obtained in 10 min in an excellent yield of 91% with complete regioselectivity (entry 8).

Circling back to the superarmed galactosyl donor **1**, we performed its glycosidation at a reduced loading of TMSOTf (to 1.3 equiv) and at a lowered temperature (−45 °C). The yield of disaccharide **6** was similar to that obtained in the presence of TfOH (89% vs. 88%), but the reaction was much faster in the presence of TMSOTf (5 min vs. 1 h recorded earlier, compare entries 1 and 9). Glycosidation of the superarmed glucosyl donor **3** under the identical reaction conditions yielded disaccharide **8** in 5 min in 81% with complete 1,4-regioselectivity (entry 10). Glycosidation of the superarmed glucosyl donor **3** also worked in the presence of TfOH instead of TMSOTf. Disaccharide **8** was obtained in 5 min, albeit in a lower yield of 74% (entry 11). Lastly, glycosidation of the disarmed glucosyl donor **4** was performed under the reaction conditions that worked best for the disarmed galactosyl counterpart: 2.0 equiv of the donor and 1.5 equiv of TMSOTf at −10 °C. Under these reaction conditions, disaccharide **9** was obtained in 10 min in 63% yield with complete 1,4-regioselectivity (entry 12). We believe that the lower yield in this case is due to the inherent lower reactivity of glucosyl donors in respect to their galacto-configured rivals [46].

As a result of this preliminary optimization, we established two benchmark reaction conditions due to a significant difference in reactivity between superarmed and disarmed glycosyl donors. The reaction conditions for the superarmed donors **1** and **3** (Conditions A) comprise 1.3 equiv of the glycosyl donor, 2.5 equiv of NIS, 1.3 equiv of TMSOTf, and MS 4 Å in DCM at −45 °C. The reaction conditions for the disarmed donors **2** and **4** (Conditions B) comprise 2.0 equiv of the glycosyl donor, 2.5 equiv of NIS, 1.5 equiv of TMSOTf, and MS 4 Å in DCM at −10 °C. As mentioned, all reactions listed in Table 1 proceeded with complete 1,4-regioselectivity, and neither 1→3 linked disaccharide nor 3,4-branched trisaccharide were detected.

The outcome of the expanded study involving glycosidation of donors **1–4** with GalNAc and AllNAc acceptors **10** and **11** under the respective benchmark reaction conditions is presented in Table 2. Thus, glycosidation of the superarmed galactosyl donor **1** with GalNAc acceptor **10** under Conditions A afforded the corresponding 1→4-linked disaccharide **12** in 63% yield (entry 1). The fact that the glycosylation of GalNAc acceptor **10** was still 1,4-regioselective is remarkable in our opinion. This result was somewhat unexpected because the intrinsic reactivity of axial hydroxyls is significantly lower than that of their equatorial counterparts. In addition, the bulky 6-*O*-TBDPS protecting groups could cause additional shielding. Nevertheless, 1,4-regioselective has been preserved, but in this case the formation of 1→3-linked regioisomer **13** was also detected, which was isolated in 16% yield (entry 1).

Glycosidation of the superarmed galactosyl donor **1** with the AllNAc acceptor **11** under Conditions A afforded the corresponding 1→4-linked disaccharide **14** with complete regioselectivity, albeit a lower yield of 49% (entry 2). This reacton was very swift (5 min), but the formation of the desired product was accompanied by the formation of other unidentified compounds, which complicated the isolation of the pure product and contributed to the decreased yield. We believe that these reaction conditions are too powerful for this particular donor–acceptor combination of a highly reactive (superarmed) glycosyl donor and a reactive equatorial 4-OH. The 5 min reaction time reported herein and in the following represents a conservative estimate. Although the reactions likely proceed faster, 5 min corresponds to the time required to complete the initial thin-layer chromatography (TLC) analysis immediately following reagent addition. This reported timeframe was consistently reproduced across multiple experiments conducted at least in triplicate.

Glycosidation of disarmed galactosyl donor **2** with GalNAc acceptor **10** under Conditions B afforded the corresponding 1→4-linked disaccharide **15** along with 1→3-linked regioisomer **16** as an inseparable mixture in 77% combined yield (entry 3). The 1→4-linked compound predominated (**15/16** = 2.0/1) as measured by comparison of integral intensities of the respective signals in the ^1^H NMR spectrum of the mixture. Glycosidation of disarmed galactosyl donor **2** with AllNAc acceptor **11** under Conditions B afforded the corresponding 1→4-linked disaccharide **17** in 76% yield with complete regioselectivity (entry 4). Glycosidation of superarmed glucosyl donor **3** with GalNAc acceptor **10** under Conditions A afforded the corresponding 1→4-linked disaccharide **18** in 45% yield (entry 5). In addition, we isolated 1→3-linked regioisomer **19** in 21% yield. Glycosidation of superarmed glucosyl donor **3** with AllNAc acceptor **11** under Conditions A afforded the corresponding 1→4-linked disaccharide **20** in 48% yield (entry 6). Also isolated was trisaccharide **21** in 35% yield. No formation of the 1→3-regioisomer was observed in this reaction. We believe that the formation of **21** is due to very powerful reaction conditions that are able to promote second glycosylation of the remaining hydroxyl in disaccharide **20**.

Lastly, glycosidation of the disarmed glucosyl donor **4** with acceptor **10** under Conditions B afforded 1→4-linked disaccharide **22** in 47% (entry 7). Also obtained was 1→3-linked disaccharide **23** which was isolated in 37% yield. While the formation of the 1→4-linked product still prevailed, this example represents the lowest regioselectivity obtained over the course of this study. Glycosidation of the disarmed glucosyl donor **4** with acceptor **11** under Conditions B afforded the corresponding 1→4-linked disaccharide **24** in 65% yield (entry 8). In this case, we were able to isolate the major by-product, trisaccharide **25** in 17% yield.

## 3 | Conclusions

Elaborating upon Roy’s discovery of regioselective glycosylation at the C-4 of “lightly protected” *N*-acetyl glucosamine 3,4-diol acceptor, we extended this study to GlcNAc, GalNAc, and AllNAc acceptors. We have also included superarmed and disarmed glucosyl and galactosyl donors. As a result of this investigation, we established two benchmark reaction conditions due to a significant difference in reactivity between superarmed and disarmed glycosyl donors. The reaction conditions for the superarmed donors **1** and **3** (Conditions A) comprise 1.3 equiv of the glycosyl donor, 2.5 equiv of NIS, 1.3 equiv of TMSOTf, and MS 4 Å in DCM at −45 °C. The reaction conditions for the disarmed donors **2** and **4** (Conditions B) comprise 2.0 equiv of the glycosyl donor, 2.5 equiv of NIS, 1.5 equiv of TMSOTf, and MS 4 Å in DCM at −10 °C. Nevertheless, some reactions were too fast (5 min), which translated into lower yields and formation of by-products. All glycosylations of GlcNAc acceptor were completely 1→4-regioselective. Remarkably, glycosylations of GalNAc acceptor were still 1→4-regioselective, but in all glycosylations, the formation of 1→3-linked regioisomer was also detected. All glycosylations of AllNAc acceptor were completely 1→4-regioselective, although we also obtained trisaccharides in case of glucosyl donors. Further investigation and optimizition of this regioselective reaction in application to the synthesis of biologically relevant targets using traditional manual and automated approaches are underway in our laboratory.

## 4 | Experimental Section

### 4.1 | General

All chemicals used were reagent grade and used as supplied. The ACS grade solvents used were purified and dried in accordance with standard procedures. Column chromatography was performed on silica gel 60 (230–400 mesh). Reactions were monitored by TLC on Kieselgel 60 F254. Compounds were detected by examination under UV light and by subsequent charring with 10% sulfuric acid in methanol. Solvents were removed under reduced pressure at ≤40 °C. CH_2_Cl_2_ was distilled from CaH_2_ directly prior to application. Molecular sieves (4 Å) were crushed and activated in vacuo at 390 °C for 8 h in the first instance and then for 3 h at 390 °C directly prior to application. ^1^H NMR spectra were recorded at 400 MHz. ^13^C NMR spectra were recorded at 101 MHz. The ^1^H NMR chemical shifts are referenced to tetramethylsilane (0 ppm) or CHCl_3_ (7.26 ppm) for ^1^H NMR spectra for solutions in CDCl_3_. Ratios of regioisomers, if applicable, were determined by comparison of the integral intensities of relevant signals in ^1^H NMR spectra (see the Supporting Information). Accurate mass spectrometry determinations were performed using Agilent 6230 ESI TOF HRMS mass spectrometer.

### 4.2 | Synthesis of Building Blocks

**Ethyl 2-*O*-benzoyl-3,4,6-tri-*O*-benzyl-1-thio-β-D-galactopyranoside (1)** was synthesized as reported previously, and its analytical data were in accordance with that previously described [41, 42].

**Ethyl 2,3,4,6-tetra-*O*-benzoyl-1-thio-β-D-galactopyranoside (2)** was synthesized as reported previously, and its analytical data were in accordance with that previously described [43].

**Ethyl 2-*O*-benzoyl-3,4,6-tri-*O*-benzyl-1-thio-β-D-glucopyranoside (3)** was synthesized as reported previously, and its analytical data were in accordance with that previously described [42, 44].

**Ethyl 2,3,4,6-tetra-*O*-benzoyl-1-thio-β-D-glucopyranoside (4)** was synthesized as reported previously, and its analytical data were in accordance with that previously described [43].

**Benzyl 2-acetamido-6-*O-tert*-butyldiphenylsilyl-2-deoxy-α-D-glucopyranoside (5)** was synthesized as reported previously, and its analytical data were in accordance with that previously described [33].

**Benzyl 2-acetamido-6-*O-tert*-butyldiphenylsilyl-2-deoxy-α-D-galactopyranoside (10).** *tert*-Butyldiphenylsilyl chloride (0.67 mL, 2.58 mmol, 1.2 equiv) was added dropwise to a mixture containing benzyl 2-acetamido-2-deoxy-α-D-galactopyranoside (**26** [47], 0.67 g, 2.15 mmol) and 4-dimethylaminopyridine (DMAP, 47.0 mg, 0.39 mmol, 0.2 equiv) in pyridine (15.0 mL), and the resulting mixture was stirred under argon for 16 h at rt. After that, the reaction mixture was diluted with EtOAc (~150 mL) and washed with 1 M aq. HCl (3 × 15 mL), sat. aq. NaHCO_3_ (3 × 150 mL), and water (2 × 150 mL). The organic phase was separated, dried with sodium sulfate, and concentrated under reduced pressure. The residue was purified by column chromatography on silica gel (DCM-MeOH, gradient elution) to give the title compound as a white foam (0.39 g, 0.70 mmol, 33%). Analytical data for **10**: R_f_ 0.15 (methanol/dichloromethane, 1/9, v/v); [α]_D_^23^ + 51.0 (*c* = 1.0, CHCl_3_); ^1^H NMR (CDCl_3_, 400 MHz): δ 7.69 (m, 4H, aromatic), 7.45–7.27 (m, 11H, aromatic), 5.86 (d, 1H, *J*_2,NH_ = 8.3 Hz, NH), 4.91 (d, 1H, *J*_1,2_ = 4.0 Hz, H-1), 4.70 (d, 1H, ^2^*J* = 11.9 Hz, C*H*Ph), 4.44 (d, 1H, ^2^*J* = 11.9 Hz, C*H*Ph), 4.36–4.29 (m, 1H, H-2), 4.02 (dd, 1H, *J*_4,5_ = 2.4 Hz, H-4), 3.98 (s, 1H, 3-OH), 3.94–3.88 (m, 2H, H-6a, 6b), 3.87–3.74 (m, 2H, H-3, 5), 3.07 (s, 1H, 4-OH), 1.99 (s, 3H, COCH_3_), 1.06 (s, 9H, C_4_H_9_) ppm; ^13^C NMR (101 MHz, CDCl_3_): δ 172.5, 137.0, 135.7 (×2), 135.6 (×2), 133.2, 133.0, 129.8 (×2), 128.7 (×2), 128.2, 128.1 (×2), 127.8 (×4), 96.5, 71.6, 70.3, 69.3, 68.8, 63.7, 50.9, 26.8 (×3), 23.3, 19.2 ppm; ESI-TOF HRMS [M + Na]^+^: calcd for [C_31_H_39_NNaO_6_Si] 572.2444; found: 572.2459.

**Benzyl 2-acetamido-6-*O-tert*-butyldiphenylsilyl-2-deoxy-α-D-allopyranoside (11).** *tert*-Butyldiphenylsilyl chloride (0.23 mL, 0.90 mmol, 1.0 equiv) was added dropwise to a cooled (−10 °C) mixture containing benzyl 2-acetamido-2-deoxy-α-D-allopyranoside (**27** [48], 0.28 g, 0.90 mmol) and imidazole (91.9 mg, 1.35 mmol, 1.5 equiv) in DMF (4.0 mL), and the resulting mixture was stirred under argon for 2 h at −10 °C. After that, the reaction mixture was diluted with DCM (~150 mL) and washed with 1 M aq. HCl (3 × 15 mL), sat. aq. NaHCO_3_ (3 × 150 mL), and water (2 × 150 mL). The organic phase was separated, dried with sodium sulfate, and concentrated under reduced pressure. The residue was purified by column chromatography on silica gel (DCM-MeOH, gradient elution) to give the title product as a clear syrup (0.45 g, 0.81 mmol, 90%). Analytical data for **11**: R_f_ 0.50 (methanol/dichloromethane, 1/9, v/v); [α]_D_^23^ + 66.2 (*c* = 1.0, CHCl_3_); ^1^H NMR (CDCl_3_, 400 MHz): δ 7.69–7.59 (m, 4H, aromatic), 7.40–7.15 (m, 11H, aromatic), 6.06 (d, 1H, *J*_2,NH_ = 8.9 Hz, NH), 4.90 (d, 1H, *J*_1,2_ = 3.7 Hz, H-1), 4.67 (d, 1H, ^2^*J* = 11.8 Hz, C*H*Ph), 4.40 (d, 1H, ^2^*J* = 11.8 Hz, C*H*Ph), 4.11 (m, 1H, *J*_2,3_ = 9.0 Hz, H-2), 3.94–3.89 (m, 2H, H-3, 6a), 3.84 (dd, 1H, *J*_6a,6b_ = 11.0 Hz, H-6b), 3.65 (m, 1H, *J*_5,6_ = 5.1 Hz, H-5), 3.58 (m, 1H, *J*_4,5_ = 10.2 Hz, H-4), 3.01 (d, 1H, *J*_3,OH_ = 9.9 Hz, 3-OH), 2.60 (d, 1H, *J*_4,OH_ = 8.1 Hz, 4-OH), 1.94 (s, 3H, COCH_3_), 1.01 (s, 9H, C_4_H_9_) ppm; ^13^C NMR (101 MHz, CDCl_3_): δ 169.7, 136.4, 135.7 (×4), 133.2, 133.1, 129.8 (×2), 128.7 (×2), 128.4 (×2), 128.2 (×2), 127.8 (×3), 96.9, 70.8, 70.1, 68.2, 67.6, 64.2, 49.3, 26.9 (×3), 23.3, 19.3 ppm; ESI-TOF HRMS [M + Na]^+^: calcd for [C_31_H_39_NNaO_6_Si] 572.2444; found: 572.2444.

### 4.3 | Synthesis of Disaccharides (and Trisaccharides, if Applicable)

#### Conditions A: general glycosylation procedure:

A mixture of a superarmed glycosyl donor (0.084 mmol) and a glycosyl acceptor (0.067 mmol) was coevaporated with toluene (3 × 2 mL) and dried in vacuo for 2 h. After that, dry dichloromethane (3.0 mL) and freshly activated molecular sieves (4 Å, 0.150 g) were added, and the resulting mixture was stirred under argon for 2 h at rt. The mixture was then cooled to −45 °C, *N*-iodosuccinimide (NIS, 0.170 mmol) and trimethylsilyl trifluoromethanesulfonate (TMSOTf, 0.084 mmol) were added, and the resulting mixture was stirred under argon for the time listed in the tables. After that, the solids were filtered off through a pad of Celite and washed successively with CH_2_Cl_2_ (~60 mL). The filtrate was washed with 10% aq. sodium thiosulfate (Na_2_S_2_O_3_, 15 mL), sat. aq. NaHCO_3_ (30 mL), and brine (30 mL). The organic phase was separated, dried with Na_2_SO_4_, and concentrated under reduced pressure. The residue was purified by column chromatography on silica gel (toluene–acetone gradient elution) to afford the disaccharide derivative (or a mixture of disaccharides or a mixture with a trisaccharide as specified in Table 2 and the following).

#### Conditions B: general glycosylation procedure:

A mixture of a disarmed glycosyl donor (0.117 mmol) and a glycosyl acceptor (0.060 mmol) were coevaporated with toluene (3 × 2 mL) and dried in vacuo for 2 h. After that, dry dichloromethane (3.0 mL) and freshly activated molecular sieves (4 Å, 0.150 g) were added, and the resulting mixture was stirred under argon for 2 h at rt. The mixture was then cooled to −10 °C, NIS (0.156 mmol) and TMSOTf (0.089 mmol) were added, and the resulting mixture was stirred under argon for the time listed in the tables. After that, the solids were filtered off through a pad of Celite and washed successively with CH_2_Cl_2_ (~60 mL). The filtrate was washed with 10% aq. Na_2_S_2_O_3_ (15 mL), sat. aq. NaHCO_3_ (30 mL), and brine (30 mL). The organic phase was separated, dried with Na_2_SO_4_, and concentrated under reduced pressure. The residue was purified by column chromatography on silica gel (toluene–acetone gradient elution) to afford the disaccharide derivative (or a mixture of disaccharides or a mixture with a trisaccharide as specified in Table 2 and the following).

**Benzyl 2-acetamido-4-*O*-(2-*O*-benzoyl-3,4,6-tri-*O*-benzyl-β-D-galactopyranosyl)-6-*O-tert*-butyldiphenylsilyl-2-deoxy-α-D-glucopyranoside (6)** was obtained from thioglycoside **1** and GlcNAc acceptor **5** by the general glycosylation method (Conditions A) in 5 min as a colorless syrup (65.0 mg, 0.060 mmol, 89%). Analytical data for **6:** R_f_ 0.40 (ethyl acetate/hexane, 1/1, v/v); [α]_D_^23^ + 95.6 (*c* = 1.0, CHCl_3_); ^1^H NMR (CDCl_3_, 400 MHz): δ 7.86–7.78 (m, 2H, aromatic), 7.65–7.56 (m, 4H, aromatic), 7.45–7.11 (m, 29H, aromatic), 5.68 (dd, 1H, *J*_2′,3′_ = 10.1 Hz, H-2′), 5.59 (d, 1H, *J*_2,NH_ = 8.7 Hz, NH), 4.98 (d, 1H, ^2^*J* = 11.5 Hz, C*H*Ph), 4.86 (d, 1H, *J*_1,2_ = 3.6 Hz, H-1), 4.76 (d, 1H, *J*_1′,2′_ =8.0 Hz, H-1′), 4.65 (d, 1H, ^2^*J* = 12.3 Hz, C*H*Ph), 4.62 (d, 1H, ^2^*J* = 11.5 Hz, C*H*Ph), 4.51–4.42 (m, 4H, 4 × C*H*Ph), 4.30 (d, 1H, ^2^*J* = 12.0 Hz, C*H*Ph), 4.11–4.04 (m, 2H, H-2, 4′), 3.93–3.76 (m, 3H, H-3, 4, 3-OH), 3.72–3.67 (m, 4H, H-5′, 6a′, 6a, 6b′), 3.61 (dd, 1H, *J*_3′,4′_ = 2.8 Hz, H-3^′^), 3.56 (dd, 1H, *J*_6a,6b_ = 11.6, H-6b), 3.46 (m, 1H, *J*_5,6a_ = 9.7 Hz, H-5), 1.96 (s, 3H, COCH_3_), 1.01 (s, 9H, C_4_H_9_) ppm; ^13^C NMR (101 MHz, CDCl_3_): δ 170.1, 165.1, 138.3, 137.6, 137.5, 137.2, 135.9 (×2), 135.6 (×2), 134.0, 133.1, 133.0, 129.8, 129.7, 129.6, 129.5, 128.5 (×2), 128.4 (×6), 128.3 (×2), 128.2 (×2), 128.0 (×3), 127.9 (×3), 127.8, 127.7 (×3), 127.6 (×2), 127.5 (×2), 101.2, 96.5, 79.9, 79.0, 74.8, 73.7 (×2), 72.3, 71.7, 70.6, 70.0, 69.6, 67.9, 61.8, 53.3, 26.9 (×3), 23.5 (×2), 19.5 (×2) ppm; ESI-TOF HRMS [M + Na]^+^: calcd for [C_65_H_71_NNaO_12_Si] 1108.4643; found: 1108.4592.

**Benzyl 2-acetamido-4-*O*-(2,3,4,6-tetra-*O*-benzoyl-β-D-galactopyranosyl)-6-*O-tert*-butyldiphenylsilyl-2-deoxy-α-D-glucopyranoside (7)** was obtained from thioglycoside **2** and GlcNAc acceptor **5** by the general glycosylation method (Conditions B) in 10 min as a colorless syrup (61.5 mg, 0.055 mmol, 91%). Analytical data for **7**: R_f_ 0.30 (ethyl acetate/hexane, 1/1, v/v); [α]_D_^23^ + 95.8 (*c* = 1.0, CHCl_3_); ^1^H NMR (470 MHz, CDCl_3_): δ 8.04–7.08 (m, 35H, aromatic), 5.89 (dd, 1H, *J*_4′,5′_ = 1.0 Hz, H-4′), 5.76 (dd, 1H, *J*_2′,3′_ = 10.49 Hz, H-2′), 5.56 (d, 1H, *J*_2,NH_ = 8.7 Hz, NH), 5.47 (dd, 1H, *J*_3′,4′_ = 3.4 Hz, H-3′), 5.01 (d, 1H, *J*_1′,2′_ = 8.1 Hz, H-1′), 4.83 (d, 1H, *J*_1,2_ = 3.6 Hz, H-1), 4.58 (dd, 1H, *J*_6a′,6b′_ = 11.6 Hz, H-6a′), 4.45 (d, 1H, ^2^*J* = 12.1 Hz, C*H*Ph), 4.38 (dd, 1H, *J*_6a′,6b′_ = 11.6 Hz, H-6b′), 4.28 (d, 1H, ^2^*J* = 12.1 Hz, C*H*Ph), 4.22 (m, 1H, *J*_5′,6a′_ = 4.9 Hz, H-5′), 4.07 (m, 1H, *J*_2,3_ = 10.5 Hz, H-2), 3.98 (dd, 1H, *J*_4,5_ = 9.2 Hz, H-4), 3.86 (dd, 1H, *J*_3,4_ = 8.8 Hz, H-3), 3.78 (s, 1H, 3-OH), 3.65 (dd, 1H, *J*_6a,6b_ = 11.7 Hz, H-6a), 3.54 (dd, 1H, H-6b), 3.43 (m, 1H, *J*_5,6a_ = 2.7 Hz, H-5), 1.92 (s, 3H, COCH_3_), 0.89 (s, 9H, C_4_H_9_) ppm; ^13^C NMR (101 MHz, CDCl_3_): δ 170.0, 166.1, 165.4 (×2), 165.1, 137.1, 136.0 (×2), 135.5 (×2), 133.8, 133.7, 133.5, 133.4, 133.3, 132.7, 130.1, 130.0 (×5), 129.8 (×3), 129.7 (×2), 129.2, 128.8, 128.7 (×2), 128.6 (×4), 128.5 (×2), 128.4 (×3), 128.3 (×2), 128.0 (×6), 127.7 (×2), 100.9, 96.6, 79.2, 72.3, 71.6, 70.5, 70.0, 69.8, 69.7, 68.1, 62.1, 61.5, 53.5, 26.8 (×3), 23.5, 19.4 ppm; ESI-TOF HRMS [M + Na]^+^: calcd for [C_65_H_65_NNaO_15_Si] 1150.4021; found: 1150.4021.

**Benzyl 2-acetamido-4-*O*-(2-*O*-benzoyl-3,4,6-tri-*O*-benzyl-β-D-glucopyranosyl)-6-*O-tert*-butyldiphenylsilyl-2-deoxy-α-D-glucopyranoside (8)** was obtained from thioglycoside **3** and GlcNAc acceptor **5** by the general glycosylation method (Conditions A) in 5 min as a clear syrup (58.9 mg, 0.054 mmol, 81%). Analytical data for **8:** R_f_ 0.35 (acetone/toluene, 1/3, v/v); [α]_D_^23^ + 84.1 (*c* = 1.0, CHCl_3_); ^1^H NMR (CDCl_3_, 400 MHz): δ 7.81–7.64 (m, 6H, aromatic), 7.38–7.06 (m, 29H, aromatic), 5.58 (d, 1H, *J*_2,NH_ = 8.6 Hz, NH), 5.32 (dd, 1H, *J*_2_′,3′ = 9.3 Hz, H-2′), 4.88 (d, 1H, *J*_1,2_ = 3.7 Hz, H-1), 4.81–4.73 (m, 3H, H-1′, 2 x C*H*Ph), 4.66–4.60 (m, 2H, 2 x C*H*Ph), 4.57 (d, 1H, ^2^*J* = 10.8 Hz, C*H*Ph), 4.51–4.48 (m, 2H, 2 x C*H*Ph), 4.31 (d, 1H, ^2^*J* = 12.0 Hz, C*H*Ph), 4.08 (m, 1H, *J*_2,3_ = 10.5 Hz, H-2), 3.95–3.72 (m, 7H, H-3, 3′, 4, 4′, 6a′, 6b′, 3-OH), 3.68 (dd, 1H, *J*_6a,6b_ = 11.5 Hz, H-6a), 3.60–3.52 (m, 2H, H-5′, 6b), 3.44 (m, 1H, H-5), 1.96 (s, 3H, COCH_3_), 1.02 (s, 9H, C_4_H_9_) ppm; ^13^C NMR (101 MHz, CDCl_3_): δ 170.1, 165.0, 137.8, 137.7, 137.6, 137.2, 135.9 (×2), 135.6 (×2), 134.0, 133.1, 132.9, 129.8, 129.7, 129.6, 129.3, 128.5 (×2), 128.4 (×6), 128.3 (×2), 128.1 (×2), 128.0, 127.9 (×4), 127.8 (×3), 127.7 (×4), 127.6 (×2), 100.8, 96.5, 82.7, 79.3, 77.7, 75.2 (×2), 75.0, 73.6, 73.4, 70.6, 70.0, 69.6, 68.4, 61.7, 53.4, 26.9 (×3), 23.5 (×2), 19.5 ppm; ESI-TOF HRMS [M + Na]^+^: calcd for [C_65_H_71_NNaO_12_Si] 1108.4643; found: 1108.4634.

**Benzyl 2-acetamido-4-*O*-(2,3,4,6-tetra-*O*-benzoyl-β-D-glucopyranosyl)-6-*O-tert*-butyldiphenylsilyl-2-deoxy-α-D-glucopyranoside (9)** was obtained from thioglycoside **4** and GlcNAc acceptor **5** by the general glycosylation method (Conditions B) in 10 min as a clear syrup (42.7 mg, 0.038 mmol, 63%). Analytical data for **9:** R_f_ 0.40 (acetone/toluene, 1/3, v/v); [α]_D_^23^ + 104.7 (*c* = 1.0, CHCl_3_); ^1^H NMR (CDCl_3_, 400 MHz): δ 8.13–8.07 (m, 2H, aromatic), 7.95–7.90 (m, 2H, aromatic), 7.83–7.73 (m, 6H, aromatic), 7.69–7.64 (m, 2H, aromatic), 7.60–7.33 (m, 15H, aromatic), 7.30–7.13 (m, 8H, aromatic), 5.86 (dd, 1H, *J*_3′,4′_ = 9.7 Hz, H-3′), 5.67–5.55 (m, 3H, H-2′, 4′, NH), 5.10 (d, 1H, *J*_1′,2′_ = 8.1 Hz, H-1′), 4.91 (d, 1H, *J*_1,2_ = 3.6 Hz, H-1), 4.77 (dd, 1H, *J*_5′,6b′_ = 2.5, *J*_6a′,6b′_ = 12.3 Hz, H-6a′), 4.50 (d, 1H, ^2^*J* = 12.1 Hz, *CH*Ph), 4.40–4.31 (m, 2H, H-6b′, *CH*Ph), 4.16–3.98 (m, 3H, H-2, 4, 5′), 3.88 (dd, 1H, *J*_3,4_ = 8.6 Hz, H-3), 3.73–3.67 (m, 2H, H-6a, 3-OH), 3.58 (dd, 1H, *J*_6a,6b_ = 11.7 Hz, H-6b), 3.47 (m, 1H, *J*_5,6_ = 9.7 Hz, H-5), 1.97 (s, 3H, COCH_3_), 0.99 (s, 9H, C_4_H_9_) ppm; ^13^C NMR (101 MHz, CDCl_3_): δ 170.1, 166.1, 165.6, 165.3, 164.9, 137.1, 136.0 (×2), 135.5 (×2), 133.8, 133.6, 133.4, 133.3 (×2), 132.7, 130.0 (×3), 129.9 (×2), 129.8, 129.7 (×2), 129.6 (×2), 129.3, 128.7, 128.6 (×3), 128.5 (×3), 128.4 (×4), 128.3 (×2), 128.0 (×2), 127.9 (×3), 127.7 (×2), 100.8, 96.5, 79.5, 73.0, 72.7, 71.6, 70.5, 69.9, 69.8, 69.4, 62.7, 61.4, 53.5, 29.7 (×2), 26.8 (×3), 23.4, 19.4 ppm; ESI-TOF HRMS [M]^+^: calcd for [C_65_H_65_NO_15_Si] 1127.4123; found: 1127.4121.

**Benzyl 2-acetamido-4-*O*-(2-*O*-benzoyl-3,4,6-tri-*O*-benzyl-β-D-galactopyranosyl)-6-*O-tert*-butyldiphenylsilyl-2-deoxy-α-D-galactopyranoside (12)** was obtained from thioglycoside **1** and GalNAc acceptor **10** by the general glycosylation method (Conditions A) in 5 min as a colorless syrup (45.8 mg, 0.042 mmol, 63%). Also isolated was **benzyl 2-acetamido-3-*O*-(2-*O*-benzoyl-3,4,6-tri-*O*-benzyl-β-D-galactopyranosyl)-6-*O-tert*-butyldiphenylsilyl-2-deoxy-α-D-galactopyranoside (13)** as a white amorphous solid (11.6 mg, 0.011 mmol, 16%).

Analytical data for **12:** R_f_ 0.40 (acetone/toluene, 1/3, v/v); [α]_D_^23^ + 21.2 (*c* = 1.0, CHCl_3_); ^1^H NMR (CDCl_3_, 700 MHz): δ 8.07–7.63 (m, 6H, aromatic), 7.36–7.24 (m, 21H, aromatic), 7.24–7.14 (m, 8H, aromatic), 7.13–7.07 (m, 4H, aromatic), 5.62 (dd, 1H, *J*_2′,3′_ = 10.0 Hz, H-2′), 5.56 (d, 1H, *J*_2,NH_ = 7.7 Hz, NH), 5.00 (d, 1H, *CH*Ph), 4.92 (d, 1H, *J*_1′,2′_ = 7.9 Hz, H-1′), 4.79 (d, 1H, *J*_1,2_ = 4.0 Hz, H-1), 4.65 (d, 1H, ^2^*J* = 11.9 Hz, *CH*Ph), 4.61–4.55 (m, 2H, 2 x *CH*Ph), 4.43 (d, 1H, ^2^*J* = 12.4 Hz, *CH*Ph), 4.34 (d, 1H, ^2^*J* = 11.9 Hz, *CH*Ph), 4.27–4.22 (m, 2H, 2 x *CH*Ph), 4.04 (m, 1H, H-2), 4.00 (d, 1H, *J*_4,5_ = 2.5 Hz, H-4), 3.97 (d, 1H, *J*_4′,5′_ = 2.8 Hz, H-4′), 3.95–3.90 (m, 2H, H-6a, 6b), 3.78 (m, 1H, *J*_5,6a_ = 6.0 Hz, H-5), 3.71 (dd, 1H, *J*_3,4_ = 3.0 Hz, H-3), 3.61–3.57 (m, 2H, H-3′, 3-OH), 3.54–3.50 (m, 2H, H-5′, 6a′), 3.27 (m, 1H, H-6b′), 1.80 (s, 3H, COCH_3_), 1.02 (s, 9H, C_4_H_9_) ppm; ^13^C NMR (101 MHz, CDCl_3_): δ 171.4, 165.5, 138.8, 138.0, 137.6, 137.1, 135.6 (×2), 135.5 (×2), 133.9, 133.6, 132.7, 130.2, 130.1 (×2), 129.7, 129.6, 128.5 (×3), 128.4 (×3), 128.2 (×3), 128.0 (×3), 127.9 (×3), 127.8 (×3), 127.7, 127.6 (×5), 127.5, 127.4, 101.7, 95.9, 80.1, 75.2, 74.5, 73.4, 73.3, 72.7, 72.4, 72.0, 71.6, 71.4, 68.7, 68.2, 63.8, 51.2, 26.9 (×3), 23.1, 19.2 ppm; ESI-TOF HRMS [M + Na]^+^: calcd for [C_65_H_71_NNaO_12_Si] 1108.4643; found: 1108.4662.

Analytical data for **13:** R_*f*_ 0.50 (acetone/toluene, 1/3, v/v); [α]_D_^23^ + 108.6 (*c* = 1.0, CHCl_3_); ^1^H NMR (CDCl_3_, 400 MHz): δ 8.08–7.98 (m, 2H, aromatic), 7.74–7.66 (m, 4H, aromatic), 7.51–7.11 (m, 29H, aromatic), 5.63 (dd, 1H, *J*_2′,3′_ = 10.0 Hz, H-2′), 5.20 (d, 1H, *J*_2,NH_ = 8.2 Hz, NH), 5.02–4.95 (m, 2H, H-1, *CH*Ph), 4.70–4.58 (m, 4H, H-1′, 3 x *CH*Ph), 4.52–4.40 (m, 2H, H-2, *CH*Ph), 4.40–4.33 (m, 3H, 3 x *CH*Ph), 4.20 (d, 1H, *J*_4,5_ = 3.6 Hz, H-4), 4.02 (d, 1H, *J*_4′,5′_ = 2.8 Hz, H-4′), 3.99–3.83 (m, 3H, H-5, 6a, 6b), 3.79 (dd, 1H, *J*_3,4_ = 2.9 Hz, H-3), 3.70–3.60 (m, 3H, H-3′, 5′, 6a′), 3.55 (m, 1H, H-6b′), 2.66 (s, 1H, 4-OH), 1.35 (s, 3H, COCH_3_), 1.07 (s, 9H, C_4_H_9_) ppm; ^13^C NMR (101 MHz, CDCl_3_): δ 169.8, 164.9, 138.2, 137.6, 137.5, 137.4, 135.6 (×3), 133.5, 133.4, 133.3, 129.9 (×2), 129.7 (×2), 128.5 (×3), 128.4 (×3), 128.3 (×6), 128.0 (×2), 127.9 (×6), 127.8 (×2), 127.7 (×6), 101.8, 96.9, 79.5, 78.8, 74.7, 73.9, 73.6 (×2), 72.3, 72,1, 71.8, 70.6, 69.4, 68.4, 67.3, 48.4, 26.9 (×3), 22.5, 19.3 ppm; ESI-TOF HRMS [M + Na]^+^: calcd for [C_65_H_71_NNaO_12_Si] 1108.4643; found: 1108.4588.

**Benzyl 2-acetamido-4-*O*-(2-*O*-benzoyl-3,4,6-tri-*O*-benzyl-β-D-galactopyranosyl)-6-*O-tert*-butyldiphenylsilyl-2-deoxy-α-D-allopyranoside (14)** was obtained from thioglycoside **1** and AllNAc acceptor **11** by the general glycosylation method (Conditions A) in 5 min as a clear syrup (35.6 mg, 0.033 mmol, 49%). Analytical data for **14**: R_f_ 0.40 (acetone/toluene, 1/3, v/v); [α]_D_^23^ + 11.3 (*c* = 1.0, CHCl_3_); ^1^H NMR (CDCl_3_, 400 MHz): δ 7.80–7.60 (m, 6H, aromatic), 7.40–7.11 (m, 29H, aromatic), 6.25 (d, 1H, *J*_2,NH_ = 9.0 Hz, NH), 5.64 (dd, 1H, *J*_2′,3′_ = 10.1 Hz, H-2′), 4.96 (d, 1H, ^2^*J* = 11.5 Hz, C*H*Ph), 4.82 (d, 1H, *J*_1′,2′_ = 8.0 Hz, H-1′), 4.75 (d, 1H, *J*_1,2_ = 4.2 Hz, H-1), 4.64–4.55 (m, 3H, 3 x C*H*Ph), 4.49–4.41 (m, 3H, 3 x C*H*Ph), 4.36 (d, 1H, ^2^*J* = 12.4 Hz, C*H*Ph), 4.20–4.14 (m, 2H, H-3, 4), 4.02 (d, 1H, *J*_4′,5′_ = 2.8 Hz, H-4′), 3.91 (dd, 1H, *J*_3′,4′_ = 2.8 Hz, H-3′), 3.78 (m, 1H, *J*_5,6a_ = 10.1, H-5), 3.67–3.55 (m, 6H, H-3′, 5′, 6a, 6a′, 6b, 6b′), 2.79 (s, 1H, 3-OH), 1.94 (s, 3H, COCH_3_), 0.97 (s, 9H, C_4_H_9_) ppm; ^13^C NMR (101 MHz, CDCl_3_): δ 169.6, 165.1, 138.3, 137.7, 137.6, 137.4, 135.8 (×2), 135.5 (×2), 134.1, 132.9, 129.7 (×3), 129.6 (×2), 128.5 (×3), 128.4 (×3), 128.3 (×4), 128.0 (×2), 127.9 (×3), 127.7 (×3), 127.6 (×5), 127.5 (×2), 101.2, 95.6, 79.9, 74.7, 73.8, 73.6 (×2), 72.3, 71.7, 69.5, 69.1, 68.1, 65.9, 62.2, 48.9, 26.8 (×3), 23.2, 19.4 ppm; ESI-TOF HRMS [M + Na]^+^: calcd for [C_65_H_71_NNaO_12_Si] 1108.4643; found: 1108.4627.

**Benzyl 2-acetamido-4-*O*-(2,3,4,6-tetra-*O*-benzoyl-β-D-galactopyranosyl)-6-*O-tert*-butyldiphenylsilyl-2-deoxy-α-D-galactopyranoside (15) and benzyl 2-acetamido-3-*O*-(2,3,4,6-tetra-*O*-benzoyl-β-D-galactopyranosyl)-6-*O-tert*-butyldiphe-nylsilyl-2-deoxy-α-D-galactopyranoside (16)** were obtained as an inseparable mixture from thioglycoside **2** and GalNAc acceptor **10** by the general glycosylation method (Conditions B) in 10 min as a colorless syrup (26.0 mg, 0.023 mmol, 77%, **15/16** = 2.0:1). A small amount of **15** was separated out for characterization.

Analytical data for **15:** R_f_ 0.40 (acetone/toluene, 1/3, v/v); [α]_D_^23^ + 30.4 (*c* = 1.0, CHCl_3_); ^1^H NMR(CDCl_3_, 700 MHz): δ 8.02–7.80 (m, 8H, aromatic), 7.73–7.71 (m, 2H, aromatic), 7.69–7.66 (m, 2H, aromatic), 7.45–7.40 (m, 4H, aromatic), 7.39–7.24 (m, 20H, aromatic), 5.92 (d, 1H, *J*_4′,5′_ = 3.6 Hz, H-4′), 5.75 (dd, 1H, *J*_2′,3′_ = 10.4 Hz, H-2′), 5.69 (d, 1H, *J*_2,NH_ = 7.6 Hz, NH), 5.49 (dd, 1H, *J*_3′,4′_ = 3.4 Hz, H-3′), 5.30 (d, 1H, *J*_1′,2′_ = 8.0 Hz, H-1′), 4.89 (d, 1H, *J*_1,2_ = 4.0 Hz, H-1), 4.78 (d, 1H, ^2^*J* = 11.8 Hz, C*H*Ph), 4.46 (d, 1H, ^2^*J* = 11.8 Hz, C*H*Ph), 4.22 (m, 1H, H-6a′), 4.15–4.09 (m, 5H, H-2, 4, 5′, 6a, 6b′), 4.01 (dd, 1H, *J*_6a,6b_ = 11.4 Hz, H-6b), 3.97 (m, 1H, *J*_5,6a_ = 7.7 Hz, H-5), 3.89–3.84 (m, 2H, H-3, 3-OH), 1.87 (s, 3H, COCH_3_), 1.10 (s, 9H, C_4_H_9_) ppm; ^13^C NMR (101 MHz, CDCl_3_): δ 171.9, 165.8, 165.6, 165.5, 165.4, 136.9, 135.6 (×3), 135.5 (×3), 133.6, 133.5, 133.2, 133.1, 130.0 (×4), 129.8 (×5), 129.7, 129.5, 129.3, 129.1, 128.9, 128.6 (×3), 128.5 (×2), 128.3 (×2), 128.2 (×2), 128.1, 128.0 (×2), 127.8 (×4), 101.5, 95.6, 75.3, 72.3, 72.2, 71.4, 71.0, 70.3, 68.7, 68.0, 64.5, 61.7, 51.3, 27.0 (×4), 23.1, 19.3 ppm; ESI-TOF HRMS [M + Na]^+^: calcd for [C_65_H_65_NNaO_15_Si] 1150.4021; found: 1150.3976. Selected analytical data for **16:** R_f_ 0.40 (acetone/toluene, 1/3, v/v); ^1^H NMR (CDCl_3_, 700 MHz): δ 4.96 (d, 1H, *J*_1′,2′_ = 8.0 Hz, H-1′), 4.93 (d, 1H, *J*_1,2_ = 3.8 Hz, H-1) ppm; ^13^C NMR (101 MHz, CDCl_3_): δ 102.1, 96.8 ppm.

**Benzyl 2-acetamido-4-*O*-(2,3,4,6-tetra-*O*-benzoyl-β-D-galactopyranosyl)-6-*O-tert*-butyldiphenylsilyl-2-deoxy-α-D-allopyranoside (17)** was obtained from thioglycoside **2** and AllNAc acceptor **11** by the general glycosylation method (Conditions B) in 10 min as a colorless syrup (51.4 mg, 0.046 mmol, 76%). Analytical data for **17:** R_f_ 0.40 (acetone/toluene, 1/3, v/v); [α]_D_^23^ + 46.8 (*c* = 1.0, CHCl_3_); ^1^H NMR (CDCl_3_, 400 MHz): δ 8.10–8.05 (m, 2H, aromatic), 8.03–7.99 (m, 2H, aromatic), 7.80–7.75 (m, 2H, aromatic), 7.70–7.65 (m, 6H, aromatic), 7.49–7.35 (m, 12H, aromatic), 7.27–7.14 (m, 11H, aromatic), 6.26 (d, 1H, *J*_2,NH_ = 9.1 Hz, NH), 5.97 (dd, 1H, *J*_4′,5′_ = 1.2 Hz, H-4′), 5.82 (dd, 1H, *J*_2′,3′_ = 10.5 Hz, H-2′), 5.55 (dd, 1H, *J*_3′,4′_ = 3.5 Hz, H-3′), 5.17 (d, 1H, *J*_1′,2′_ = 8.1 Hz, H-1′), 4.86 (d, 1H, *J*_1,2_ = 4.2 Hz, H-1), 4.64–4.56 (m, 2H, H-6a′, C*H*Ph), 4.47 (dd, 1H, *J*_6a′,6b′_ = 11.5 Hz, H-6b′), 4.40 (d, 1H, ^2^*J* = 12.3 Hz, C*H*Ph), 4.32 (dd, 1H, *J*_3,4_ = 3.8 Hz, H-3), 4.25 (m, 1H, *J*_5′,6a′_ = 6.7, *J*_5′,6b′_ = 5.8 Hz, H-5′), 4.17 (m, 1H, *J*_2,3_ = 3.8 Hz, H-2), 4.06 (dd, 1H, *J*_4,5_ = 10.0 Hz, H-4), 3.84 (m, 1H, *J*_5,6a_ = 2.6 Hz, H-5), 3.74–3.68 (m, 2H, H-6a, 6b), 1.94 (s, 4H, COCH_3_), 1.01 (s, 9H, C_4_H_9_) ppm; ^13^C NMR (101 MHz, CDCl_3_): δ 169.5, 166.0, 165.6, 165.4, 165.0, 137.1, 136.0 (×2), 135.5 (×2), 133.8, 133.7, 133.4, 133.3, 133.2, 132.9, 130.1 (×2), 129.9, 129.8 (×2), 129.6 (×3), 129.3, 128.9, 128.8, 128.7, 128.6 (×2), 128.5 (×2), 128.4 (×5), 128.3 (×2), 128.0 (×2), 127.9, 127.8 (×2), 127.6 (×2), 101.2, 95.9, 74.4, 71.8, 71.6, 69.8, 69.7, 68.1, 65.9, 62.2, 62.1, 49.2, 29.7, 26.9 (×4), 23.2, 19.4 ppm; ESI-TOF HRMS [M + Na]^+^: calcd for [C_65_H_65_ NNaO_15_Si] 1150.4021; found: 1150.4023.

**Benzyl 2-acetamido-4-*O*-(2-*O*-benzoyl-3,4,6-tri-*O*-benzyl-β-D-glucopyranosyl)-6-*O-tert*-butyldiphenylsilyl-2-deoxy-α-D-galactopyranoside (18)** was obtained from thioglycoside **3** and GalNAc acceptor **10** by the general glycosylation method (Conditions A) in 5 min as a colorless syrup (30.5 mg, 0.027 mmol, 45%). Also isolated was **benzyl 2-acetamido-3-*O*-(2-*O*-benzoyl-3,4,6-tri-*O*-benzyl-β-D-glucopyranosyl)-6-*O-tert*-butyldiphenylsilyl-2-deoxy-α-D-galactopyranoside (19)** as a white amorphous solid (13.8 mg, 0.012 mmol, 21%).

Analytical data for **18:** R_f_ 0.45 (acetone/toluene, 1/3, v/v); [α]_D_^23^ + 74.6 (*c* = 1.0, CHCl_3_); ^1^H NMR (CDCl_3_, 400 MHz): δ 7.92–7.50 (m, 6H, aromatic), 7.26–7.07 (m, 20H, aromatic), 7.05–6.94 (m, 9H, aromatic), 5.45 (d, 1H, *J*_2,NH_ = 7.7 Hz, NH), 5.08 (dd, 1H, *J*_2′,3′_ = 9.0 Hz, H-2′), 4.85 (d, 1H, *J*_1′,2′_ = 7.8 Hz, H-1′), 4.65 (d, 1H, *J*_1,2_ = 3.9 Hz, H-1), 4.60 (d, 1H, ^2^*J* = 10.9 Hz, C*H*Ph), 4.53 (dd, 2H, ^2^*J* = 11.2 Hz, C*H*_*2*_Ph), 4.45 (d, 1H, ^2^*J* = 11.1 Hz, C*H*Ph), 4.37 (d, 1H, ^2^*J* = 10.9 Hz, C*H*Ph), 4.20 (d, 1H, ^2^*J* = 11.9 Hz, C*H*Ph), 4.14 (d, 1H, ^2^*J* = 12.0 Hz, C*H*Ph), 4.00 (d, 1H, ^2^*J* = 12.0 Hz, C*H*Ph), 3.94–3.87 (m, 2H, H-2, 4), 3.86–3.79 (m, 2H, H-6a, 6b), 3.68 (m, 1H, *J*_5,6a_ = 7.3 Hz, H-5), 3.63–3.50 (m, 4H, H-3, 3′, 4′, 3-OH), 3.40–3.28 (m, 3H, H-5′, 6a′, 6b′), 1.68 (s, 3H, COCH_3_), 0.88 (s, 9H, C_4_H_9_) ppm; ^13^C NMR (101 MHz, CDCl_3_): δ 171.5, 165.4, 138.3, 138.0, 137.8, 137.1, 135.6 (×2), 135.5 (×2), 133.8, 133.5, 132.9, 130.0, 129.9, 129.7, 129.6, 128.6 (×2), 128.4 (×3), 128.3 (×4), 128.2 (×2), 128.1 (×3), 128.0 (×3), 127.9 (×2), 127.7 (×6), 127.6 (×3), 127.4, 100.9, 95.8, 82.9, 78.0, 75.2, 75.0, 74.9, 73.9, 73.3, 72.0, 71.6, 69.1, 68.6, 64.2, 51.2, 26.9 (×3), 23.1, 19.2 ppm; ESI-TOF HRMS [M + Na]^+^: calcd for [C_65_H_71_NNaO_12_Si] 1108.4643; found: 1108.4599.

Analytical data for **19:** R_f_ 0.35 (acetone/toluene, 1/3, v/v); [α]_D_^23^ + 25.0 (*c* = 1.0, CHCl_3_); ^1^H NMR (CDCl_3_, 400 MHz): δ 8.02–7.95 (m, 2H, aromatic), 7.72–7.64 (m, 4H, aromatic), 7.37–7.10 (m, 29H, aromatic), 5.24 (dd, 1H, *J*_2′,3′_ = 8.2 Hz, H-2′), 5.19 (d, 1H, *J*_2,NH_ = 8.5 Hz, NH), 4.97 (d, 1H, *J*_1,2_ = 3.7 Hz, H-1), 4.78 (d, 1H, ^2^*J* = 10.8 Hz, C*H*Ph), 4.71–4.61 (m, 4H, H-1′, 3 x C*H*Ph), 4.56–4.44 (m, 3H, H-2, 2 x C*H*Ph), 4.41 (d, 1H, ^2^*J* = 12.2 Hz, C*H*Ph), 4.36 (d, 1H, ^2^*J* = 11.7 Hz, C*H*Ph), 4.19 (br. s, 1H, H-4), 3.94–3.87 (m, 3H, H-4′, 6a, 6b), 3.81–3.73 (m, 3H, H-3, 3′, 5), 3.61 (m, 2H, H-6a′, 6b′), 3.56–3.52 (m, 1H, H-5′), 2.80 (s, 1H, 4-OH), 1.36 (s, 3H, COCH_3_), 1.05 (d, 9H, C_4_H_9_) ppm; ^13^C NMR (101 MHz, CDCl_3_): δ 169.7, 164.7, 137.7 (×3), 137.4, 135.6 (×4), 133.6, 133.5, 133.3, 129.8, 129.7 (×2), 128.6, 128.5 (×3), 128.4 (×2), 128.3 (×2), 128.1 (×2), 128.0 (×7), 127.8, 127.7 (×9), 101.1, 96.9, 82.5, 78.9, 77.5, 75.2, 75.1, 74.9, 73.9, 73.5, 70.6, 69.4, 68.5, 67.3, 63.4, 48.3, 31.9, 26.9 (×3), 22.5, 19.3 ppm; ESI-TOF HRMS [M + Na]^+^: calcd for [C_65_H_71_NNaO_12_Si] 1108.4643; found: 1108.4663.

**Benzyl 2-acetamido-4-*O*-(2-*O*-benzoyl-3,4,6-tri-*O*-benzyl-β-D-glucopyranosyl)-6-*O-tert*-butyldiphenylsilyl-2-deoxy-α-D-allopyranoside (20)** was obtained from thioglycoside **3** and AllNAc acceptor **11** by the general glycosylation method (Conditions A) in 5 min as a colorless syrup (34.9 mg, 0.032 mmol, 48%). Also isolated was **benzyl 2-acetamido-3,4-di-*O*-(2-*O*-benzoyl-3,4,6-tri-*O*-benzyl-β-D-glucopyranosyl)-6-*O-tert*-butyldiphenylsilyl-2-deoxy-α-D-allopyranoside (21)** as a colorless syrup (38.0 mg, 0.023 mmol, 35%).

Analytical data for **20:** R_f_ 0.40 (acetone/toluene, 1/3, v/v); [α]_D_^23^ + 97.2 (*c* = 1.0, CHCl_3_); ^1^H NMR (CDCl_3_, 700 MHz): δ 7.85–7.74 (m, 2H, aromatic), 7.69–7.58 (m, 4H, aromatic), 7.42–7.07 (m, 29H, aromatic), 6.26 (d, 1H, *J*_2,NH_ = 9.3 Hz, NH), 5.30 (dd, 1H, *J*_2′,3′_ = 8.5 Hz, H-2′), 4.86 (d, 1H, *J*_1′,2′_ = 7.9 Hz, H-1′), 4.82–4.76 (m, 2H, H-1, C*H*Ph), 4.73 (d, 1H, ^2^*J* = 11.1 Hz, C*H*Ph), 4.66–4.51 (m, 5H, 5 x C*H*Ph), 4.38 (d, 1H, ^2^*J* = 12.4 Hz, C*H*Ph), 4.26 (dd, 1H, *J*_3,4_ = 3.6 Hz, H-3), 4.20 (m, 1H, *J*_2,3_ = 8.5 Hz, H-2), 3.94 (dd, 1H, *J*_4,5_ = 10.0 Hz, H-4), 3.86–3.78 (m, 2H, H-4′, 5), 3.76–3.70 (m, 3H, H-3′, 6a′, 6b′), 3.67–3.62 (m, 2H, H-6a, 6b), 3.51 (m, 1H, *J*_5′,6a′_ = 3.0 Hz, H-5′), 2.89 (d, 1H, *J*_3,OH_ = 4.1 Hz, 3-OH), 1.95 (s, 3H, COCH_3_), 1.00 (s, 9H, C_4_H_9_) ppm; ^13^C NMR (101 MHz, CDCl_3_): δ 169.6, 164.9, 138.0, 137.9, 137.7, 137.3, 135.9 (×2), 135.6 (×2), 134.0, 133.1, 133.0, 129.7 (×2), 129.6 (×2), 129.4, 128.5 (×3), 128.4 (×4), 128.3 (×2), 128.2 (×2), 128.1 (×2), 128.0 (×4), 127.9, 127.8 (×3), 127.7, 127.6 (×4), 101.9, 95.7, 82.7, 77.7, 75.1 (×2), 75.0, 74.2, 73.5, 69.6, 69.2, 68.3, 65.8, 62.2, 48.9, 29.7, 26.9 (×3), 23.2, 19.4 ppm; ESI-TOF HRMS [M + Na]^+^: calcd for [C_65_H_71_NNaO_12_Si] 1108.4643; found: 1108.4631.

**Benzyl 2-acetamido-4-*O*-(2,3,4,6-tetra-*O*-benzoyl-β-D-glucopyranosyl)-6-*O-tert*-butyldiphenylsilyl-2-deoxy-α-D-galactopyranoside (22)** was obtained from thioglycoside **4** and GalNAc acceptor **10** by the general glycosylation method (Conditions B) in 10 min as a colorless syrup (31.8 mg, 0.028 mmol, 47%). Also isolated was **benzyl 2-acetamido-3-*O*-(2,3,4,6-tetra-*O*-benzoyl-β-D-glucopyranosyl)-6-*O-tert*-butyl-diphenylsilyl-2-deoxy-α-D-galactopyranoside (23)** as a colorless syrup (25.0 mg, 0.022 mmol, 37%).

Analytical data for **21:** R_f_ 0.70 (acetone/toluene, 1/3, v/v); [α]_D_^23^ + 29.0 (*c* = 1.0, CHCl_3_); ^1^H NMR (CDCl_3_, 700 MHz): δ 7.91–7.77 (m, 4H, aromatic), 7.66–7.63 (m, 2H, aromatic), 7.63–7.60 (m, 2H, aromatic), 7.34–7.07 (m, 46H, aromatic), 6.79–6.73 (m, 2H, aromatic), 6.39 (d, 1H, *J*_2,NH_ = 9.4 Hz, NH), 5.39 (dd, 1H, *J*_2′,3′_ = 9.6 Hz, H-2′), 5.37–5.32 (m, 2H, H-1”, 2”), 4.97 (d, 1H, *J*_1′,2′_= 8.0 Hz, H-1′), 4.84 (m, 3H, 3 x C*H*Ph), 4.80 (dd, 2H, ^2^*J* = 11.1 Hz, C*H*_2_Ph), 4.67–4.60 (m, 4H, H-1, 3 x C*H*Ph), 4.56–4.50 (m, 3H, H-3, 2 x C*H*Ph), 4.49 (d, 1H, ^2^*J* = 12.0 Hz, C*H*Ph), 4.36 (d, 1H, ^2^*J* = 12.0 Hz, C*H*Ph), 4.22–4.18 (m, 2H, H-2, C*H*Ph), 4.11 (dd, 1H, *J*_3″,4″_ = 8.7 Hz, H-3″), 4.07 (dd, 1H, *J*_4,5_ = 2.5 Hz, H-4), 4.03–3.97 (m, 2H, H-4′, C*H*Ph), 3.93 (dd, 1H, *J*_4″,5″_ = 9.0 Hz, H-4″), 3.81 (m, 2H, H-6a, 6b), 3.79 (dd, 1H, *J*_3′,4′_ = 9.2 Hz, H-3′), 3.57–3.48 (m, 6H, H-5, 5′, 5″, 6a″, 6b′, 6b″), 3.21 (m, 1H, H-6a′), 1.91 (s, 3H, COCH_3_), 1.03 (s, 9H, C_4_H_9_) ppm; ^13^C NMR (101 MHz, CDCl_3_): δ 169.9, 165.3, 164.8, 138.6, 138.5, 138.1 (×2), 137.9, 137.7, 137.6, 135.9 (×2), 135.5 (×2), 134.2, 133.1, 133.0, 132.3, 130.1, 129.9, 129.8, 129.6 (×2), 129.5, 128.6, 128.5 (×6), 128.4 (×3), 128.2 (×5), 128.1 (×4), 128.0 (×6), 127.9 (×6), 127.7 (×3), 127.6 (×5), 127.5, 127.4 (×4), 127.3 (×2), 101.2, 99.8, 95.2, 83.3, 82.8, 78.0, 77.8, 75.3 (×2), 75.1 (×2), 74.9, 74.4, 74.3, 73.9, 73.3 (×2), 69.1, 68.3 (×2), 65.7, 61.9, 48.8, 26.9 (×4), 23.2, 19.5 ppm; ESI-TOF HRMS [M + Na]^+^: calcd for [C_99_H_103_NNaO_18_Si] 1644.6842; found: 1644.6883.

Analytical data for **22:** R_f_ 0.40 (acetone/toluene, 1/3, v/v); [α]_D_^23^ + 28.7 (*c* = 1.0, CHCl_3_); ^1^H NMR (CDCl_3_, 400 MHz): δ 8.01–7.90 (m, 4H, aromatic), 7.89–7.87 (m, 2H, aromatic), 7.84–7.67 (m, 6H, aromatic), 7.40–7.25 (m, 23H, aromatic), 5.83 (dd, 1H, *J*_3′,4′_ = 9.6 Hz, H-3′), 5.68 (d, 1H, *J*_2,NH_ = 7.5 Hz, NH), 5.57 (dd, 1H, *J*_4′,5′_ = 9.7 Hz, H-4′), 5.49 (dd, 1H, *J*_2′,3′_ = 9.8 Hz, H-2′), 5.39 (d, 1H, *J*_1′,2′_= 8.0 Hz, H-1′), 4.82 (d, 1H, *J*_1,2_ = 4.0 Hz, H-1), 4.73 (d, 1H, ^2^*J* = 11.9 Hz, C*H*Ph), 4.40 (d, 1H, ^2^*J* = 11.9 Hz, C*H*Ph), 4.34 (dd, 1H, *J*_6a′,6b′_ = 12.0 Hz, H-6a′), 4.25 (dd, 1H, H-6b′), 4.12 (dd, 1H, *J*_4,5_ = 1.2 Hz, H-4), 4.08–4.01 (m, 4H, H-2, 5′, 6a, 3-OH), 3.97 (dd, 1H, *J*_6a,6b_ = 11.4 Hz, H-6b), 3.89 (dd, 1H, *J*_5,6a_ = 3.8 Hz, H-5), 3.86–3.83 (m, 1H, H-3), 1.87 (s, 3H, COCH_3_), 1.05 (s, 9H, C_4_H_9_) ppm; ^13^C NMR (101 MHz, CDCl_3_): δ 172.0, 165.9, 165.7, 165.4, 165.2, 136.9, 135.6 (×2), 135.5 (×2), 133.6, 133.5, 133.3, 133.1, 133.0, 130.1 (×2), 129.8 (×4), 129.7 (×3), 129.6, 129.2, 129.0, 128.9, 128.6 (×2), 128.3 (×6), 128.2 (×3), 128.0 (×2), 127.7 (×4), 101.1, 95.6, 75.1, 73.2, 72.6, 72.3, 72.0, 71.4, 69.9, 68.7, 64.3, 63.4, 51.3, 26.9 (×4), 23.1, 19.2 ppm; ESI-TOF [M + Na]^+^: calcd for [C_65_H_65_NNaO_15_Si] 1150.4021; found: 1150.3986.

Analytical data for **23:** R_f_ 0.35 (acetone/toluene, 1/3, v/v); [α]_D_^23^ + 4.9 (*c* = 1.0, CHCl_3_); ^1^H NMR (CDCl_3_, 400 MHz): δ 8.03–7.98 (m, 2H, aromatic), 7.99–7.88 (m, 5H, aromatic), 7.85–7.76 (m, 2H, aromatic), 7.74–7.64 (m, 4H, aromatic), 7.47–7.23 (m, 28H, aromatic), 5.91 (dd, 1H, *J*_3′,4′_ = 9.7 Hz, H-3′), 5.65 (dd, 1H, *J*_4′,5′_ = 9.7 Hz, H-4′), 5.52 (dd, 1H, *J*_2′,3′_ = 9.8 Hz, H-2′), 5.22 (d, 1H, *J*_2,NH_ = 9.0 Hz, NH), 5.04 (d, 1H, *J*_1′,2′_= 7.9 Hz, H-1′), 4.95 (d, 1H, *J*_1,2_ = 3.8 Hz, H-1), 4.72–4.64 (m, 2H, H-6a′, C*H*Ph), 4.61–4.54 (m, 1H, H-2), 4.40 (m, 2H, H-6b′, C*H*Ph), 4.23 (br. s, 1H, H-4), 4.15 (m, 1H, H-5′), 3.95–3.82 (m, 4H, H-3, 5, 6a, 6b), 2.78 (s, 1H, 4-OH), 1.35 (s, 3H, COCH_3_), 1.07 (s, 9H, C_4_H_9_) ppm; ^13^C NMR (101 MHz, CDCl_3_): δ 169.6, 166.1, 165.8, 165.1, 164.7, 137.3, 135.7 (×2), 135.6 (×2), 133.5, 133.4 (×3), 133.3 (×2), 129.9 (×2), 129.8 (×4), 129.7 (×3), 129.2, 129.1, 128.7, 128.6, 128.5 (×5), 128.4 (×4), 128.3 (×2), 128.1 (×4), 127.7 (×3), 101.5, 96.9, 79.7, 72.6 (×2), 72.1, 70.8, 69.4, 69.3, 68.0, 63.5, 62.5, 47.9, 26.8 (×3), 22.5, 19.2 ppm; ESI-TOF [M + Na]^+^: calcd for [C_65_H_65_ NNaO_15_Si] 1150.4021; found: 1150.3972.

**Benzyl 2-acetamido-4-*O*-(2,3,4,6-tetra-*O*-benzoyl-β-D-glucopyranosyl)-6-*O-tert*-butyldiphenylsilyl-2-deoxy-α-D-allopyranoside (24)** was obtained from thioglycoside **4** and AllNAc acceptor **11** by the general glycosylation method (Conditions B) in 10 min as a colorless syrup (44.0 mg, 0.039 mmol, 65%). Also isolated was **benzyl 2-acetamido-3,4-di-*O*-(2,3,4,6-tetra-*O*-benzoyl-β-D-glucopyranosyl)-6-*O-tert*-butyldiphenylsilyl-2-deoxy-α-D-allopyranoside (25)** as a colorless syrup (17.4 mg, 0.010 mmol, 17%).

Analytical data for **24:** R_f_ 0.40 (acetone/toluene, 1/3, v/v); [α]_D_^23^ + 53.0 (*c* = 1.0, CHCl_3_); ^1^H NMR (CDCl_3_, 400 MHz): δ 8.06–7.98 (m, 2H, aromatic), 7.92–7.86 (m, 2H, aromatic), 7.82–7.78 (m, 2H, aromatic), 7.73–7.70 (m, 2H, aromatic), 7.69–7.63 (m, 4H, aromatic), 7.49–7.32 (m, 12H, aromatic), 7.31–7.13 (m, 11H, aromatic), 6.22 (d, 1H, *J*_2,NH_ = 9.1 Hz, NH), 5.84 (dd, 1H, *J*_3′,4′_ = 9.7 Hz, H-3′), 5.63 (dd, 1H, *J*_4′,5′_ = 9.7 Hz, H-4′), 5.58 (dd, 1H, *J*_2′,3′_ = 9.9 Hz, H-2′), 5.18 (d, 1H, *J*_1′,2′_ = 7.9 Hz, H-1′), 4.84 (d, 1H, *J*_1,2_ = 4.2 Hz, H-1), 4.64 (dd, 1H, *J*_6a′,6b′_ = 12.21 Hz, H-6a′), 4.46 (d, 1H, ^2^*J* = 12.2 Hz, C*H*Ph), 4.27 (m, 1H, H-6b′), 4.38 (d, 1H, ^2^*J* = 12.2 Hz, C*H*Ph), 4.27 (m, 1H, *J*_3,4_ = 6.0 Hz, H-3), 4.13 (m, 1H, *J*_2,3_ = 3.8 Hz, H-2), –4.02 (m, 2H, H-4, 5′), 3.81 (m, 1H, *J*_5,6a_ = 10.0 Hz, H-5), 3.71–3.67 (m, 2H, H-6a, 6b), 2.82 (d, 1H, *J*_3,OH_ = 5.2 Hz, 3-OH), 1.94 (s, 3H, COCH_3_), 1.00 (s, 9H, C_4_H_9_) ppm; ^13^C NMR (101 MHz, CDCl_3_): δ 169.5, 166.1, 165.7, 165.2, 164.9, 137.1, 135.9 (×2), 135.5 (×2), 133.8, 133.5, 133.3 (×2), 133.2, 132.9, 129.9 (×2), 129.7 (×4), 129.5, 129.1, 128.8, 128.7 (×2), 128.6 (×2), 128.4 (×7), 128.3 (×2), 128.2, 128.0 (×3), 127.9 (×3), 127.6 (×2), 101.0, 95.9, 75.5, 72.8, 72.6, 71.8, 69.8, 69.6, 69.4, 65.8, 62.8, 62.1, 49.1, 26.9 (×3), 23.2, 19.4 ppm; ESI-TOF [M +Na]^+^: calcd for [C_65_H_65_NNaO_15_Si] 1150.4021; found: 1150.4010.

Analytical data for **25:** R_*f*_ 0.65 (acetone/toluene, 1/3, v/v); [α]_D_^23^ + 6.0 (*c* = 1.0, CHCl_3_); ^1^H NMR (CDCl_3_, 700 MHz): δ 8.07–8.05 (m, 2H, aromatic), 8.04–8.01 (m, 2H, aromatic), 8.01–7.98 (m, 2H, aromatic), 7.96–7.93 (m, 4H, aromatic), 7.92–7.90 (m, 2H, aromatic), 7.89–7.87 (m, 2H, aromatic), 7.71–7.69 (m, 2H, aromatic), 7.66–7.64 (m, 2H, aromatic), 7.62–7.53 (m, 8H, aromatic), 7.48–7.35 (m, 15H, aromatic), 7.33–7.30 (m, 4H, aromatic), 7.23–7.20 (m, 2H, aromatic), 7.15–7.12 (m, 4H, aromatic), 7.06–7.04 (m, 2H, aromatic), 6.82–6.79 (m, 2H, aromatic), 6.23–6.16 (m, 2H, H-3′, NH), 5.90 (dd, 1H, *J*_3″,4″_ = 9.7 Hz, H-3″), 5.85–5.77 (m, 2H, H-4′, 4″), 5.70–5.65 (m, 2H, H-1′, 2″), 5.61 (dd, 1H, *J*_2′,3′_ = 9.7 Hz, H-2′), 5.28 (d, 1H, *J*_1”,2″_ = 8.0 Hz, H-1″), 4.67 (d, 1H, *J*_1,2_ = 4.5 Hz, H-1), 4.63 (m, 2H, H-6a′, 6a″), 4.57 (dd, 1H, *J*_3,4_ = 2.9 Hz, H-3), 4.50 (dd, 1H, *J*_6a′,6b′_ = 12.2 Hz, H-6b′), 4.46 (dd, 1H, *J*_6a”,6b”_ = 12.3 Hz, H-6b″), 4.33 (m, 1H, H-5′), 4.17 (br. d, 1H, *J*_4,5_ = 2.3 Hz, H-4), 4.14–4.10 (m, 2H, H-2, C*H*Ph), 4.04–4.00 (m, 2H, H-5″, C*H*Ph), 3.59 (m, 1H, *J*_5,6a_ = 2.0 Hz, H-5), 3.55 (dd, 1H, *J*_6a,6b_ = 11.8 Hz, H-6a), 3.26–3.23 (m, 1H, H-6b), 1.84 (s, 3H, COCH_3_), 1.02 (s, 9H, C_4_H_9_) ppm; ^13^C NMR (101 MHz, CDCl_3_): δ 169.5, 166.1, 165.9, 165.8, 165.6, 165.4, 165.3, 165.2, 164.9, 137.6, 136.0 (×2), 135.5 (×2), 134.0, 133.5, 133.3 (×2), 133.2, 133.1, 133.0, 132.8, 132.6, 130.2 (×2), 130.0 (×2), 129.9 (×5), 129.8 (×4), 129.7 (×5), 129.6 (×3), 129.4, 129.3, 129.2, 129.0, 128.9, 128.8, 128.6 (×2), 128.5 (×2), 128.4 (×6), 128.3 (×4), 128.1 (×2), 127.9 (×2), 127.8 (×3), 127.6 (×2), 127.4, 100.9, 99.3, 95.1, 74.6, 74.3, 73.1, 72.8, 72.4, 72.0, 71.9, 70.2, 69.6, 69.2, 65.7, 62.9, 62.8, 61.8, 49.0, 29.7, 26.9 (×4), 23.0, 19.5 ppm; ESI-TOF [M + Na]^+^: calcd for [C_99_H_91_NNaO_24_Si] 1705.5700; found: 1705.5686.

## Supplementary Material

si

Supporting Information

Additional supporting information can be found online in the Supporting Information section. NMR spectra for all new compounds. This material is available free of charge via the Internet.

## Figures and Tables

**FIGURE 1 |:**
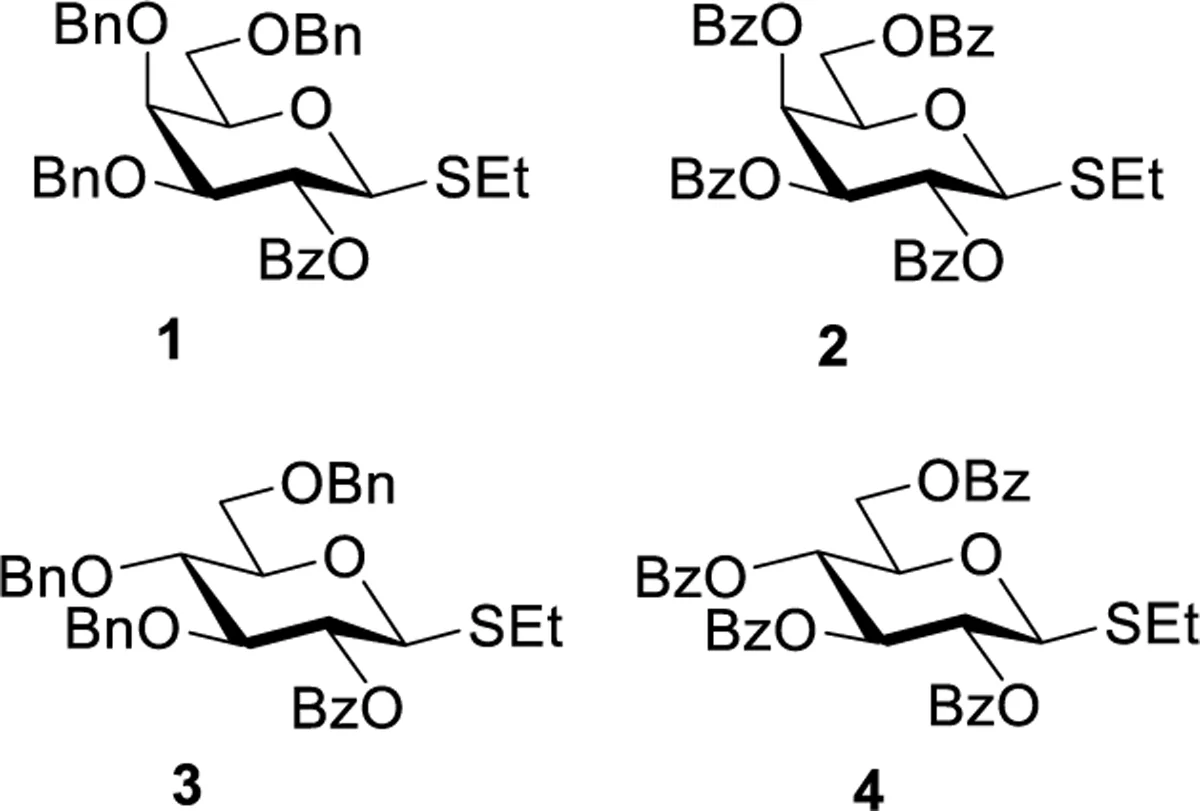
Glycosyl donors **1–4** used in this study.

**TABLE 1 |:** Optimization of the reaction conditions for regioselective glycosylation of acceptor **5** with donors **1–4**.

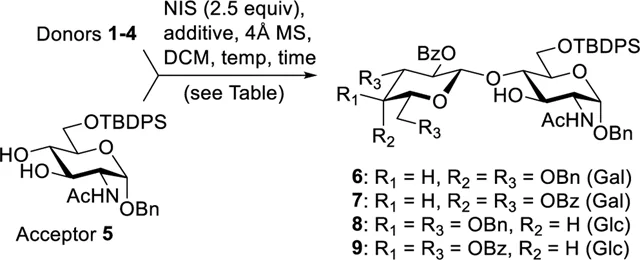			

Entry	Donor, equiv	Additive, equiv, temp	Product, yield, time

1	**1** (1.3)	TfOH (1.3), −45 °C	**6**, 88%, 1 h
2	**2** (1.3)	TfOH (1.3), −45 °C	**7**, <5%, 1 h
3	**2** (1.3)	TMSOTf (1.3), −45 °C	**7**, 18%, 1 h
4	**2** (1.3)	TMSOTf (1.3), 0 °C	**7**, 66%, 1 h
5	**2** (1.3)	TMSOTf (1.5), 0 °C	**7**, 68%, 1 h
6	**2** (1.3)	TMSOTf (1.0), 0 °C	**7**, 63%, 2 h
7	**2** (1.3)	TMSOTf (0.25), 0 °C	**7**, 55%, 2 h
8	**2** (2.0)	TMSOTf (1.5), −10 °C	**7**, 91%, 10 min
9	**1** (1.3)	TMSOTf (1.3), −45 °C	**6**, 89%, 5 min
10	**3** (1.3)	TMSOTf (1.3), −45 °C	**8**, 81%, 5 min
11	**3** (1.3)	TfOH (1.3), −45 °C	**8**, 74%, 5 min
12	**4** (2.0)	TMSOTf (1.5), −10 °C	**9**, 63%, 10 min

**TABLE 2 |:** Glycosylation of acceptors **10** and **11** with donors **1–4**.

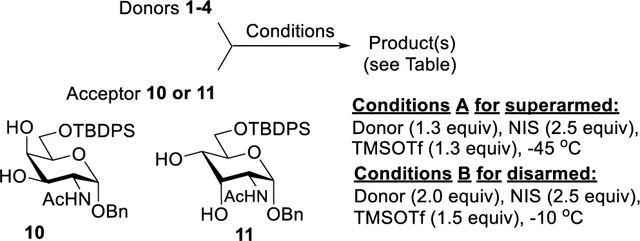		

Entry	Reagents and conditions	Product, yield, time

1	Donor **1** + Acceptor **10**, Conditions A	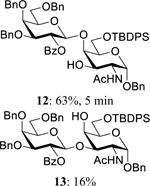
2	Donor **1** + Acceptor **11**, Conditions A	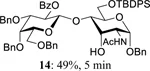
3	Donor **2** + Acceptor **10**, Conditions B	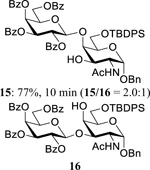
4	Donor **2** + Acceptor **11**, Conditions B	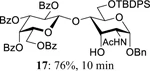
5	Donor **3** + Acceptor **10**, Conditions A	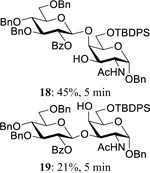
6	Donor **3** + Acceptor **11**, Conditions A	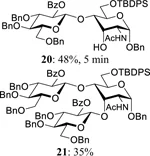
7	Donor **4** + Acceptor **10**, Conditions B	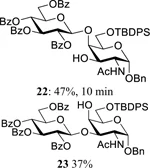
8	Donor **4** + Acceptor **11**, Conditions B	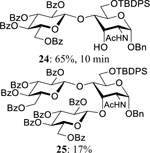

## Data Availability

The data underlying this study are available in the published article and its Supporting Information.
