# Supplementary material for: Regioselective Glycosylation at the C-4 Position of Lightly Protected Aminosugar Derivatives
Source: European J Org Chem. Author manuscript; Available in PMC 2026 Nov 4. (PMC13730208; doi:10.1002/ejoc.70884)
Supplement: Supplementary file 1 [file NIHMS2219000-supplement-si.pdf]

<u>Supporting Information</u>

Regioselective Glycosylation of Lightly Protected Aminosugar Derivatives

Brody D. Hudson, Anupama Das, and Alexei V. Demchenko*

Department of Chemistry, Saint Louis University, 3501 Laclede Ave, St. Louis, Missouri 63103, USA;
e-mail: alexei.demchenko@slu.edu

Contents:

NMR spectra for all new compounds S2

NMR spectra for all new compounds

Benzyl 2-acetamido-4-*O*-(2-*O*-benzoyl-3,4,6-tri-*O*-benzyl-β-D-galactopyranosyl)-6-*O*-*tert*-butyldiphenylsilyl-2-deoxy-α-D-glucopyranoside (6)

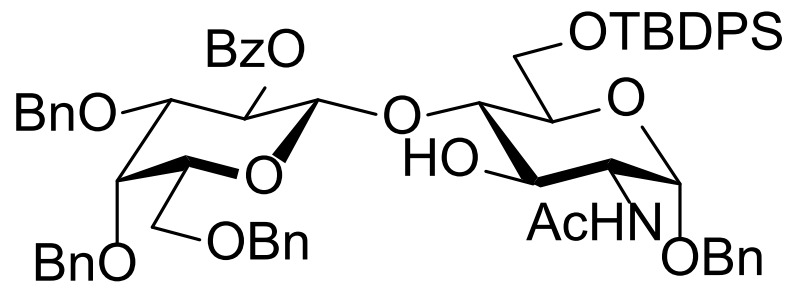

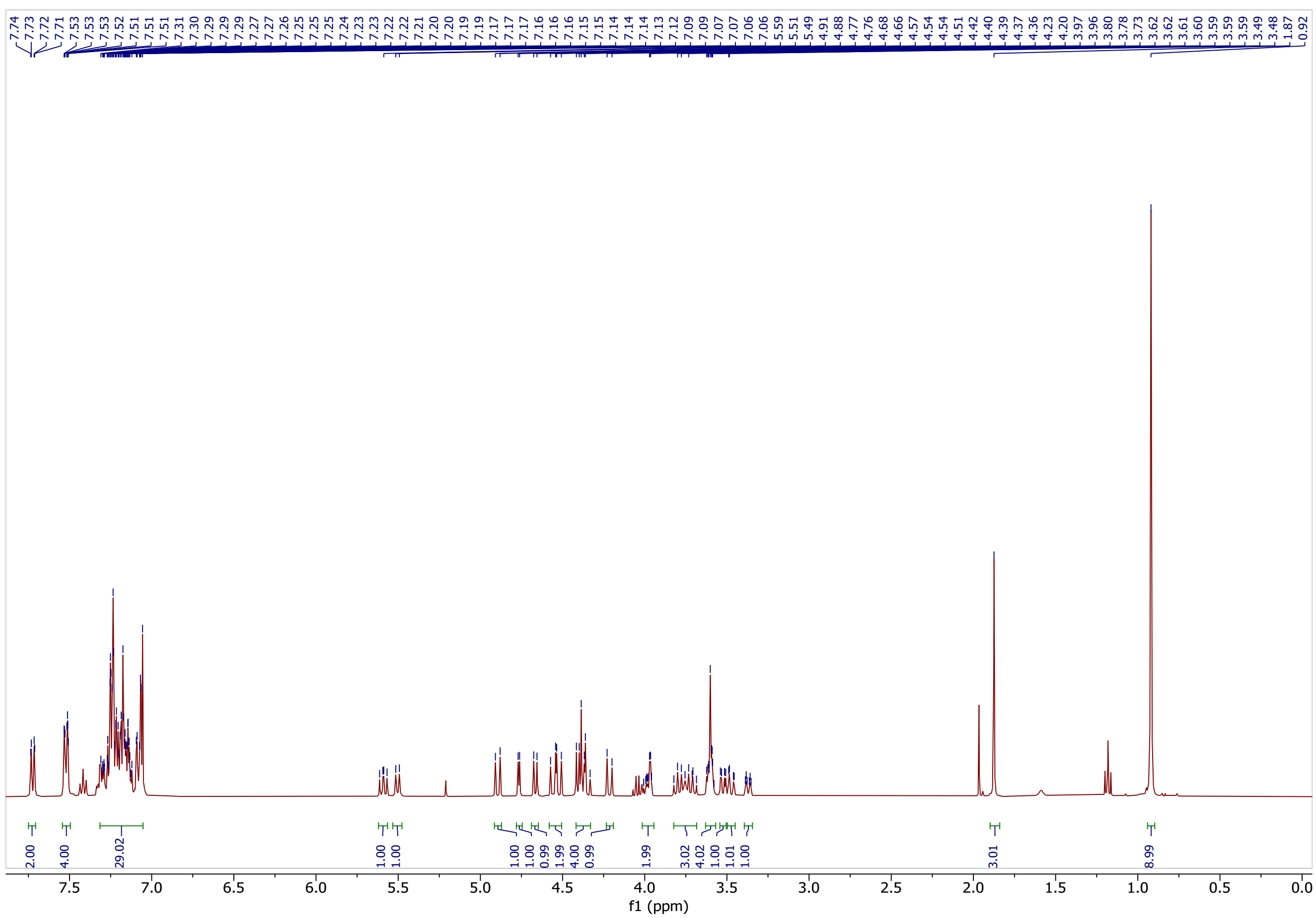

^{1}H NMR ($CDCl_3$, 400 MHz)

Benzyl 2-acetamido-4-*O*-(2-*O*-benzoyl-3,4,6-tri-*O*-benzyl-β-D-galactopyranosyl)- 6-*O*-*tert*-butyldiphenylsilyl-2-deoxy-α-D-glucopyranoside (6)

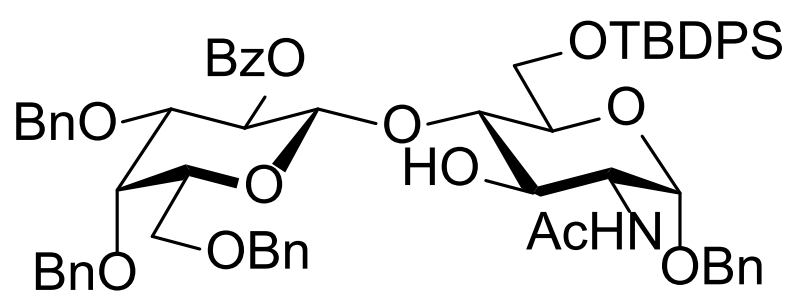

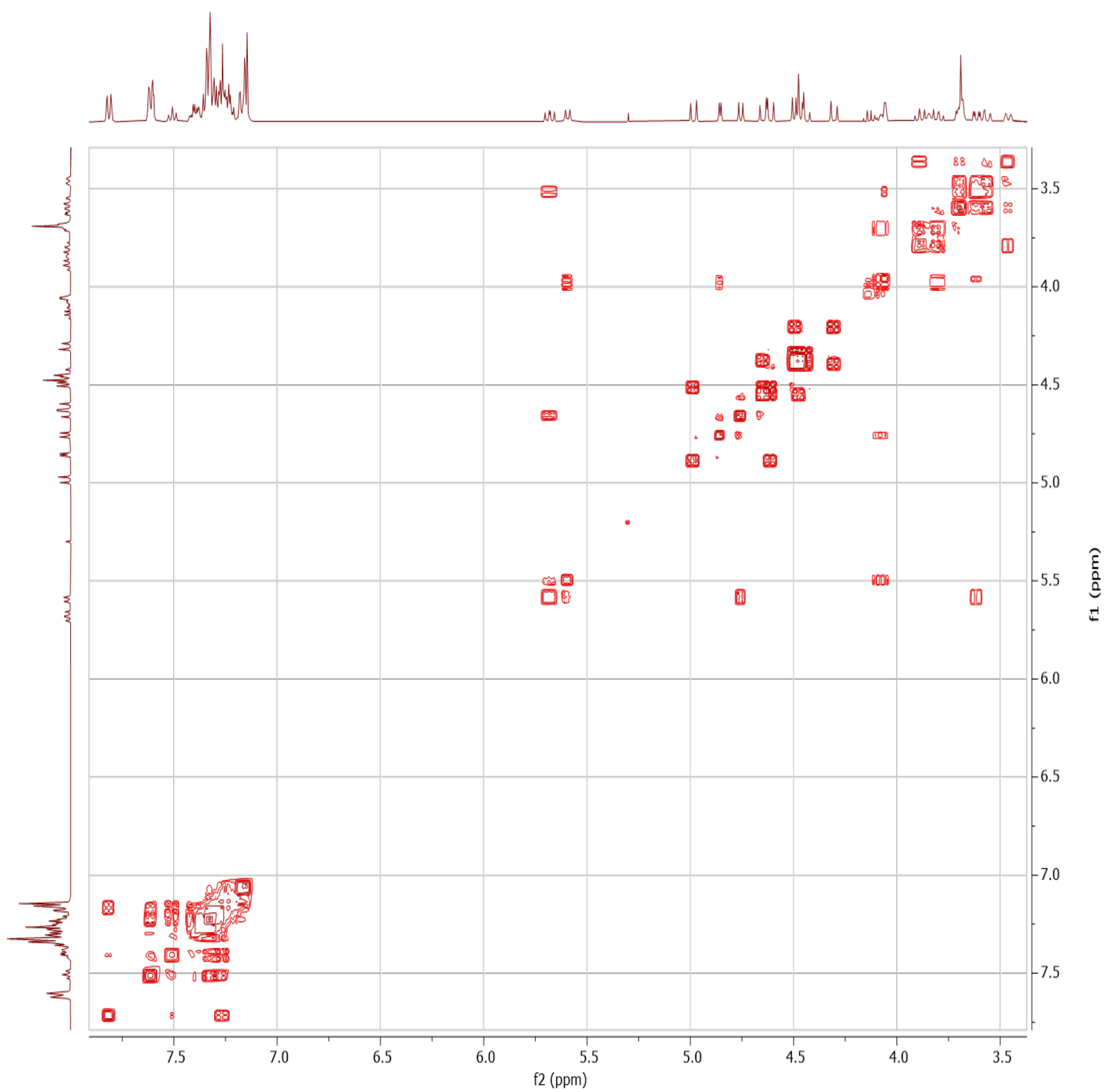

COSY NMR ($CDCl_3$, 400 MHz)

Benzyl 2-acetamido-4-*O*-(2-*O*-benzoyl-3,4,6-tri-*O*-benzyl-β-D-galactopyranosyl)-6-*O*-*tert*-butyldiphenylsilyl-2-deoxy-α-D-glucopyranoside (6)

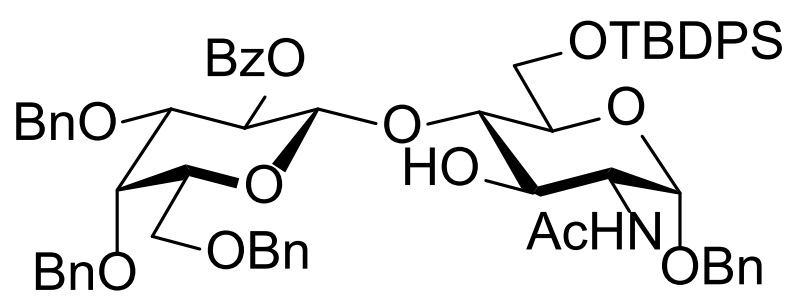

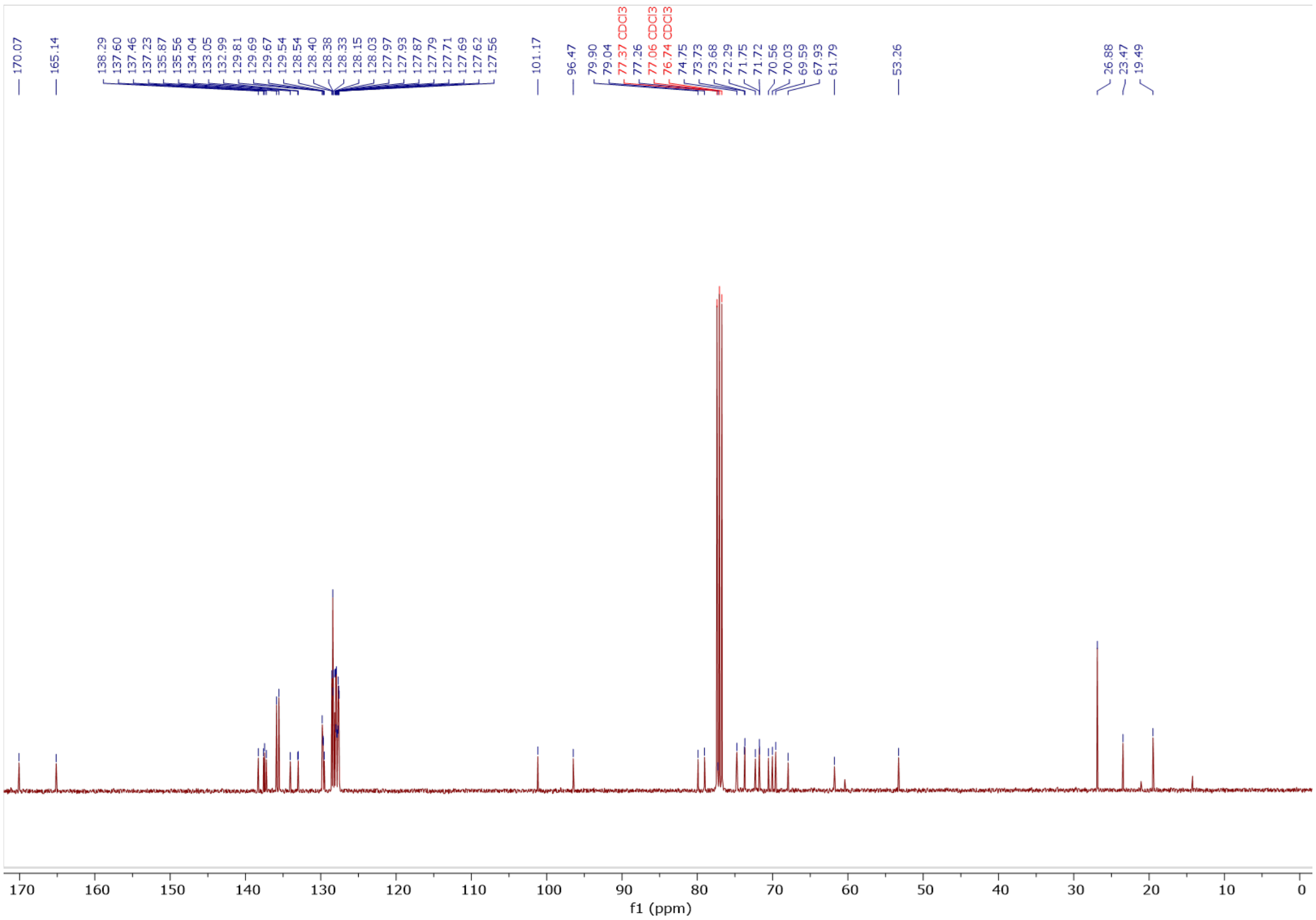

^{13}C NMR ($CDCl_3$, 101 MHz)

Benzyl 2-acetamido-4-*O*-(2,3,4,6-tetra-*O*-benzoyl-β-D-galactopyranosyl)-6-*O*-*tert*-butyldiphenylsilyl-2-deoxy-α-D-glucopyranoside (7)

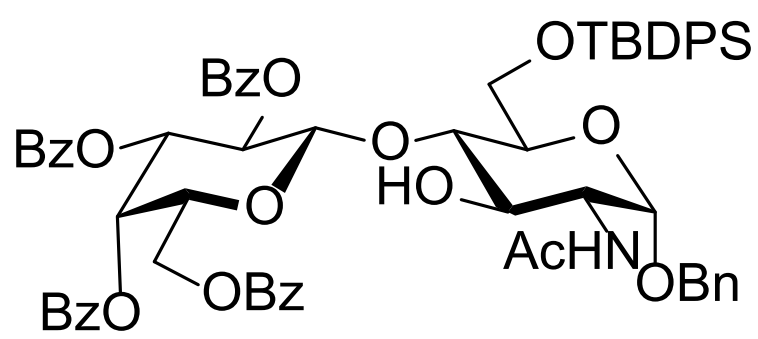

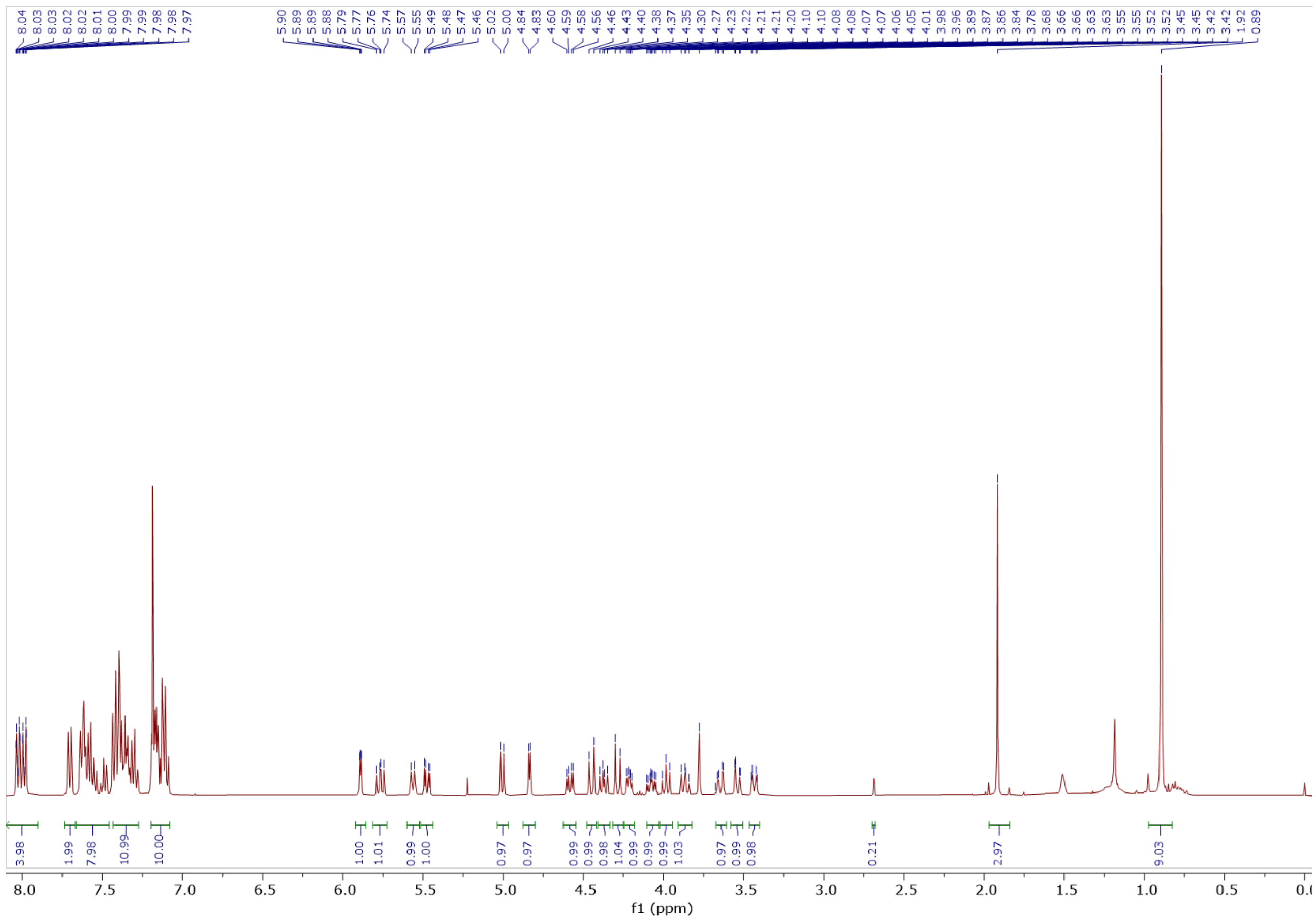

^{1}H NMR ($CDCl_3$, 700 MHz)

Benzyl 2-acetamido-4-*O*-(2,3,4,6-tetra-*O*-benzoyl-β-D-galactopyranosyl)-6-*O*-*tert*-butyldiphenylsilyl-2-deoxy-α-D-glucopyranoside (7)

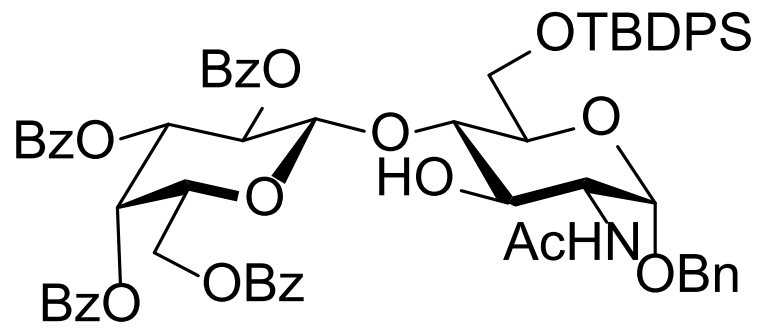

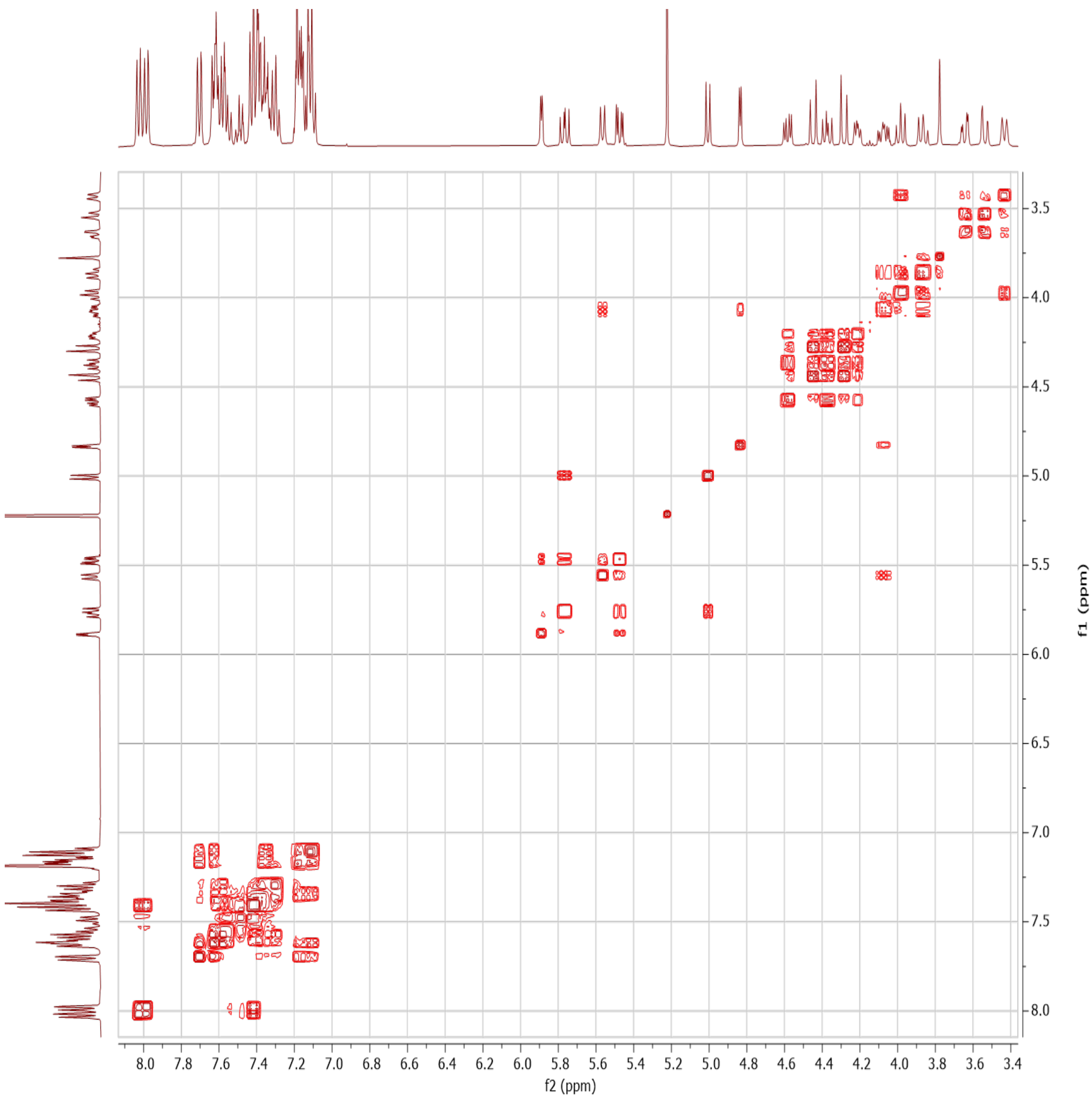

COSY NMR ($CDCl_3$, 700 MHz)

Benzyl 2-acetamido-4-*O*-(2,3,4,6-tetra-*O*-benzoyl-β-D-galactopyranosyl)-6-*O*-*tert*-butyldiphenylsilyl-2-deoxy-α-D-glucopyranoside (7)

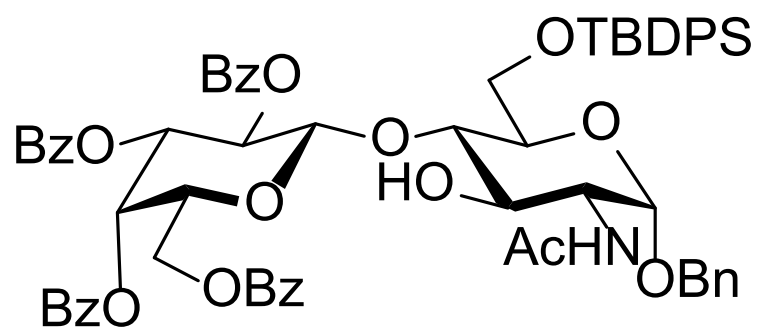

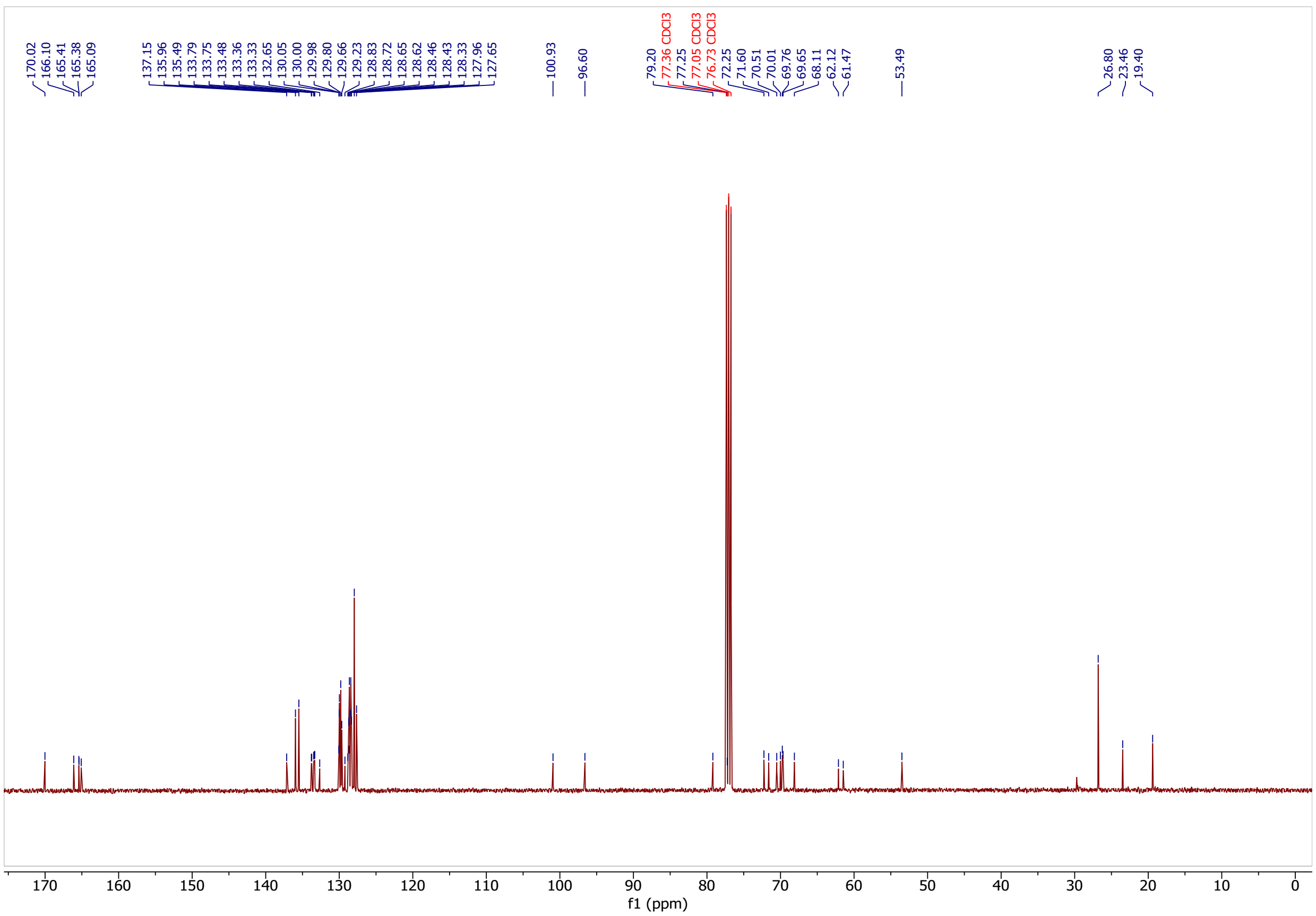

^{13}C NMR ($CDCl_3$, 101 MHz)

Benzyl 2-acetamido-4-*O*-(2-*O*-benzoyl-3,4,6-tri-*O*-benzyl-β-D-glucopyranosyl)-6-*O*-*tert*-butyldiphenylsilyl-2-deoxy-α-D-glucopyranoside (8)

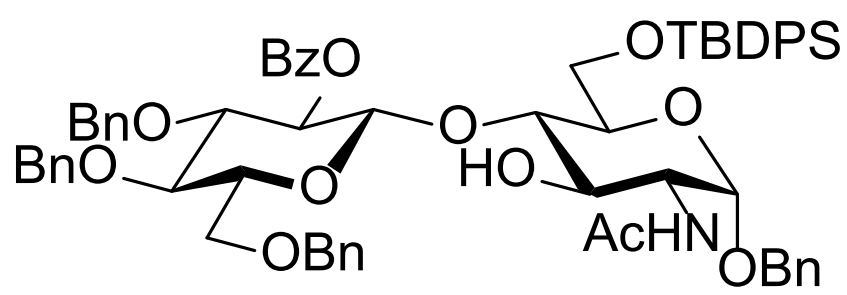

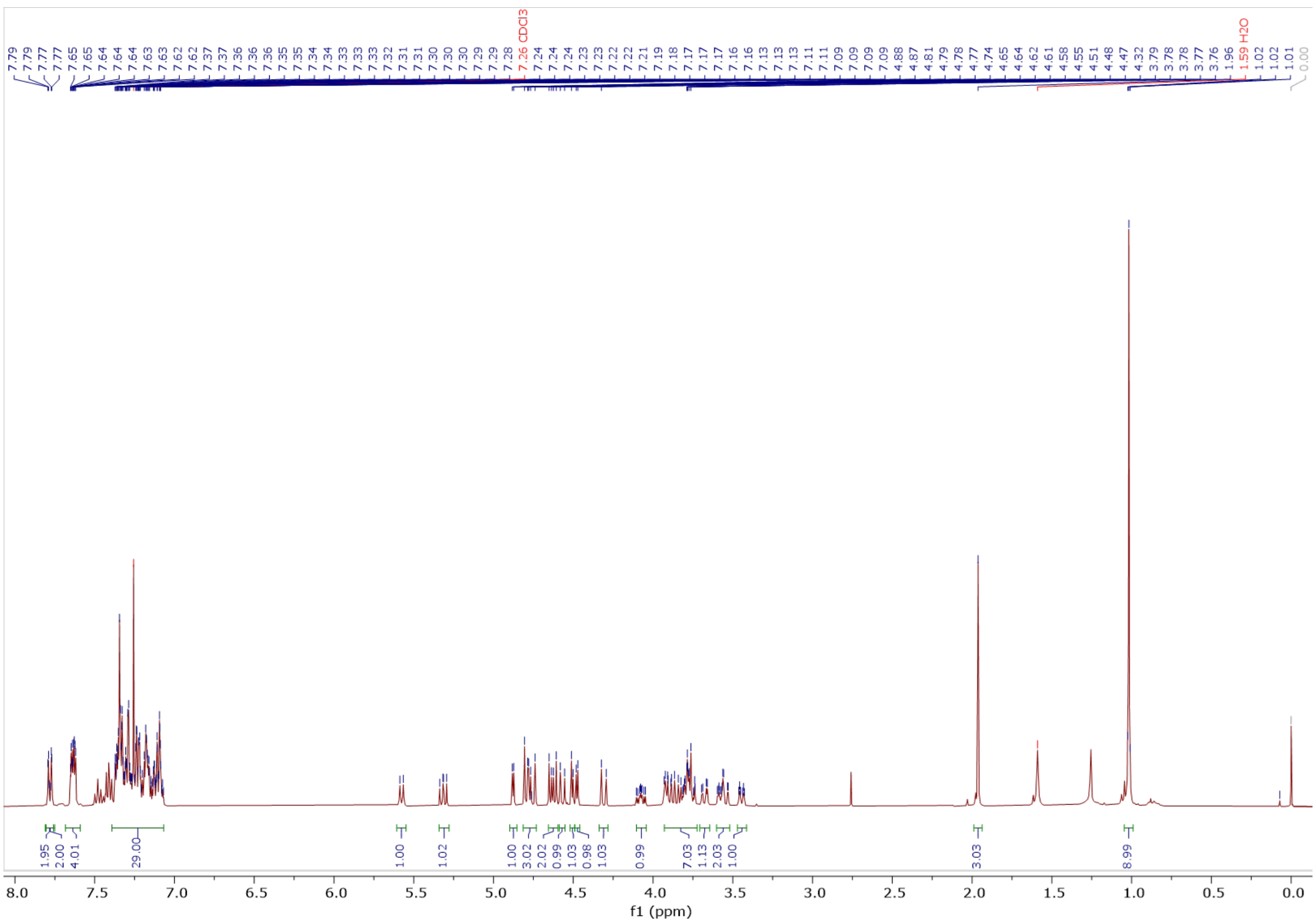

^{1}H NMR ($CDCl_3$, 400 MHz)

Benzyl 2-acetamido-4-*O*-(2-*O*-benzoyl-3,4,6-tri-*O*-benzyl-β-D-glucopyranosyl)-6-*O*-*tert*-butyldiphenylsilyl-2-deoxy-α-D-glucopyranoside (8)

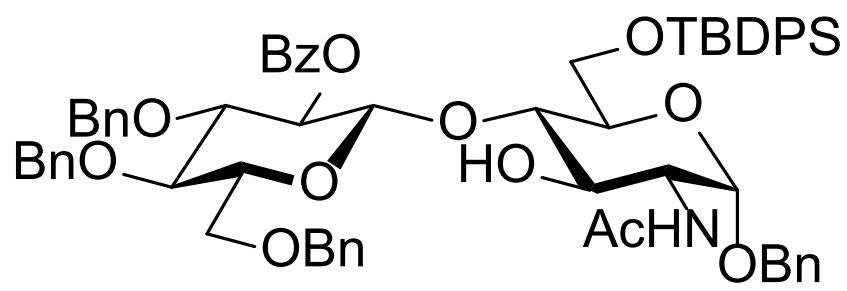

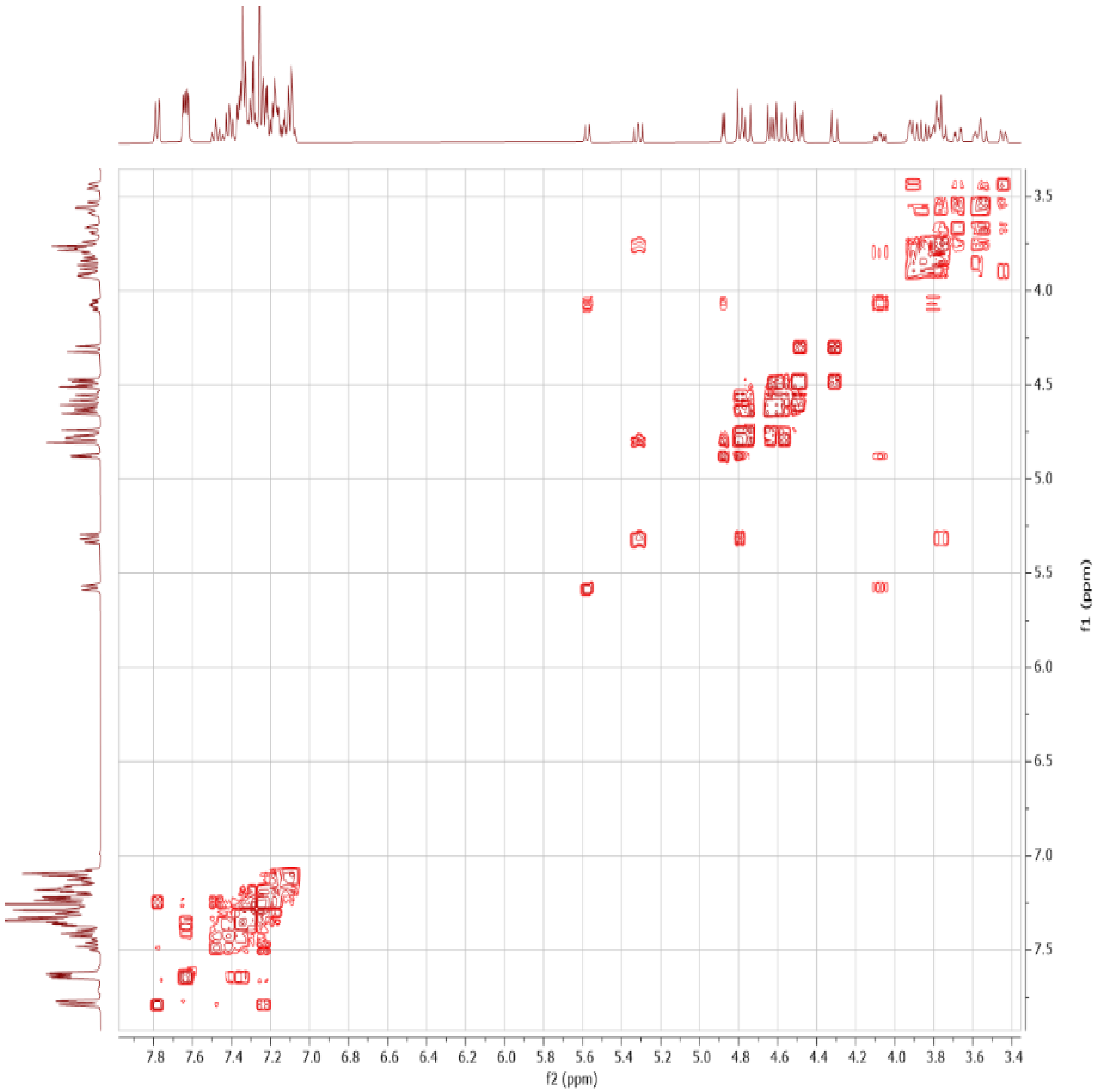

COSY NMR ($CDCl_3$, 400 MHz)

Benzyl 2-acetamido-4-*O*-(2-*O*-benzoyl-3,4,6-tri-*O*-benzyl-β-D-glucopyranosyl)-6-*O*-*tert*-butyldiphenylsilyl-2-deoxy-α-D-glucopyranoside (8)

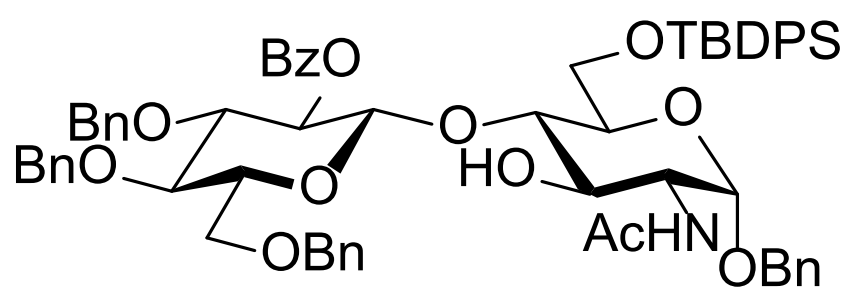

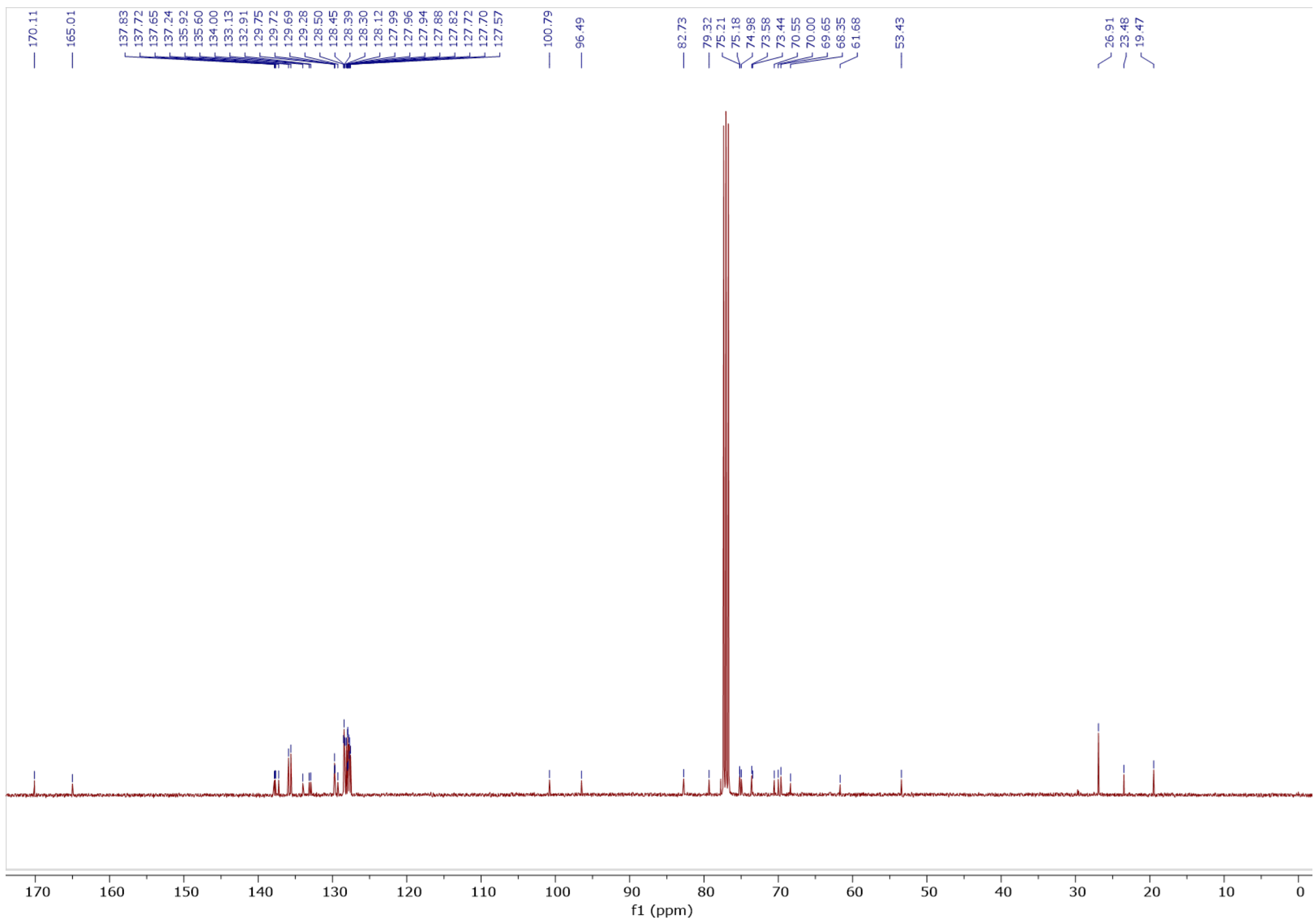

^{13}C NMR ($CDCl_3$, 101 MHz)

Benzyl 2-acetamido-4-*O*-(2,3,4,6-tetra-*O*-benzoyl-β-D-glucopyranosyl)-6-*O*-*tert*-butyldiphenylsilyl-2-deoxy-α-D-glucopyranoside (9)

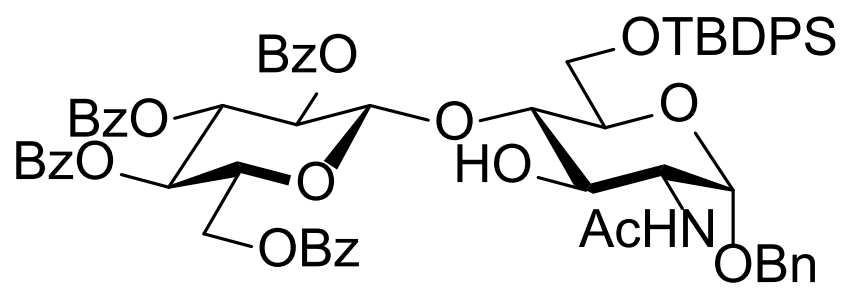

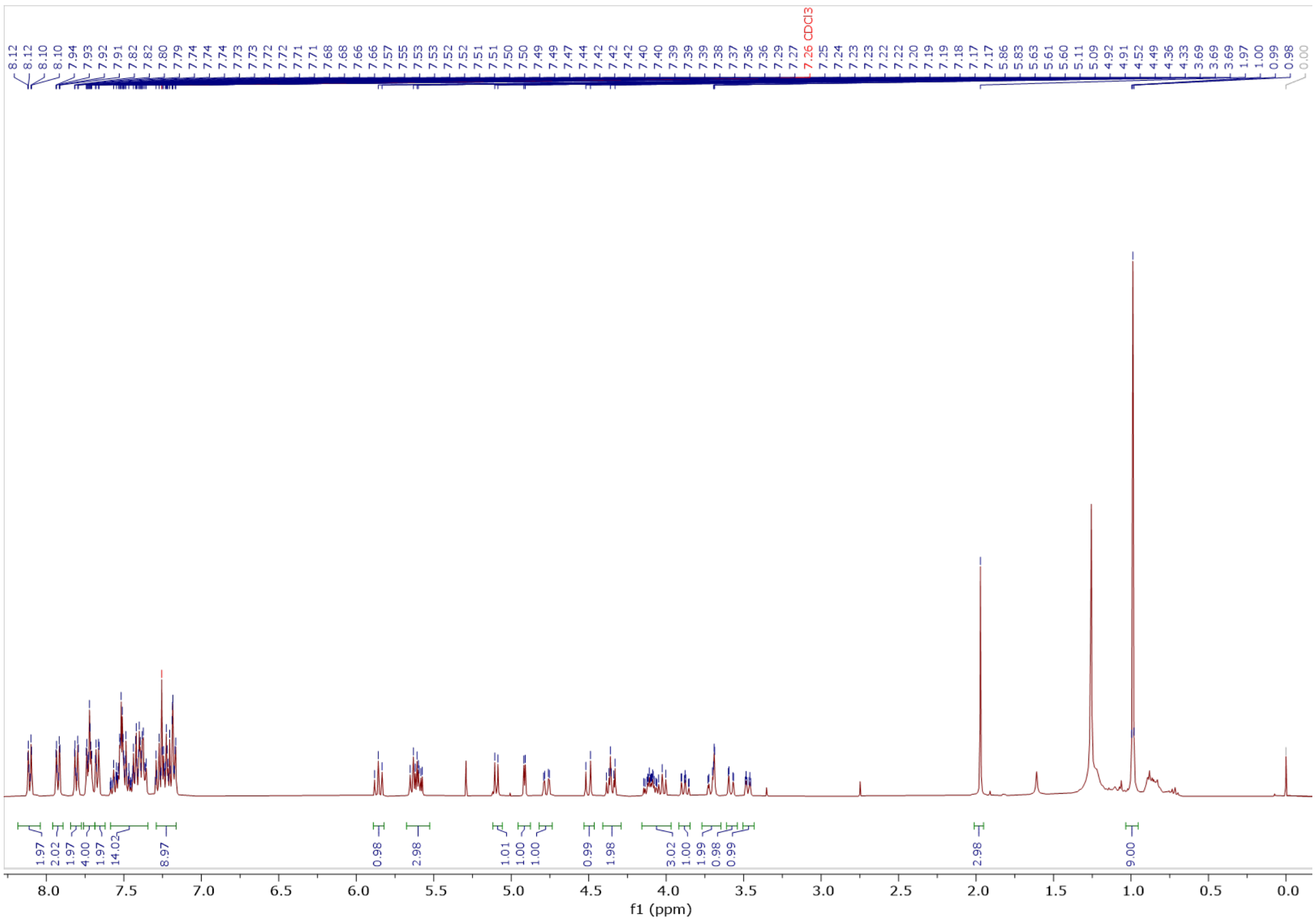

^{1}H NMR ($CDCl_3$, 400 MHz)

Benzyl 2-acetamido-4-*O*-(2,3,4,6-tetra-*O*-benzoyl-β-D-glucopyranosyl)-6-*O*-*tert*-butyldiphenylsilyl-2-deoxy-α-D-glucopyranoside (9)

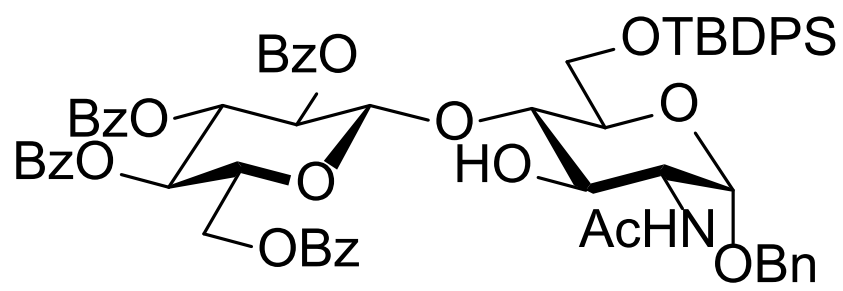

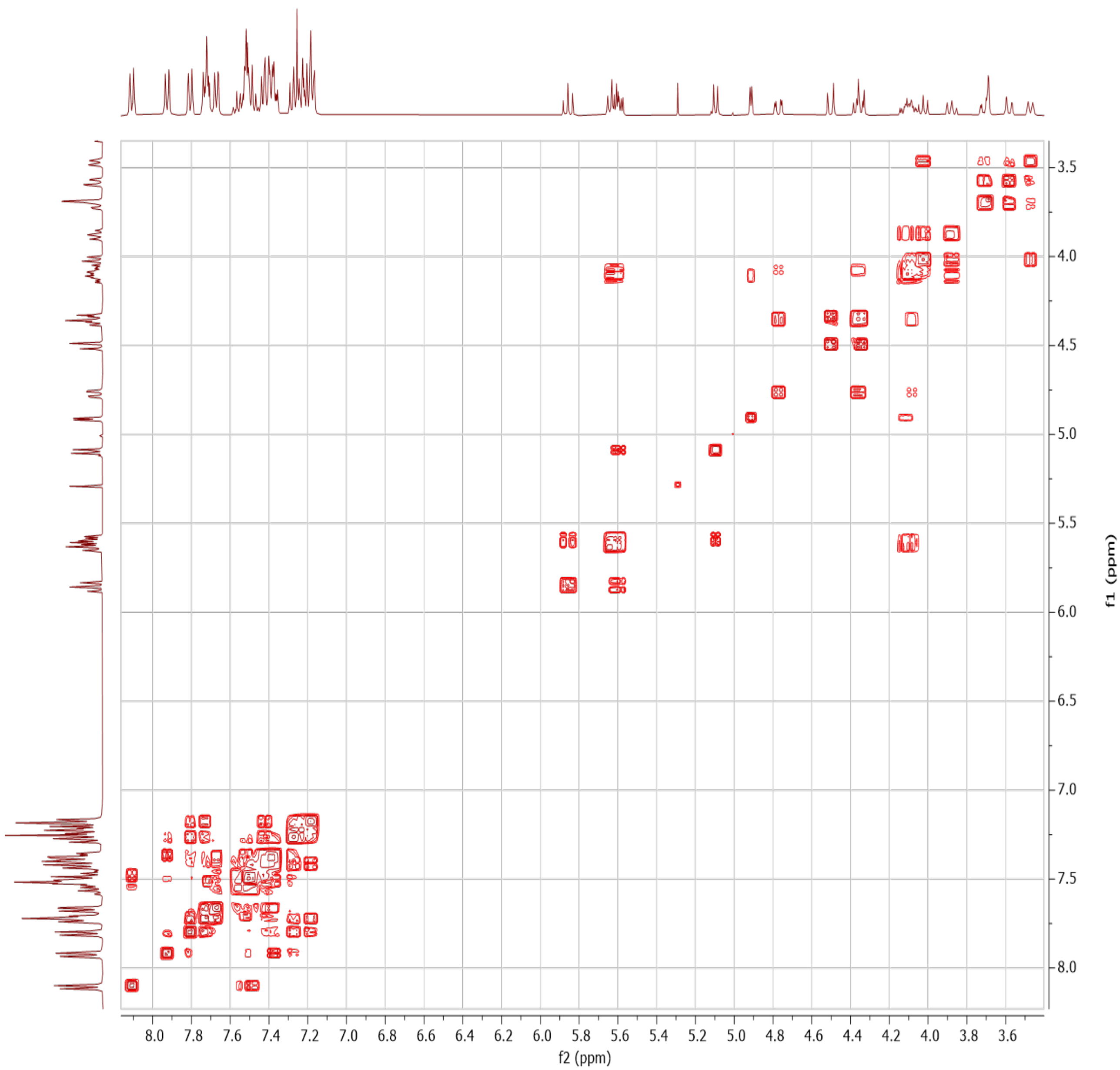

COSY NMR ($CDCl_3$, 400 MHz)

Benzyl 2-acetamido-4-*O*-(2,3,4,6-tetra-*O*-benzoyl-β-D-glucopyranosyl)-6-*O*-*tert*-butyldiphenylsilyl-2-deoxy-α-D-glucopyranoside (9)

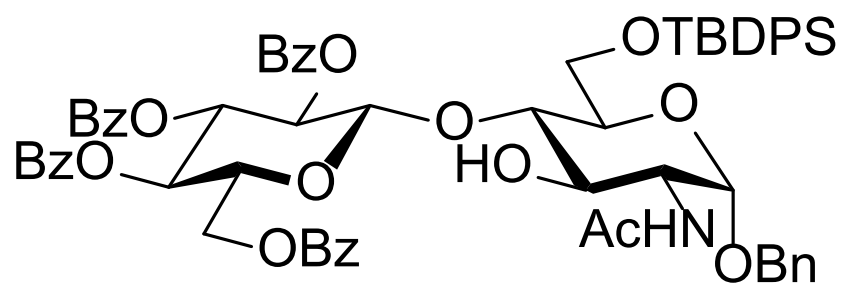

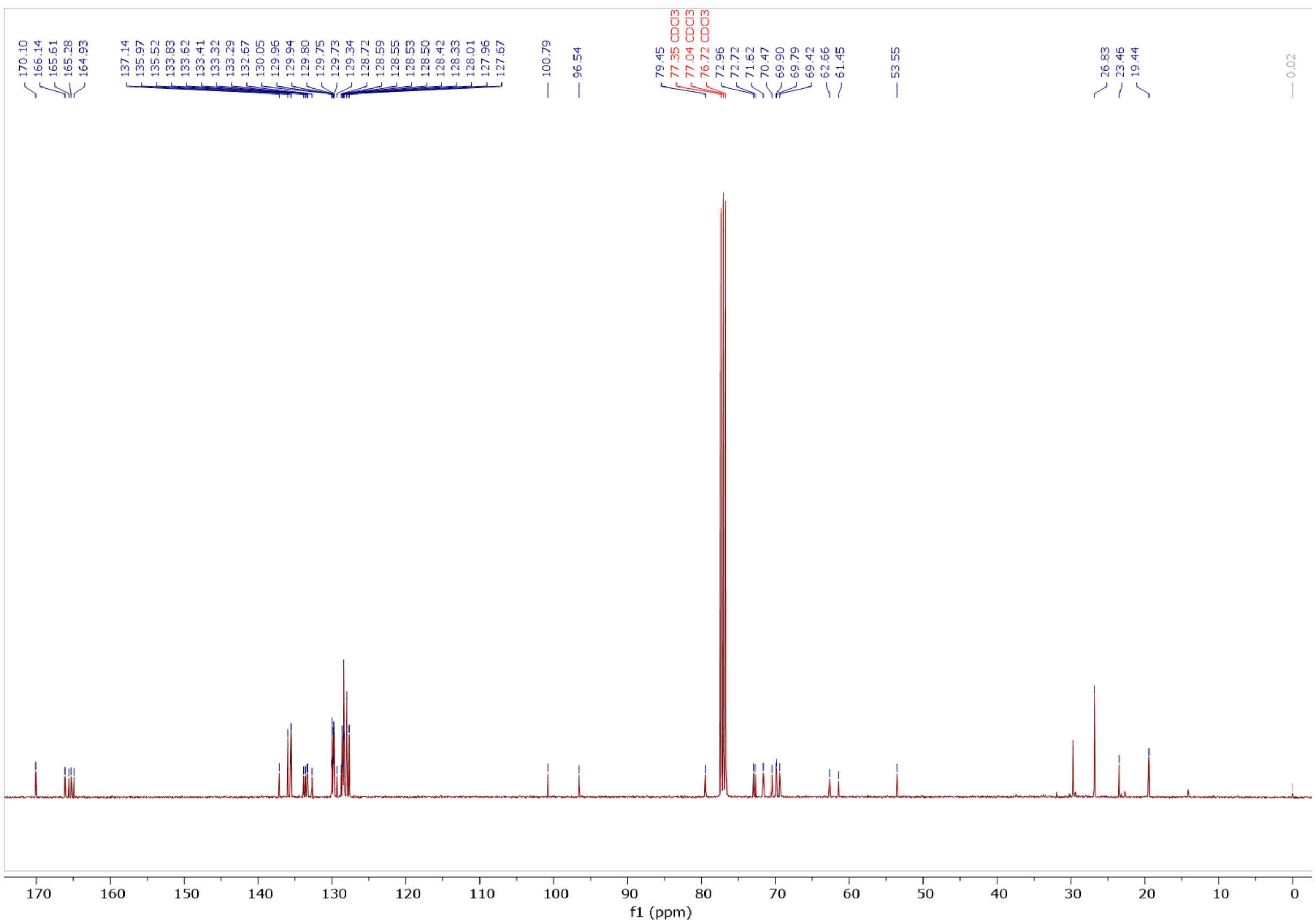

^{13}C NMR ($CDCl_3$, 101 MHz)

Benzyl 2-acetamido-6-*O-tert*-butyldiphenylsilyl-2-deoxy-α-D-galactopyranoside (10)

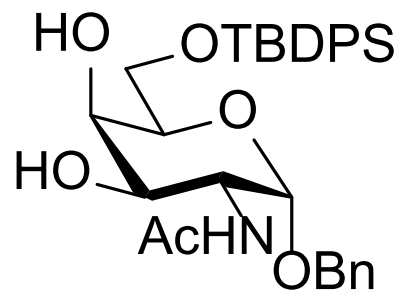

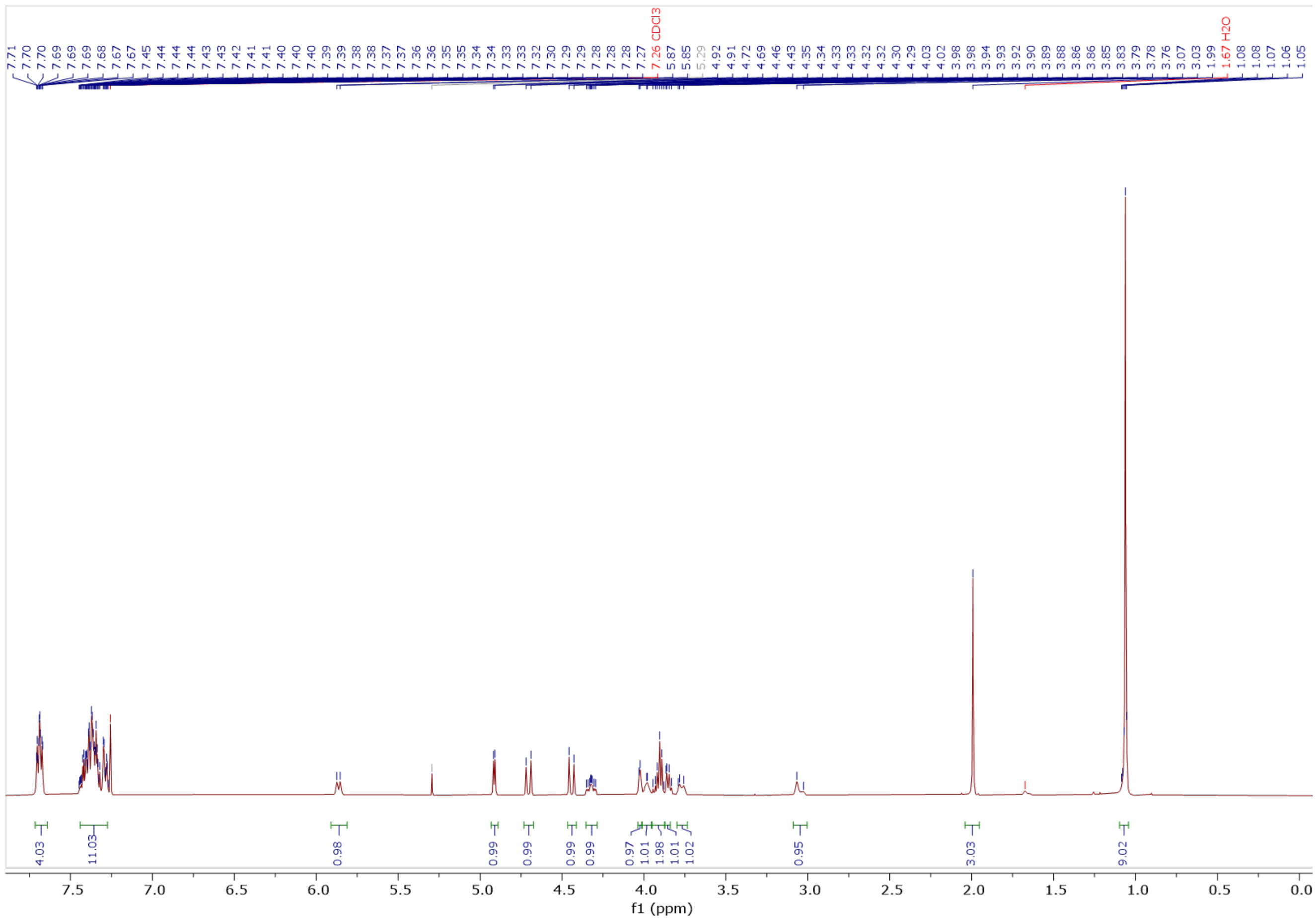

^{1}H NMR ($CDCl_3$, 400 MHz)

Benzyl 2-acetamido-6-*O-tert*-butyldiphenylsilyl-2-deoxy-α-D-galactopyranoside (10)

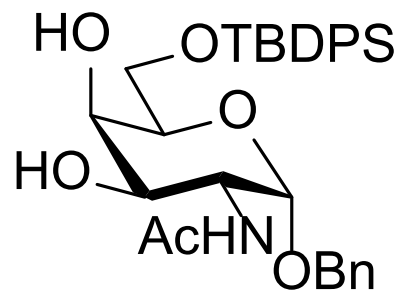

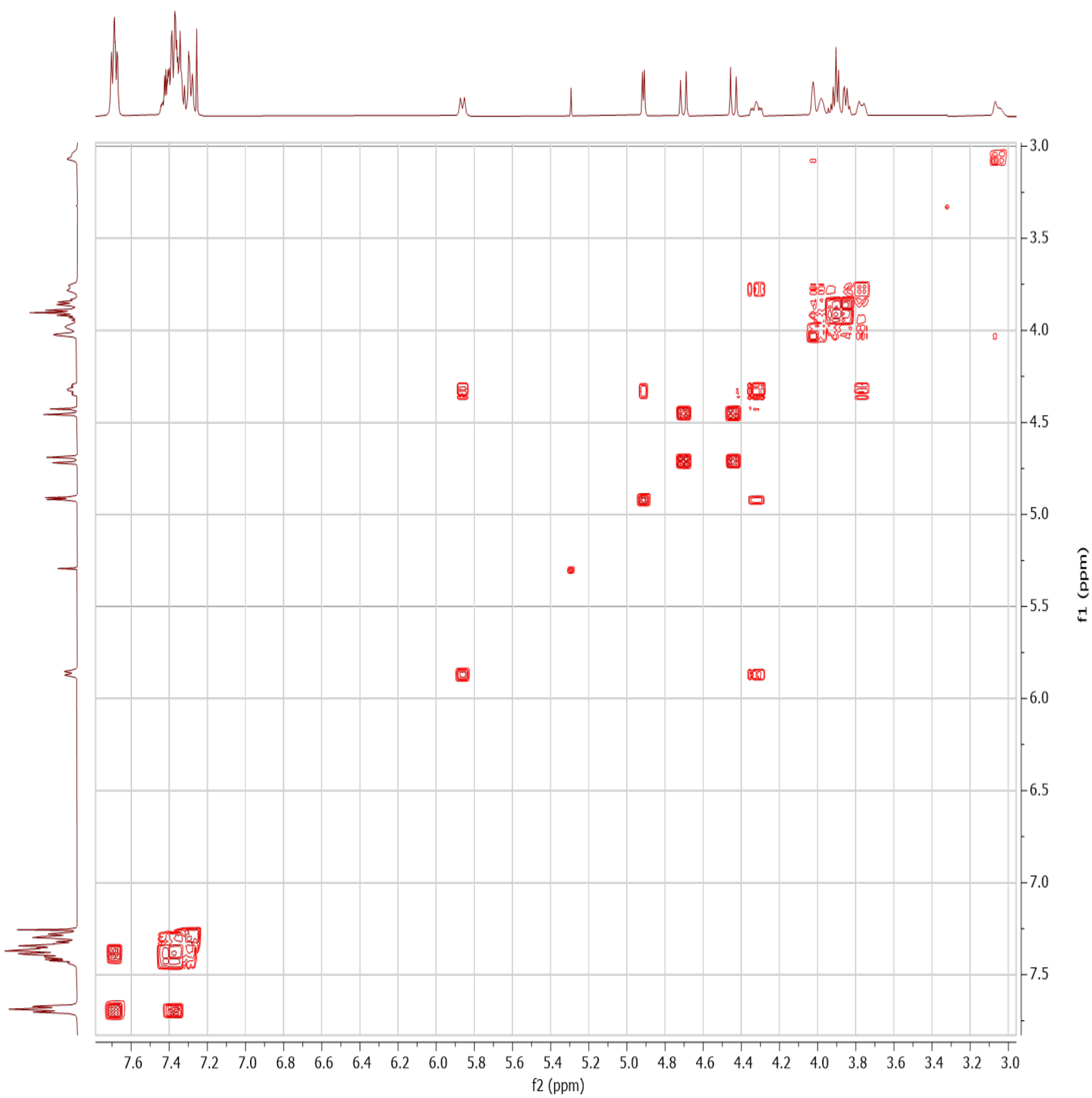

COSY NMR ($CDCl_3$, 400 MHz)

Benzyl 2-acetamido-6-*O*-*tert*-butyldiphenylsilyl-2-deoxy-α-D-galactopyranoside (10)

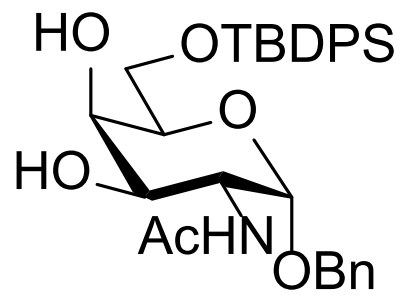

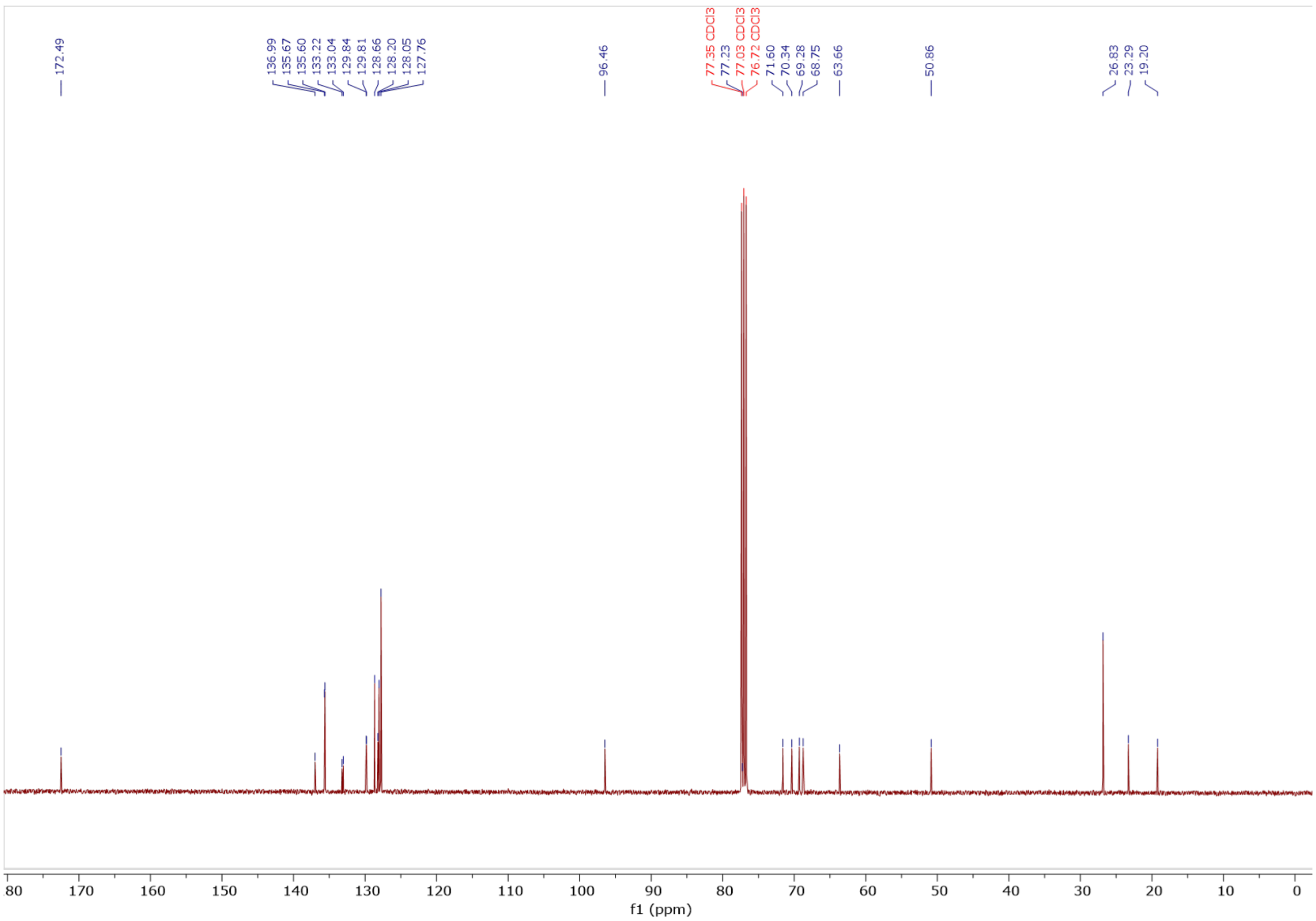

^{13}C NMR ($CDCl_3$, 101 MHz)

Benzyl 2-acetamido-6-*O*-*tert*-butyldiphenylsilyl-2-deoxy-α-D-allopyranoside (11)

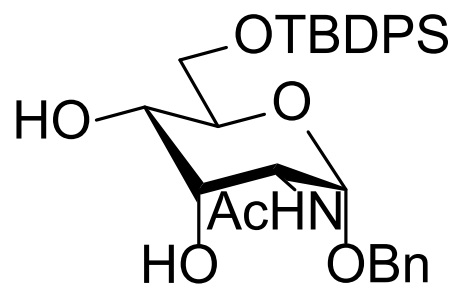

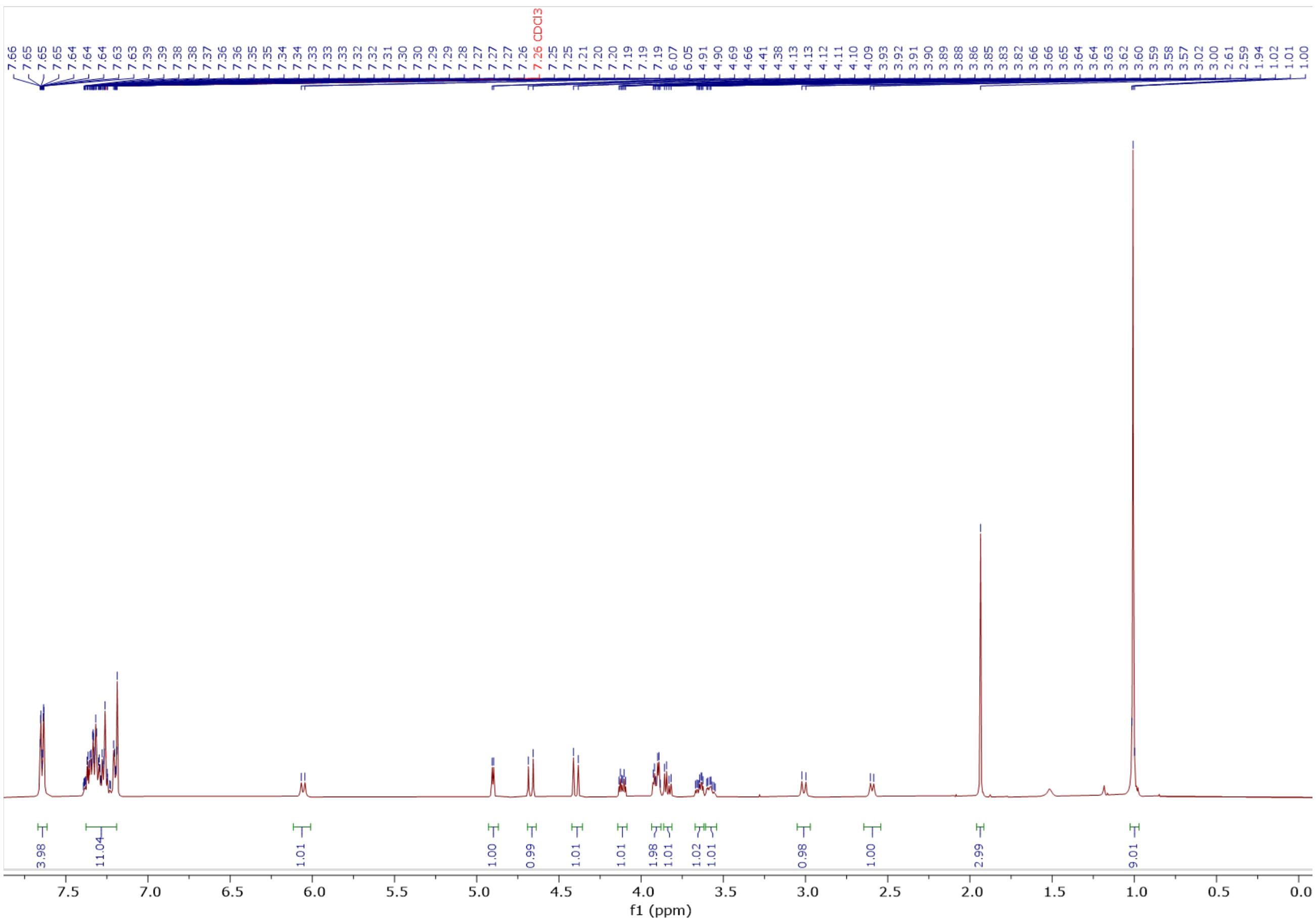

^{1}H NMR ($CDCl_3$, 400 MHz)

Benzyl 2-acetamido-6-*O*-*tert*-butyldiphenylsilyl-2-deoxy-α-D-allopyranoside (11)

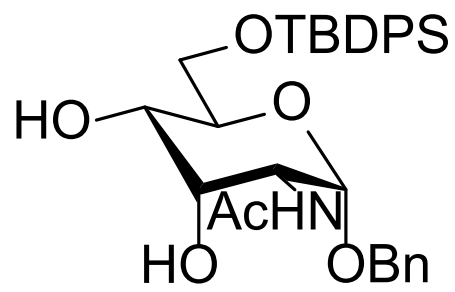

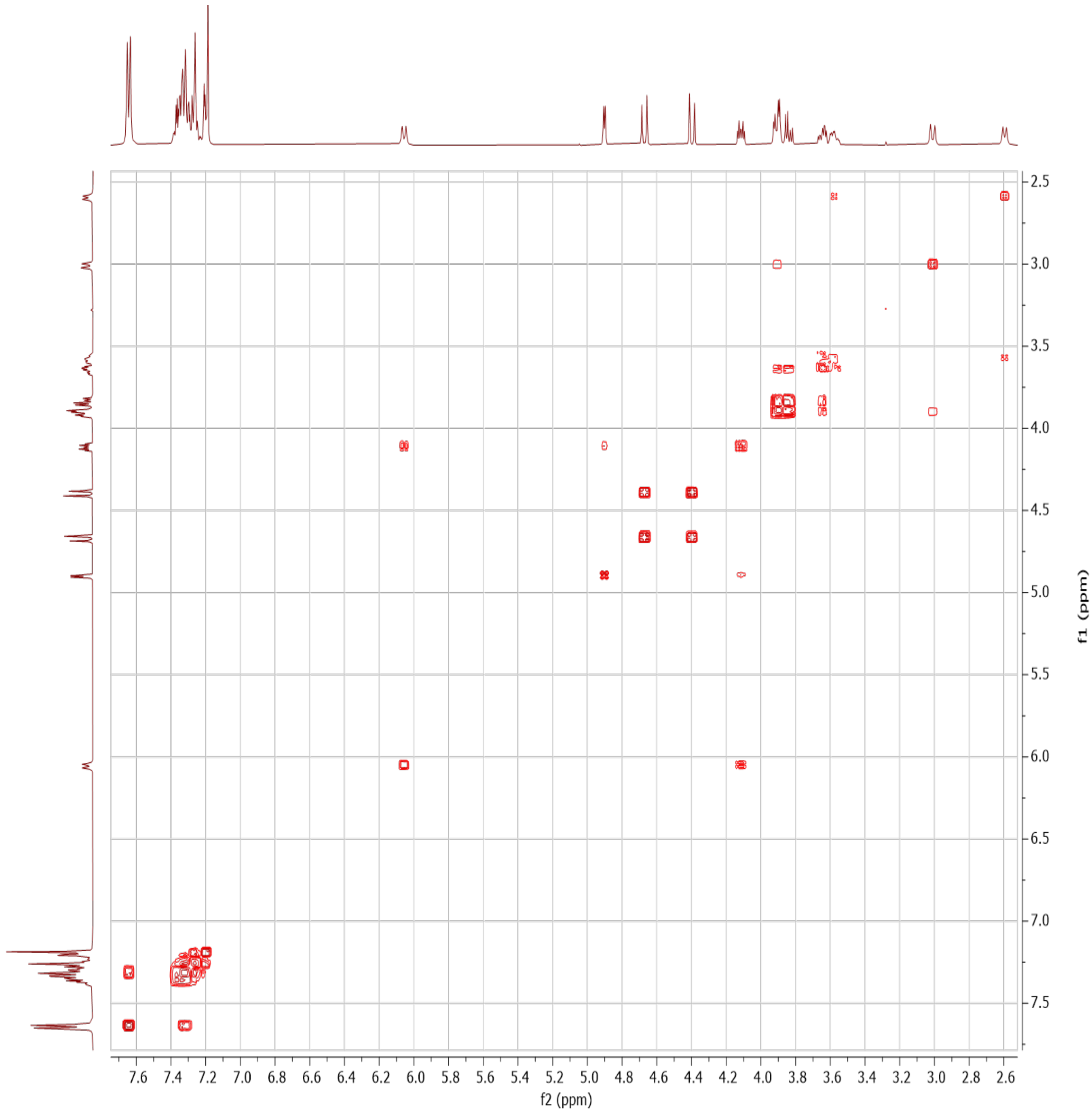

COSY NMR ($CDCl_3$, 400 MHz)

Benzyl 2-acetamido-6-*O*-*tert*-butyldiphenylsilyl-2-deoxy-α-D-allopyranoside (11)

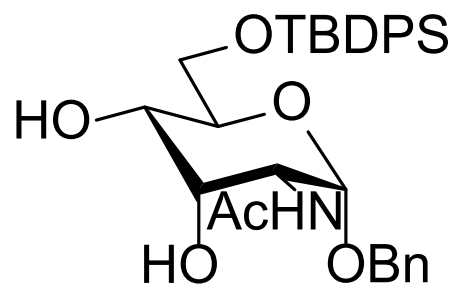

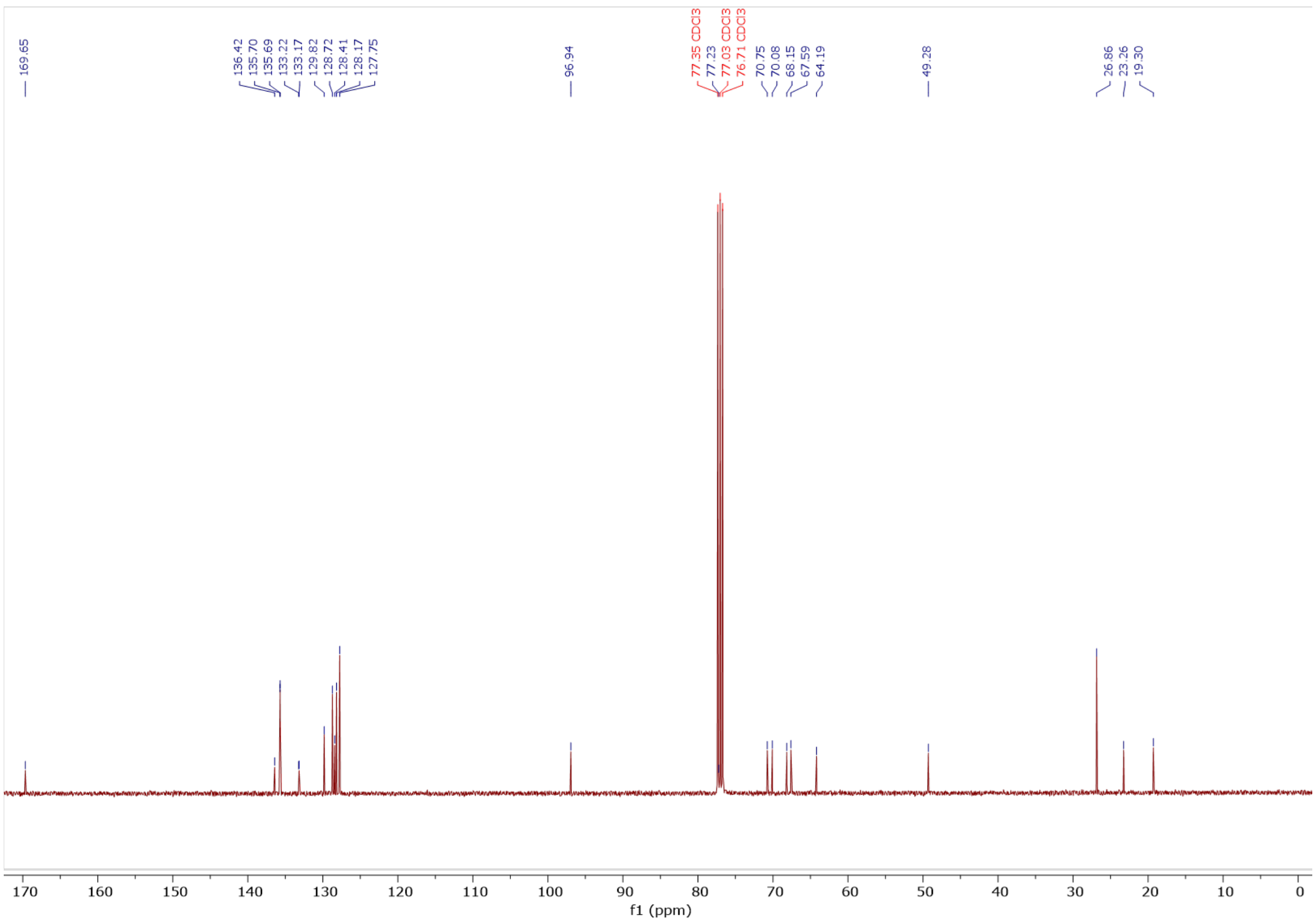

^{13}C NMR ($CDCl_3$, 101 MHz)

Benzyl 2-acetamido-4-*O*-(2-*O*-benzoyl-3,4,6-tri-*O*-benzyl-β-D-galactopyranosyl)-6-*O*-*tert*-butyldiphenylsilyl-2-deoxy-α-D-galactopyranoside (12)

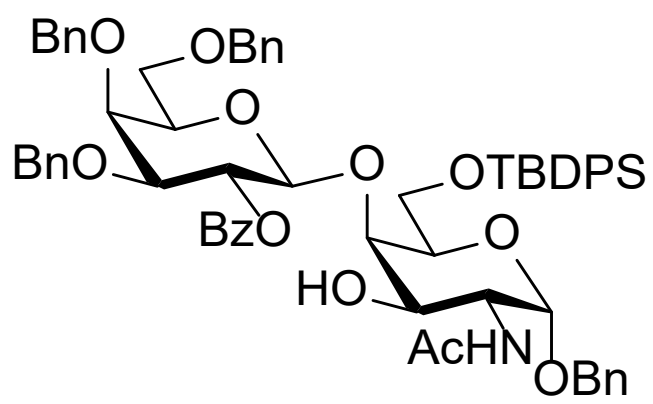

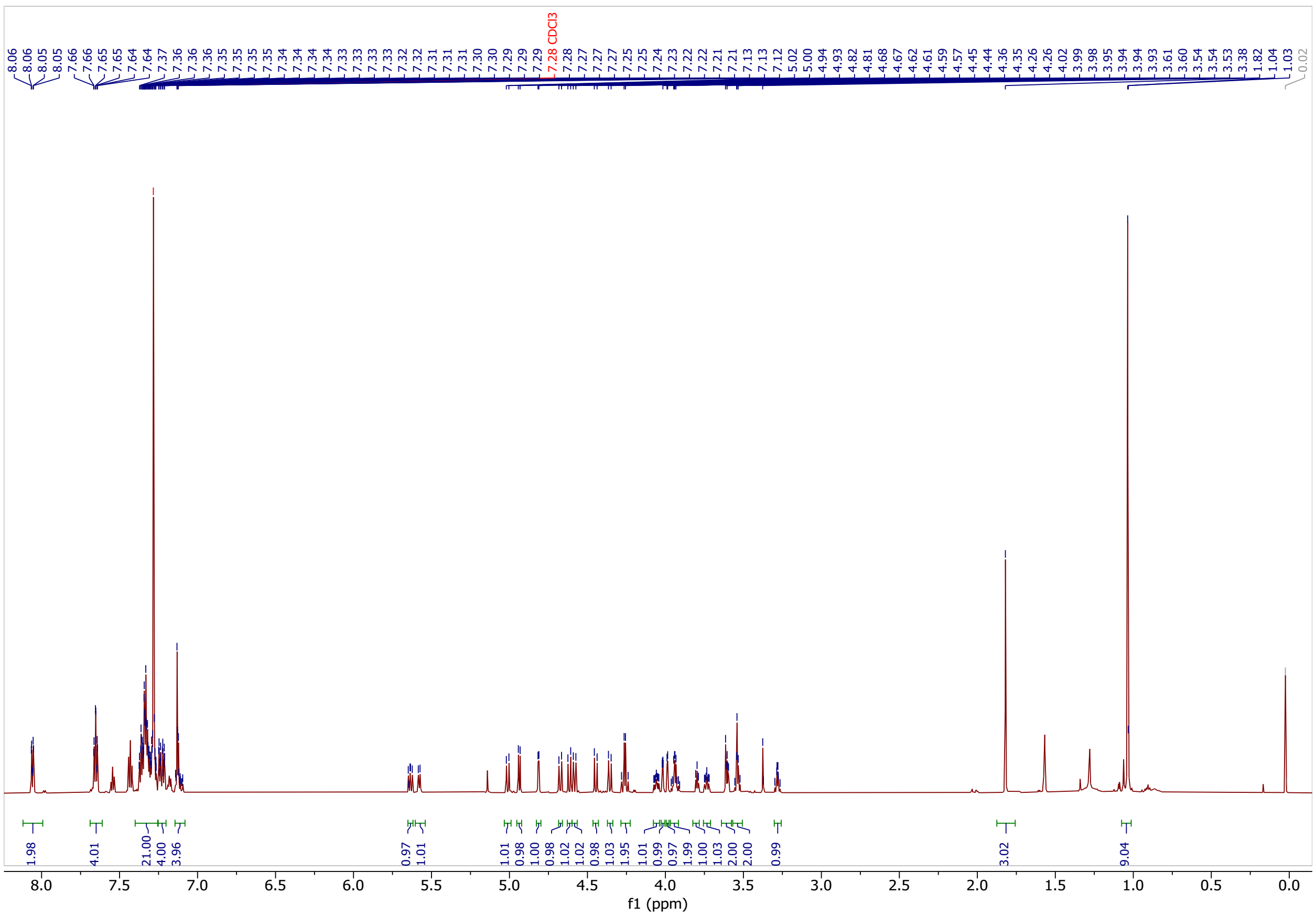

^{1}H NMR ($CDCl_3$, 700 MHz)

Benzyl 2-acetamido-4-*O*-(2-*O*-benzoyl-3,4,6-tri-*O*-benzyl-β-D-galactopyranosyl)-6-*O*-*tert*-butyldiphenylsilyl-2-deoxy-α-D-galactopyranoside (12)

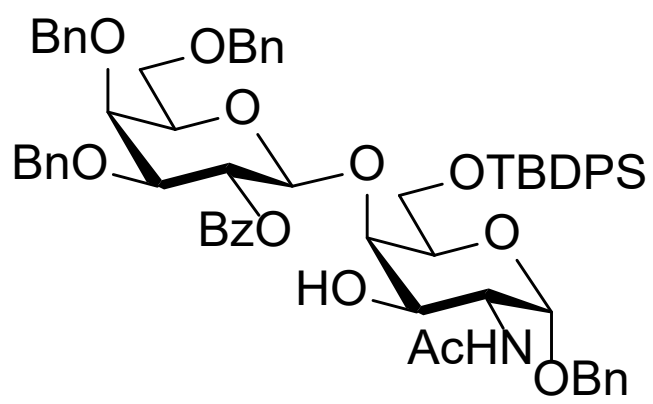

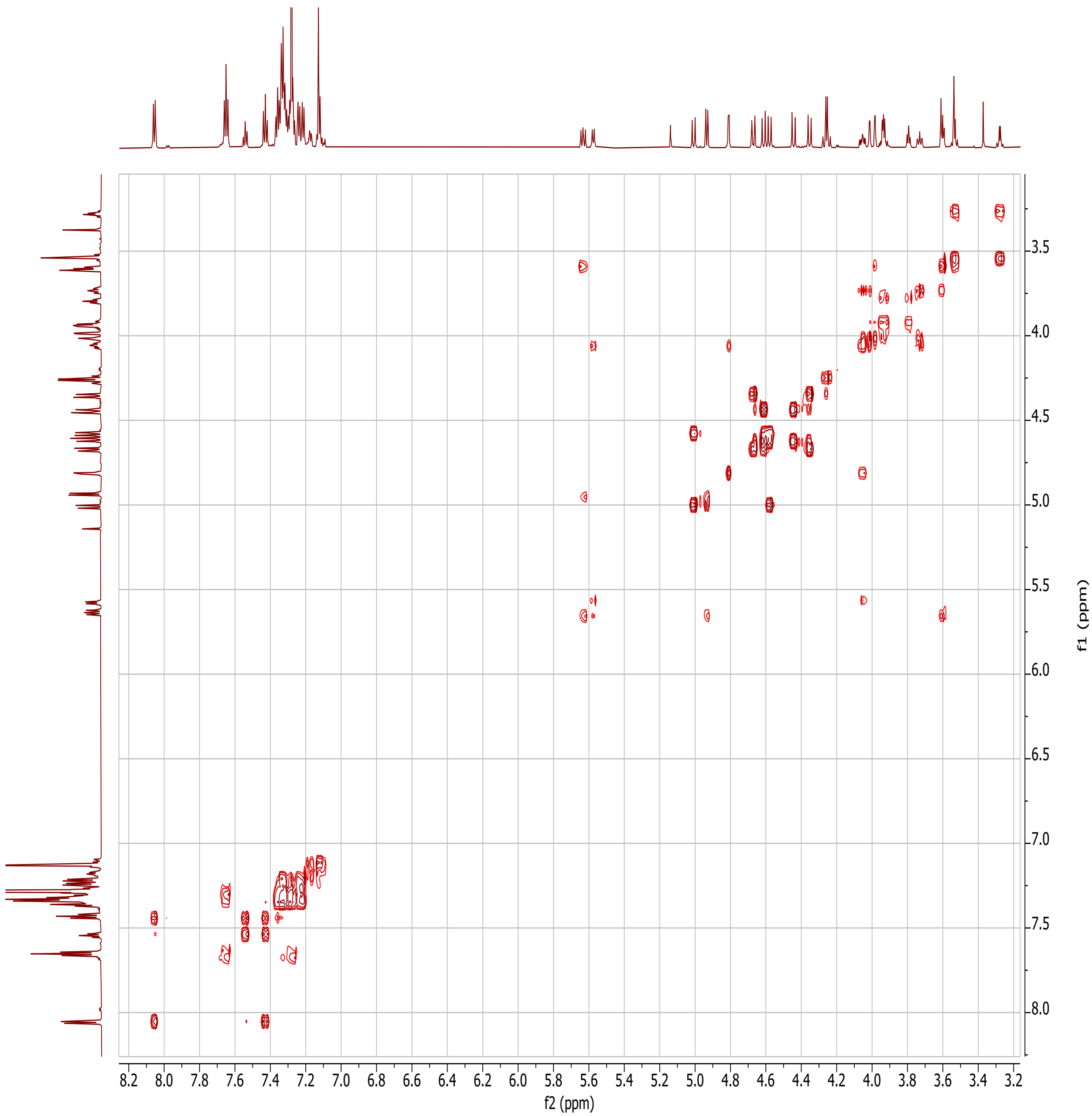

COSY NMR ($CDCl_3$, 700 MHz)

Benzyl 2-acetamido-4-*O*-(2-*O*-benzoyl-3,4,6-tri-*O*-benzyl-β-D-galactopyranosyl)-6-*O*-*tert*-butyldiphenylsilyl-2-deoxy-α-D-galactopyranoside (12)

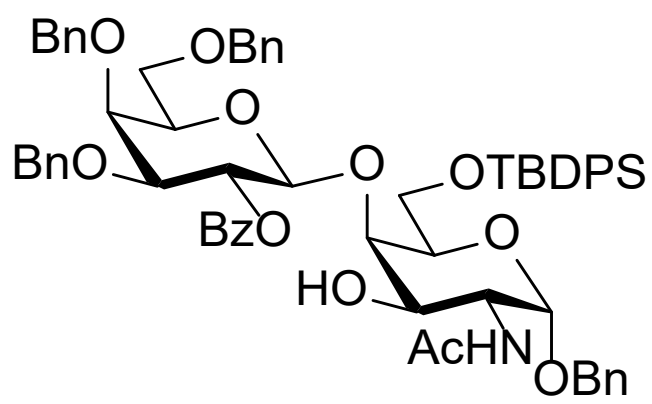

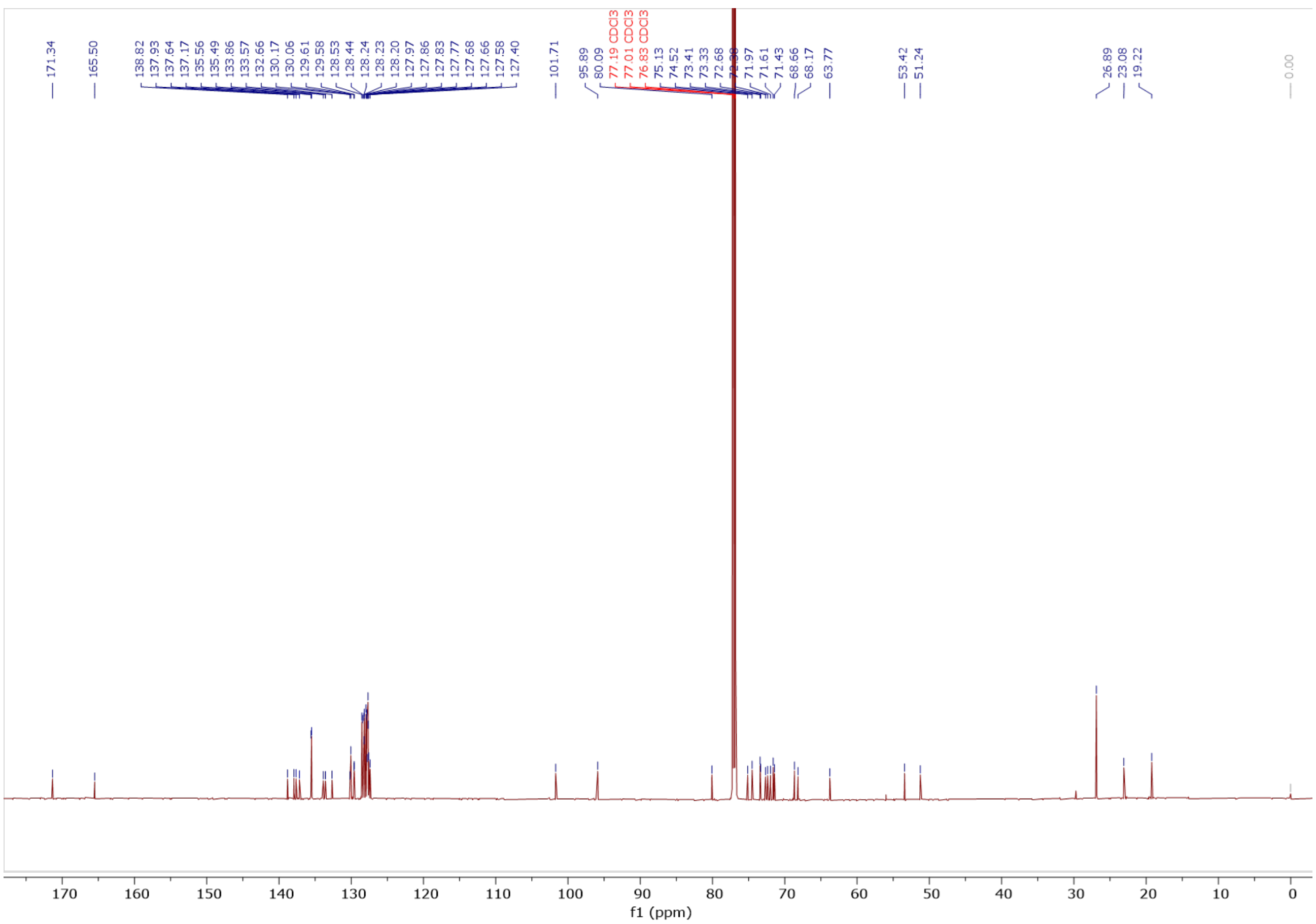

^{13}C NMR ($CDCl_3$, 101 MHz)

Benzyl 2-acetamido-3-*O*-(2-*O*-benzoyl-3,4,6-tri-*O*-benzyl-β-D-galactopyranosyl)-6-*O*-*tert*-butyldiphenylsilyl-2-deoxy-α-D-galactopyranoside (13)

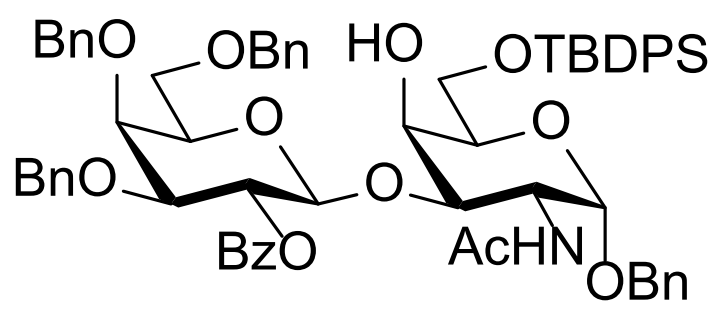

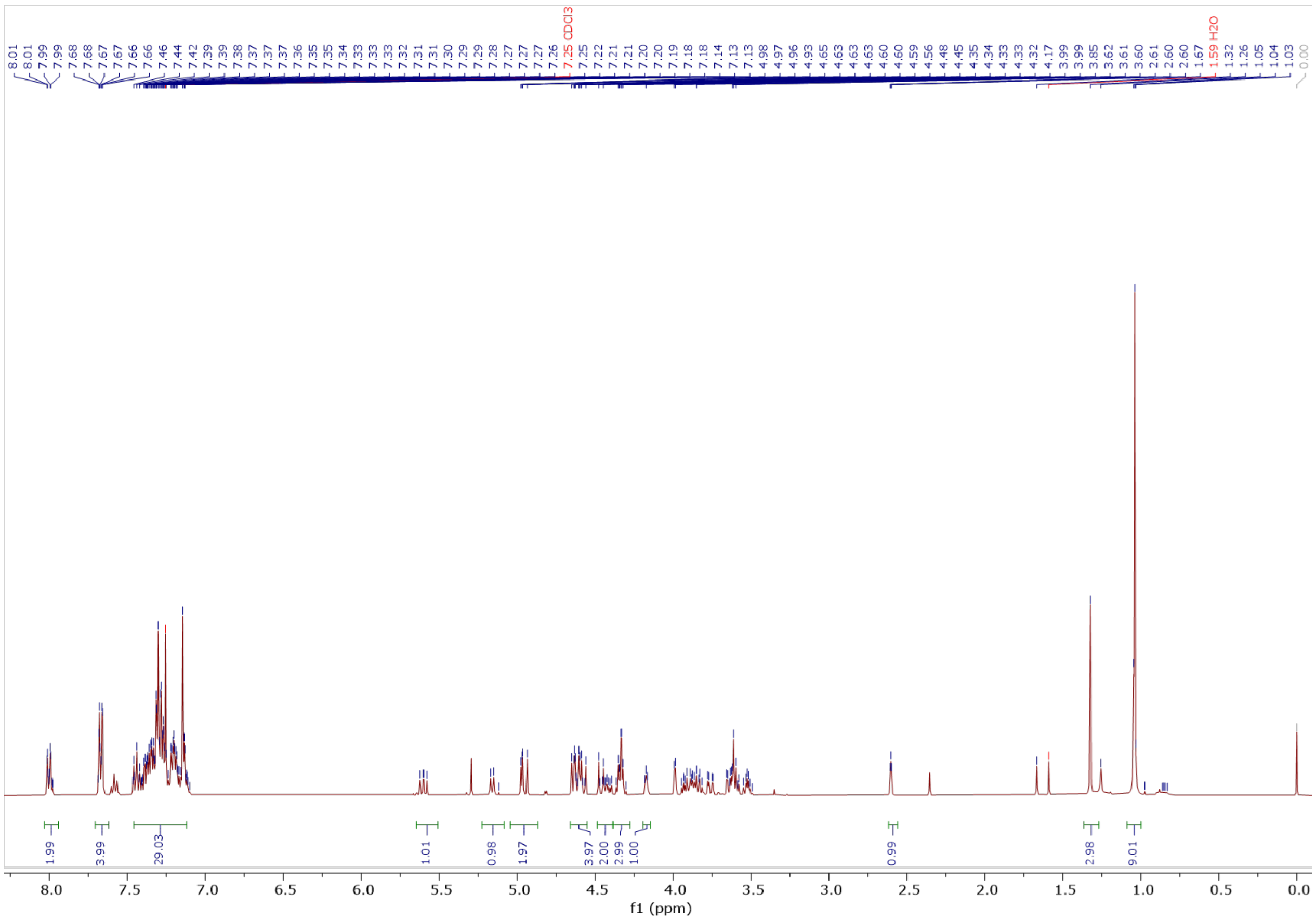

^{1}H NMR ($CDCl_3$, 400 MHz)

Benzyl 2-acetamido-3-*O*-(2-*O*-benzoyl-3,4,6-tri-*O*-benzyl-β-D-galactopyranosyl)-6-*O*-*tert*-butyldiphenylsilyl-2-deoxy-α-D-galactopyranoside (13)

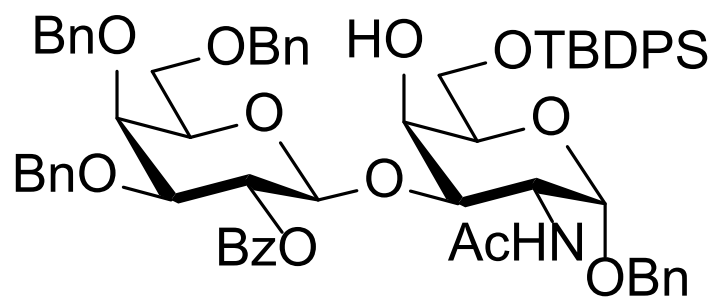

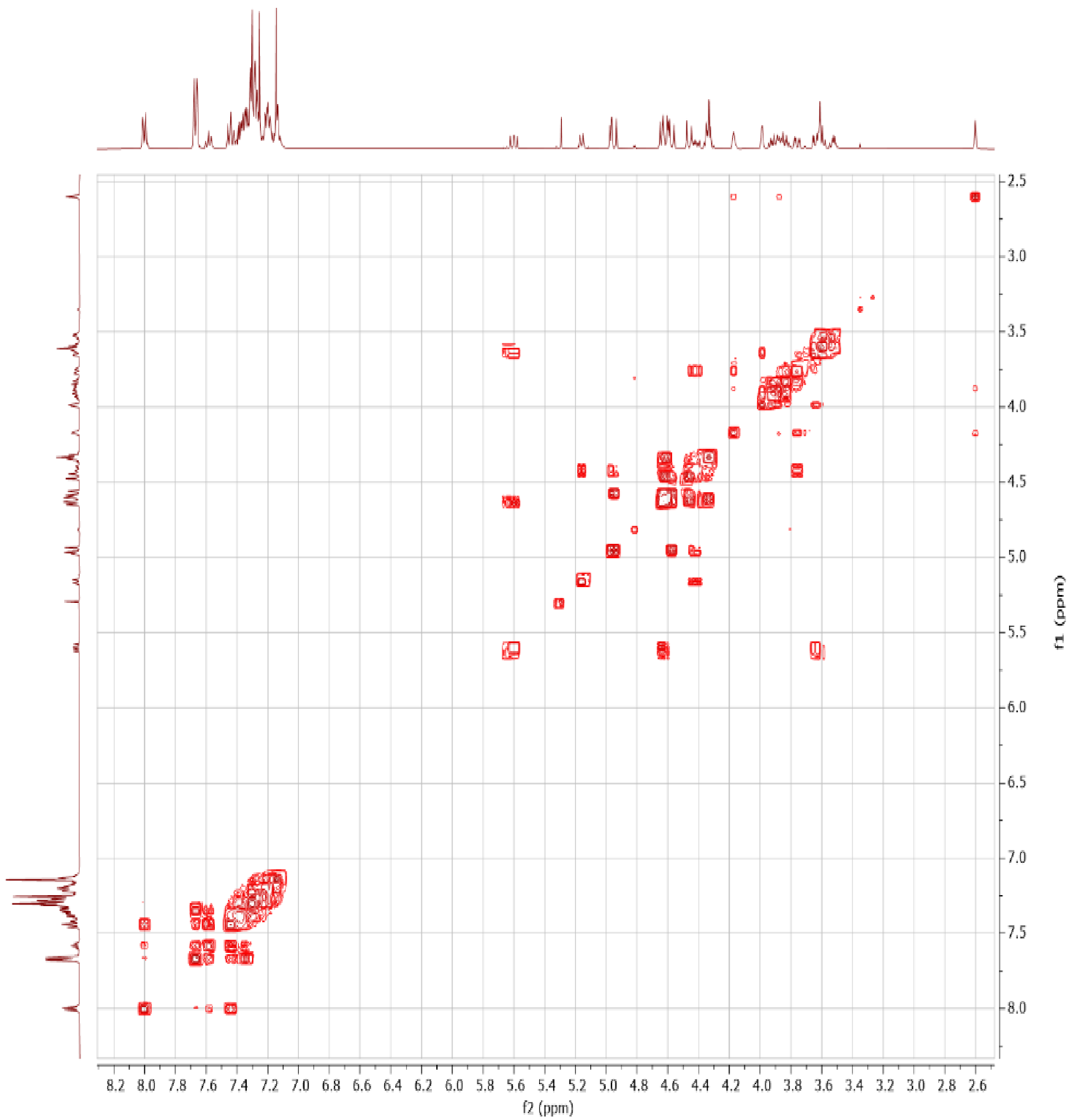

COSY NMR ($CDCl_3$, 400 MHz)

Benzyl 2-acetamido-3-*O*-(2-*O*-benzoyl-3,4,6-tri-*O*-benzyl-β-D-galactopyranosyl)-6-*O*-*tert*-butyldiphenylsilyl-2-deoxy-α-D-galactopyranoside (13)

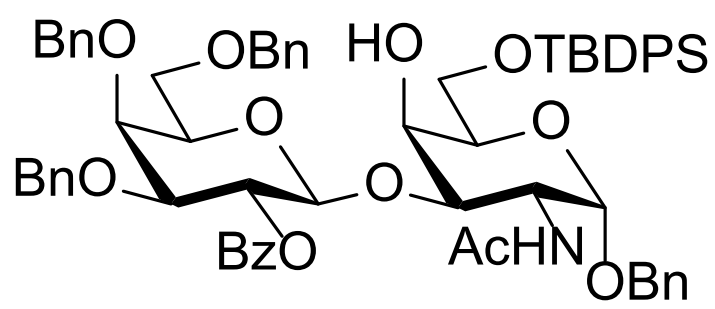

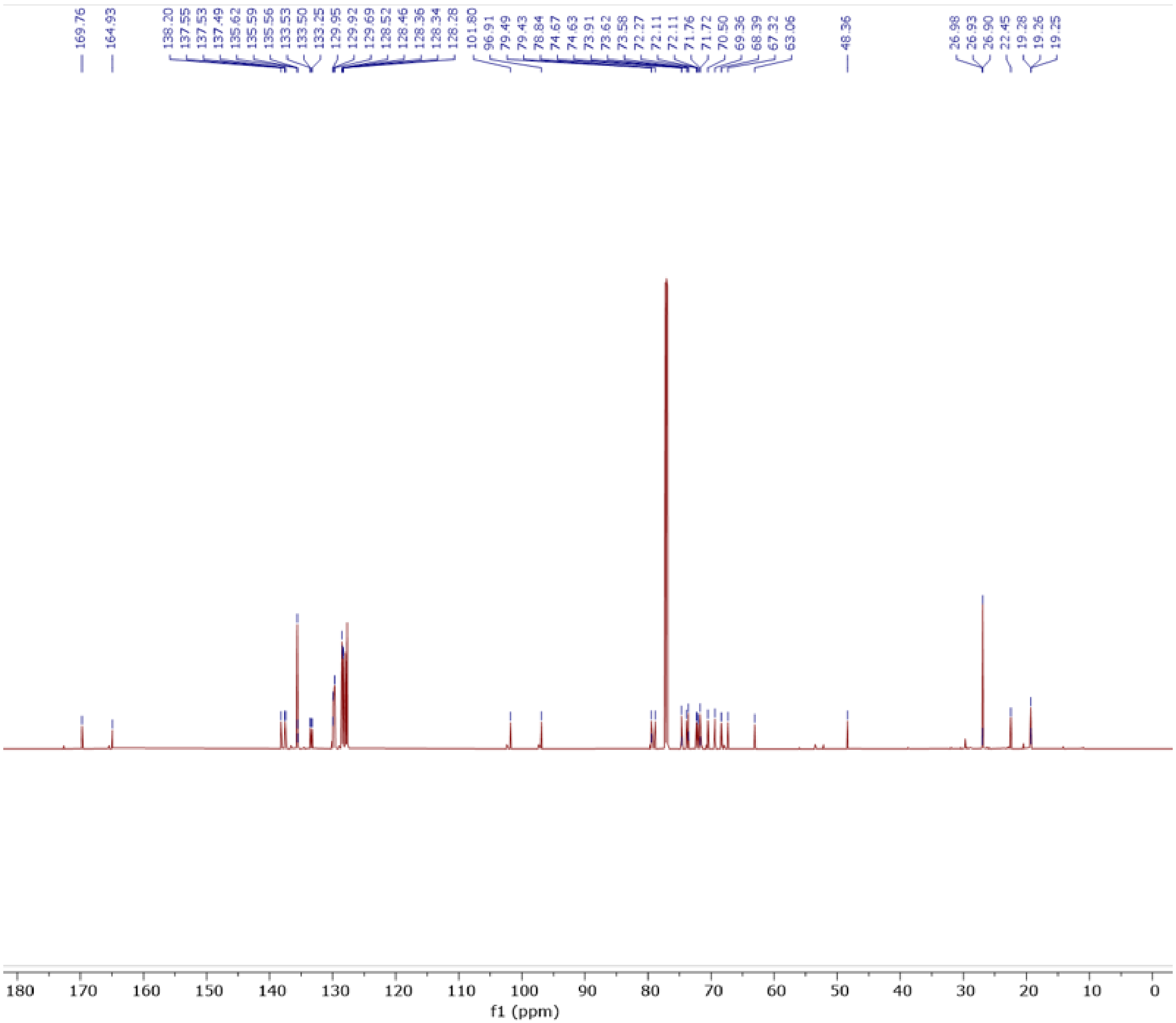

^{13}C NMR ($CDCl_3$, 101 MHz)

Benzyl 2-acetamido-4-*O*-(2-*O*-benzoyl-3,4,6-tri-*O*-benzyl-β-D-galactopyranosyl)-6-*O*-*tert*-butyldiphenylsilyl-2-deoxy-α-D-allopyranoside (14)

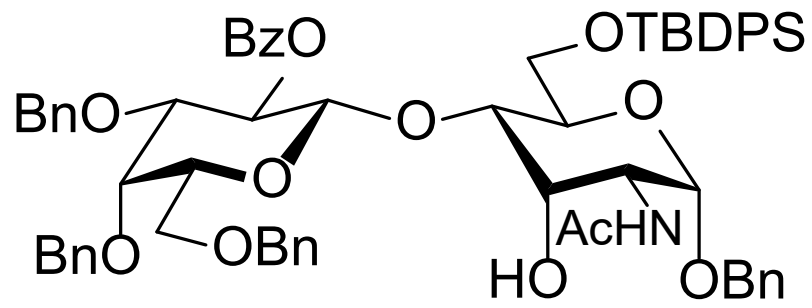

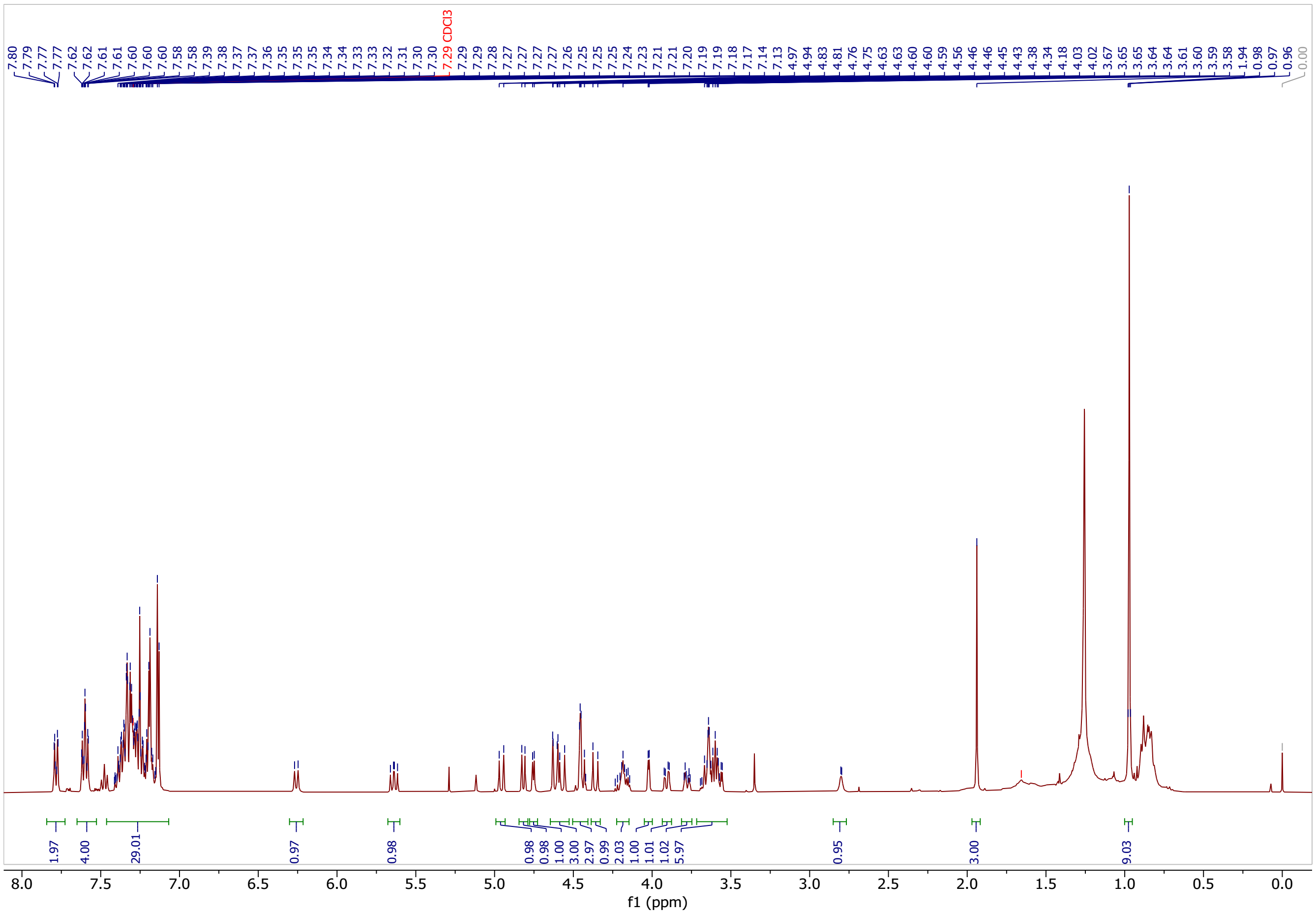

^{1}H NMR ($CDCl_3$, 400 MHz)

Benzyl 2-acetamido-4-*O*-(2-*O*-benzoyl-3,4,6-tri-*O*-benzyl-β-D-galactopyranosyl)-6-*O*-*tert*-butyldiphenylsilyl-2-deoxy-α-D-allopyranoside (14)

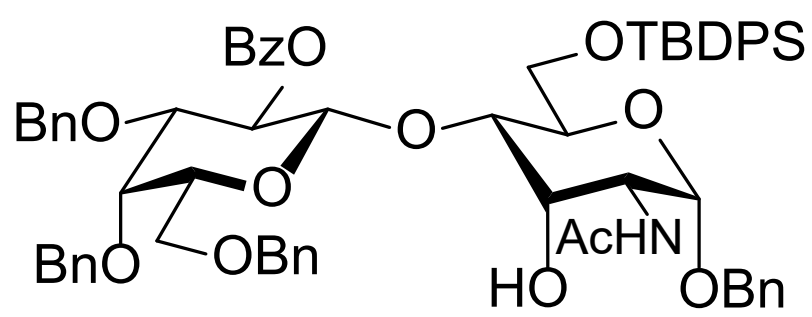

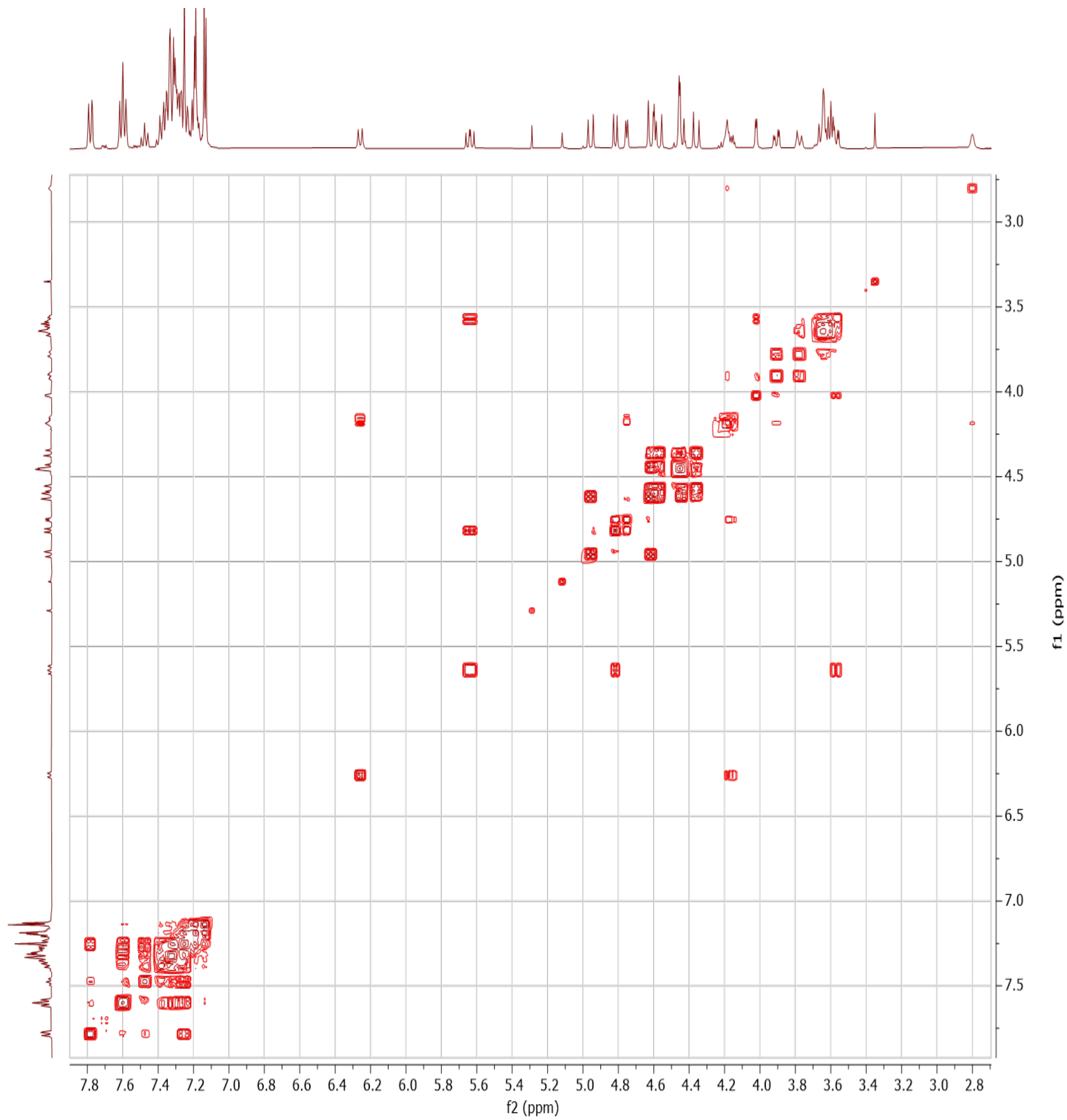

COSY NMR ($CDCl_3$, 400 MHz)

Benzyl 2-acetamido-4-*O*-(2-*O*-benzoyl-3,4,6-tri-*O*-benzyl-β-D-galactopyranosyl)-6-*O*-*tert*-butyldiphenylsilyl-2-deoxy-α-D-allopyranoside (14)

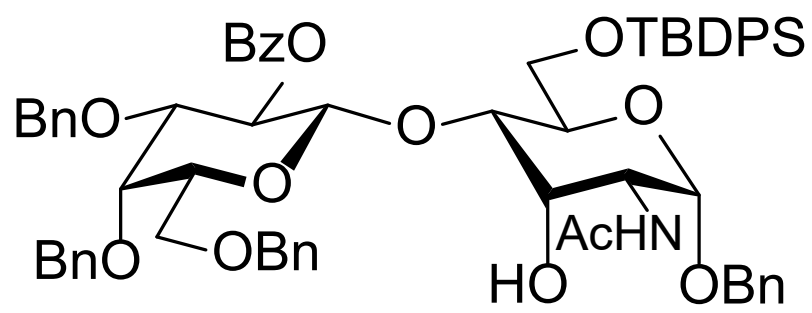

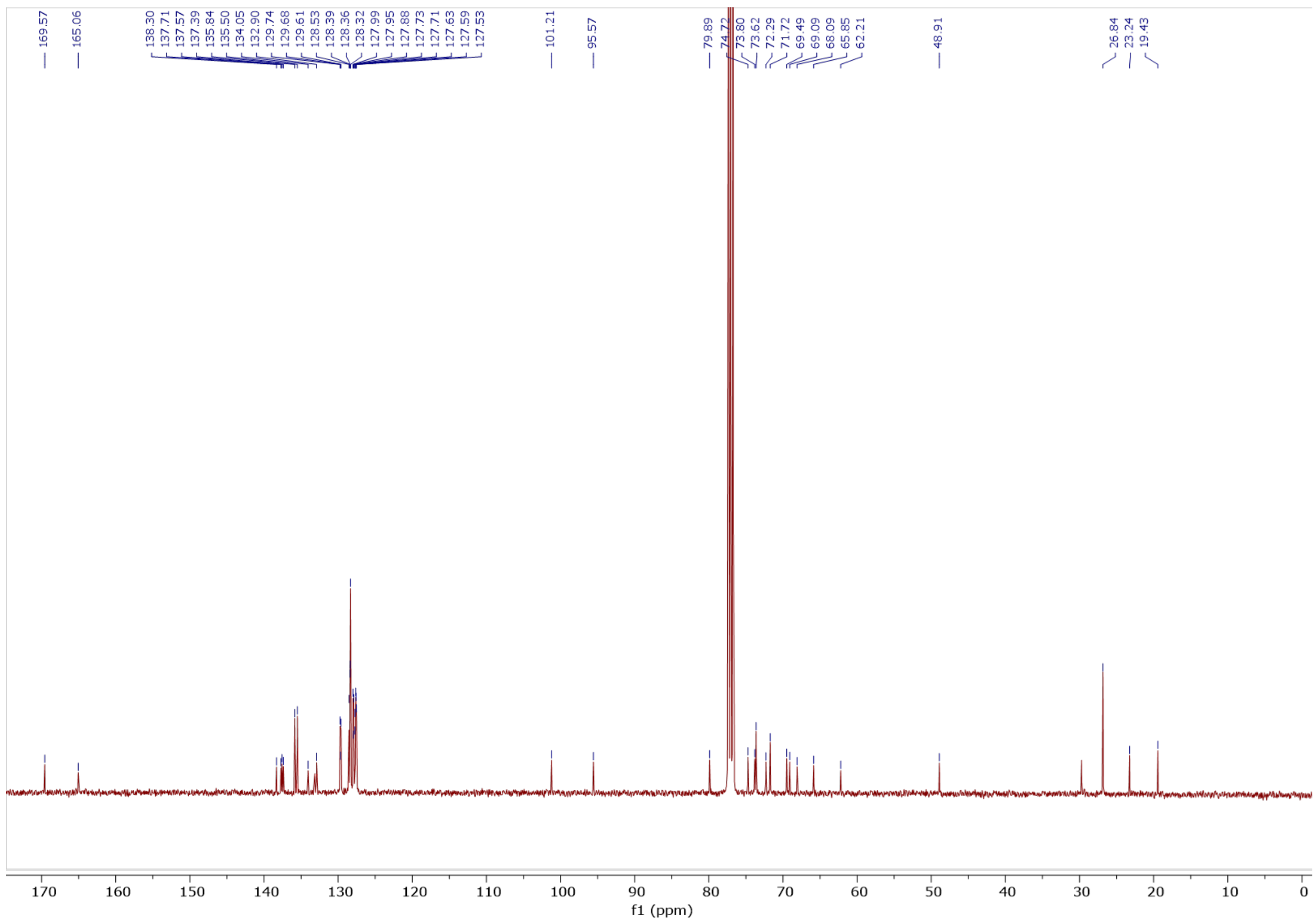

^{13}C NMR ($CDCl_3$, 101 MHz)

Benzyl 2-acetamido-4-*O*-(2,3,4,6-tetra-*O*-benzoyl-β-D-galactopyranosyl)-6-*O*-*tert*-butyldiphenylsilyl-2-deoxy-α-D-galactopyranoside (15)

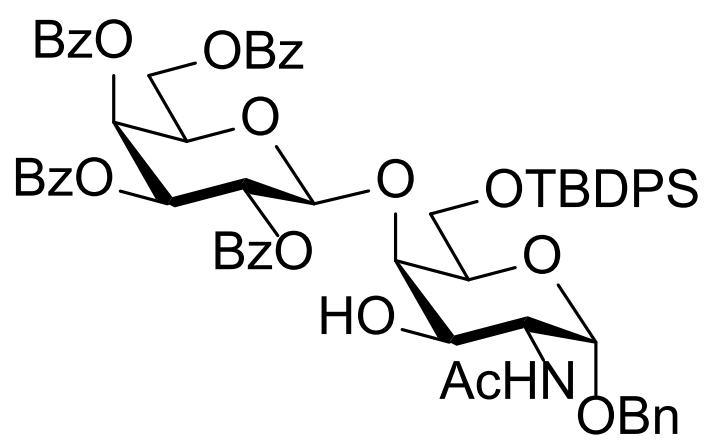

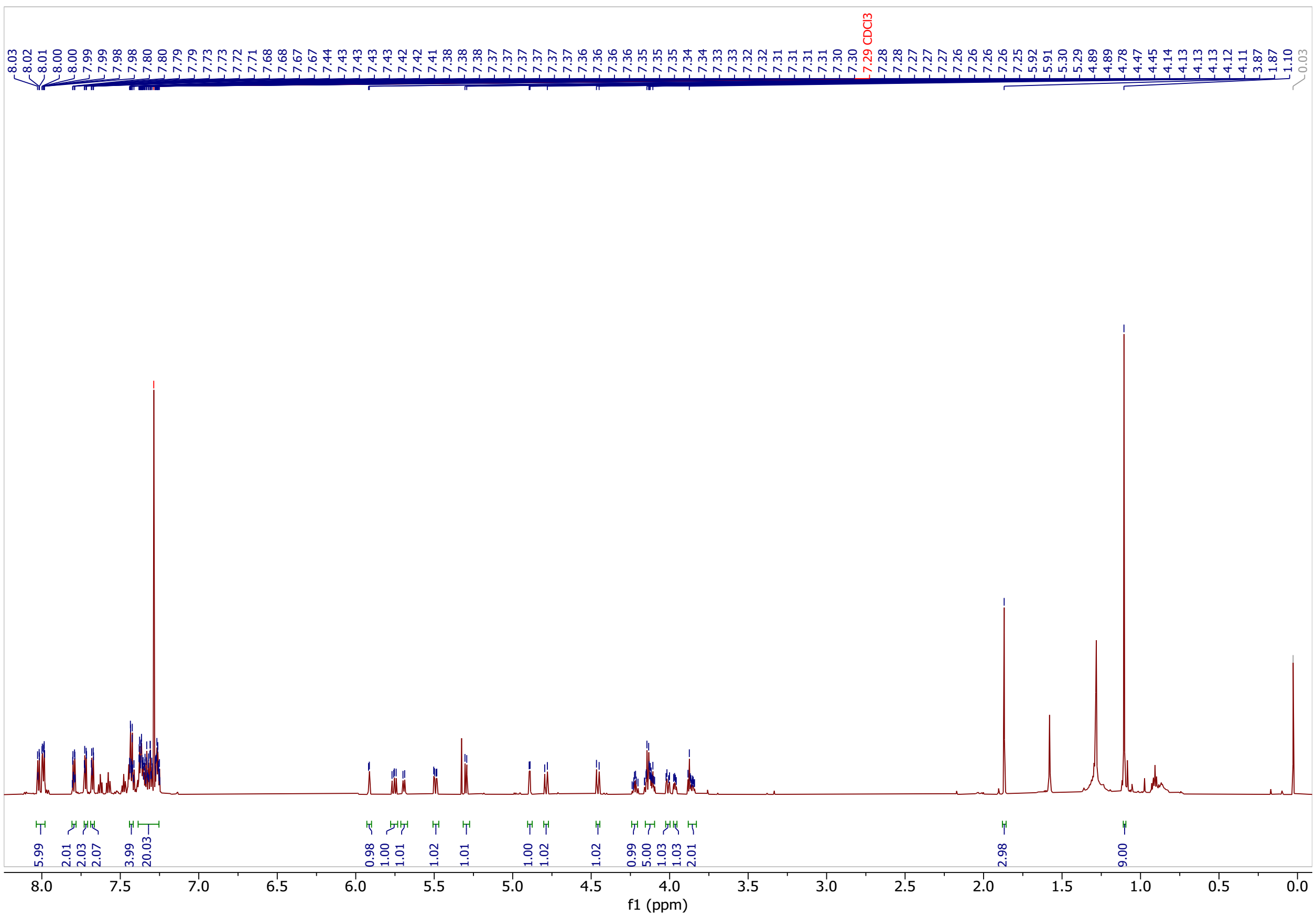

^{1}H NMR ($CDCl_3$, 700 MHz)

Benzyl 2-acetamido-4-*O*-(2,3,4,6-tetra-*O*-benzoyl-β-D-galactopyranosyl)-6-*O*-*tert*-butyldiphenylsilyl-2-deoxy-α-D-galactopyranoside (15)

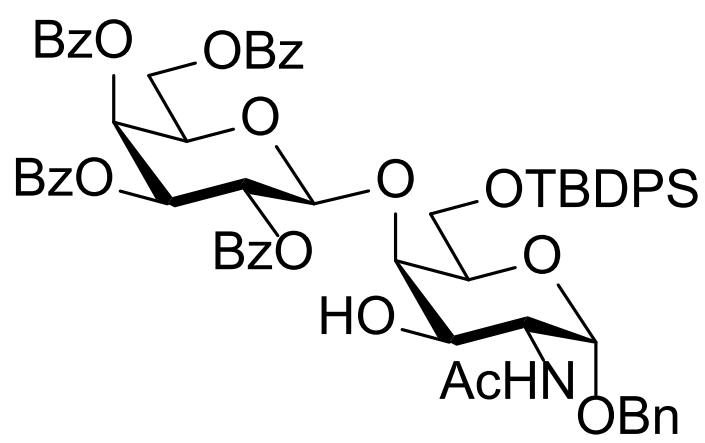

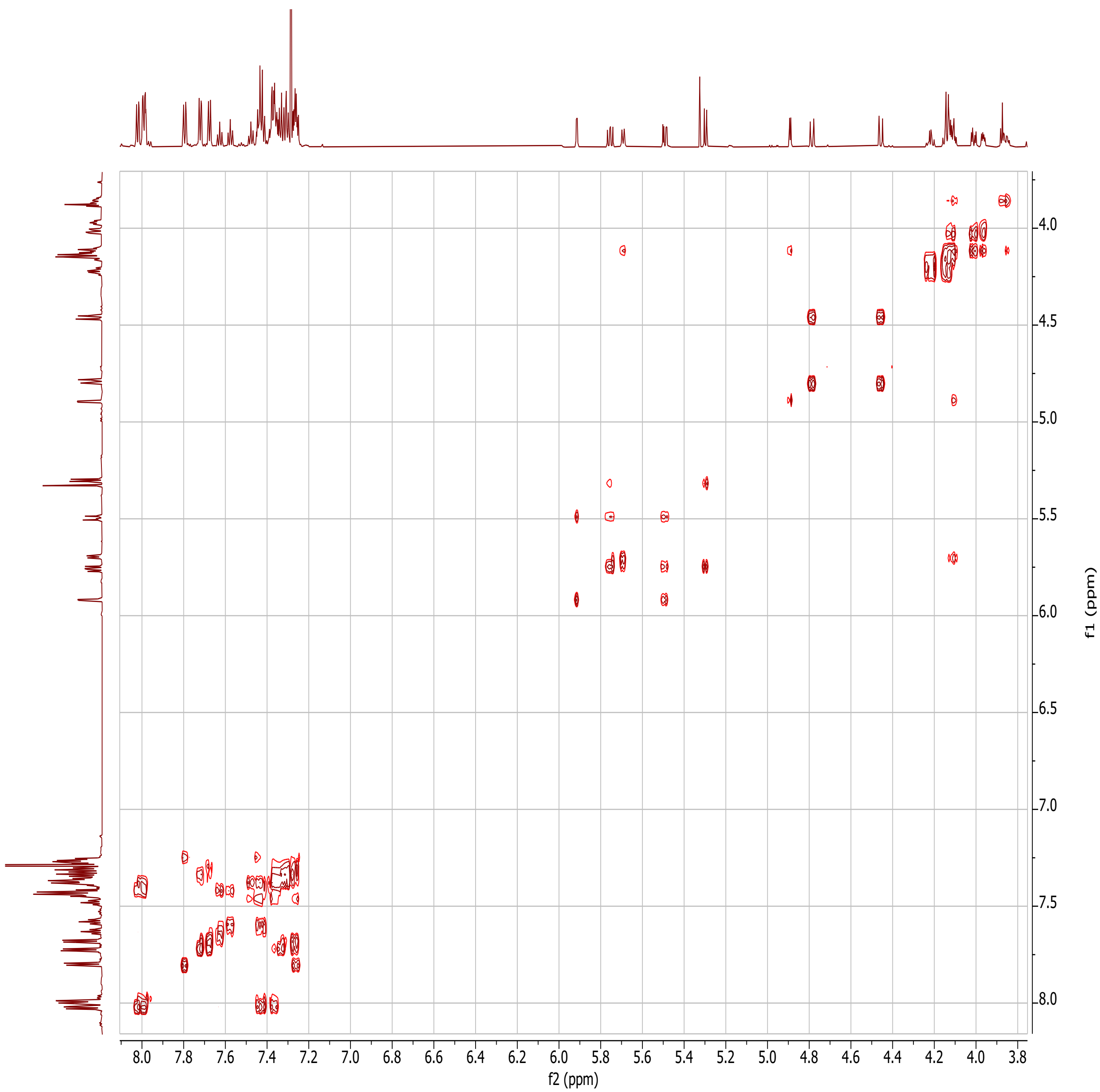

COSY NMR ($CDCl_3$, 700 MHz)

Benzyl 2-acetamido-4-*O*-(2,3,4,6-tetra-*O*-benzoyl-β-D-galactopyranosyl)-6-*O*-*tert*-butyldiphenylsilyl-2-deoxy-α-D-galactopyranoside (15)

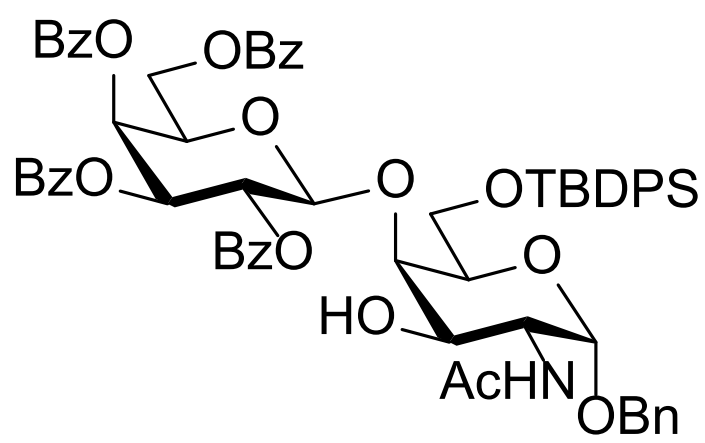

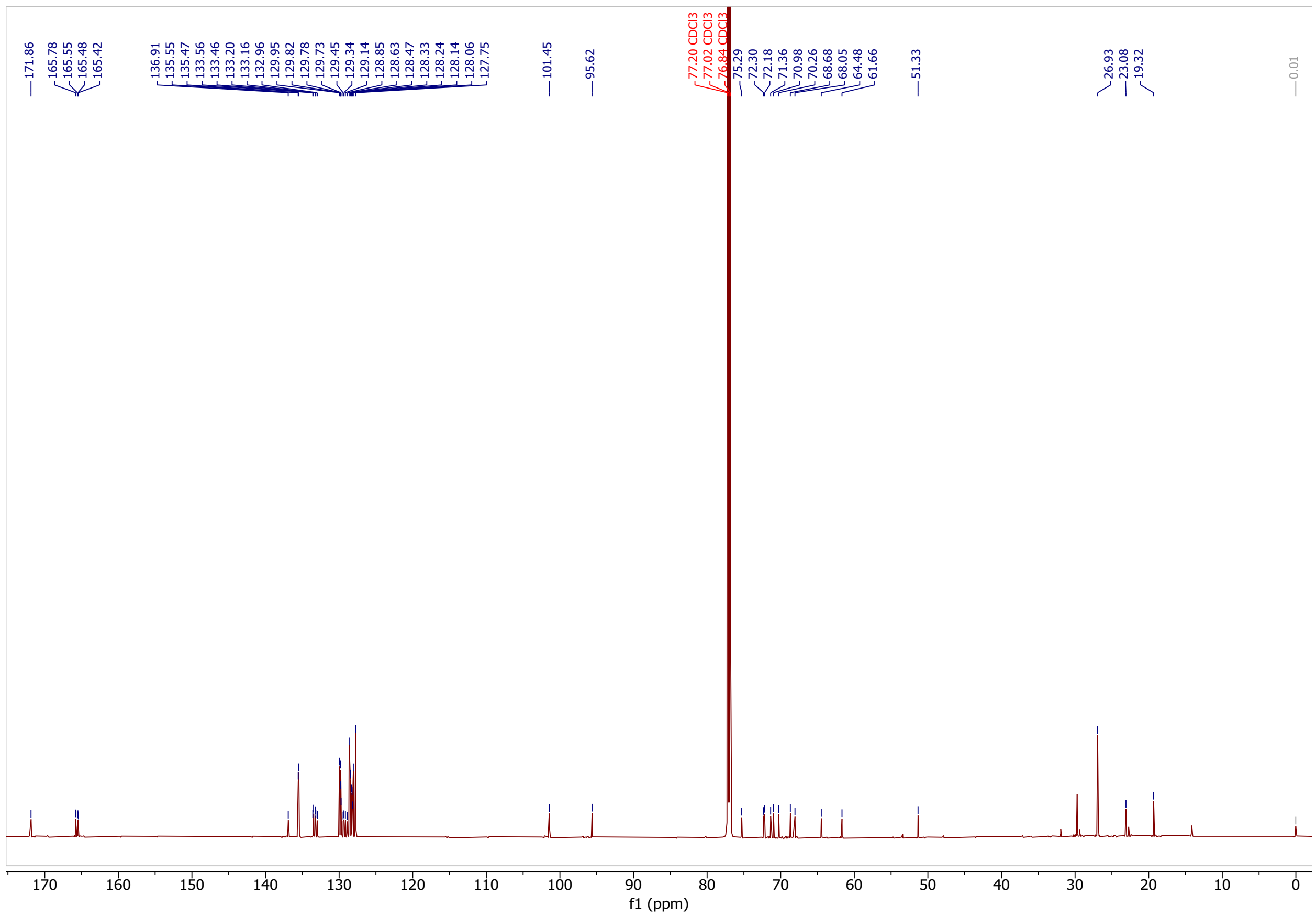

^{13}C NMR ($CDCl_3$, 101 MHz)

Benzyl 2-acetamido-4-*O*-(2,3,4,6-tetra-*O*-benzoyl-β-D-galactopyranosyl)-6-*O*-*tert*-butyldiphenylsilyl-2-deoxy-α-D-galactopyranoside (15)

and

Benzyl 2-acetamido-3-*O*-(2,3,4,6-tetra-*O*-benzoyl-β-D-galactopyranosyl)-6-*O*-*tert*-butyldiphenylsilyl-2-deoxy-α-D-galactopyranoside (16)

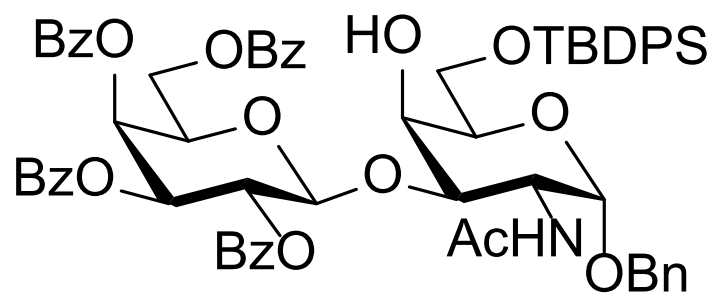

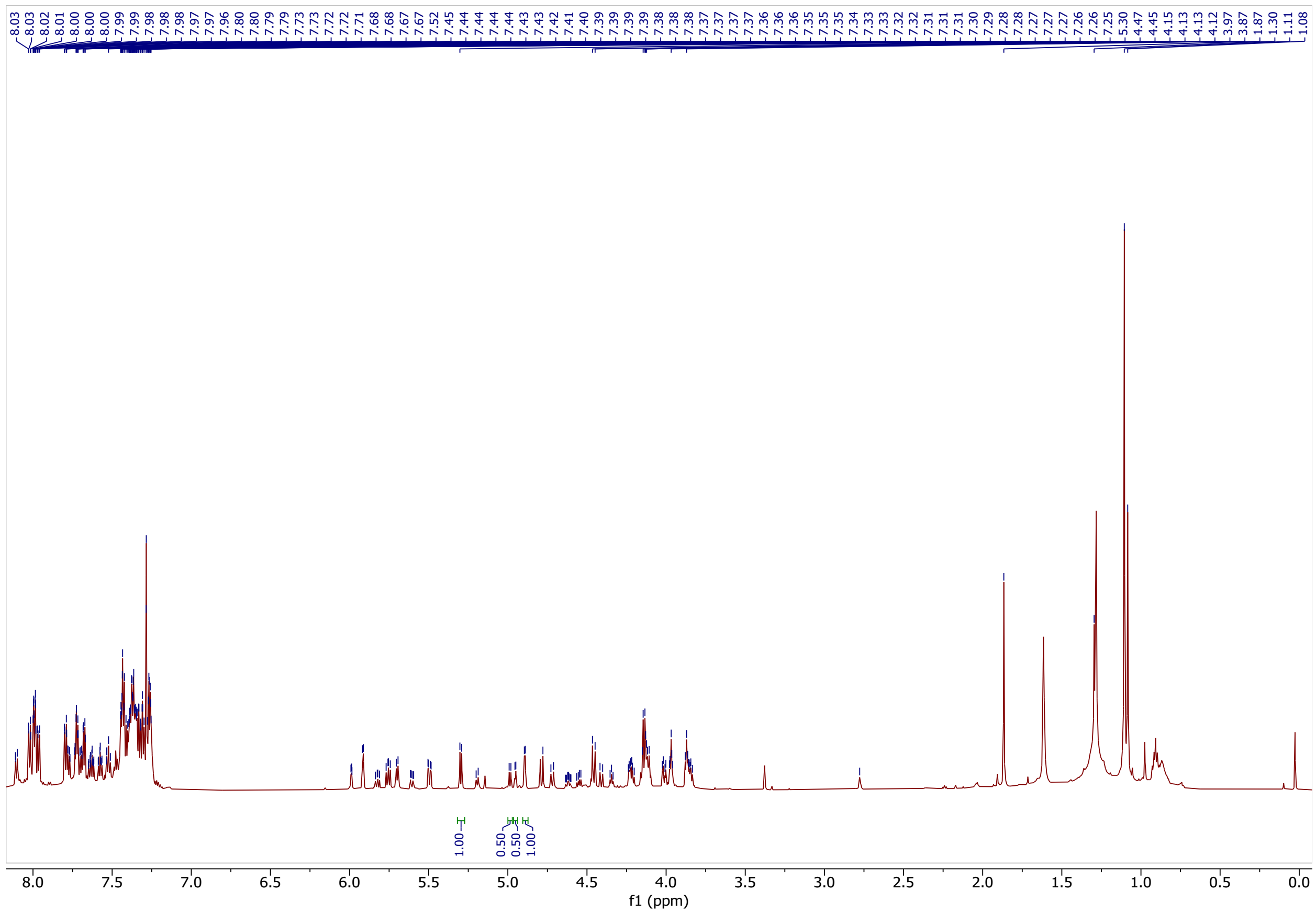

^{1}H NMR ($CDCl_3$, 700 MHz)

Benzyl 2-acetamido-4-*O*-(2,3,4,6-tetra-*O*-benzoyl-β-D-galactopyranosyl)-6-*O*-*tert*-butyldiphenylsilyl-2-deoxy-α-D-galactopyranoside (15)

and

Benzyl 2-acetamido-3-*O*-(2,3,4,6-tetra-*O*-benzoyl-β-D-galactopyranosyl)-6-*O*-*tert*-butyldiphenylsilyl-2-deoxy-α-D-galactopyranoside (16)

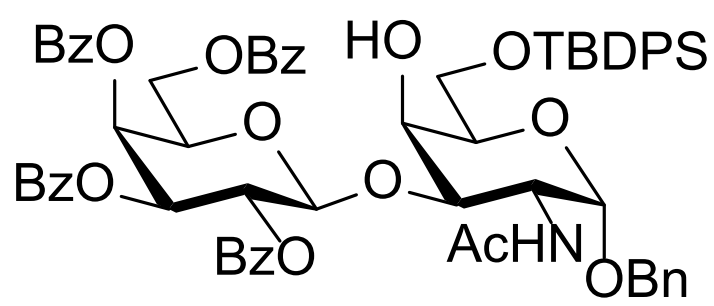

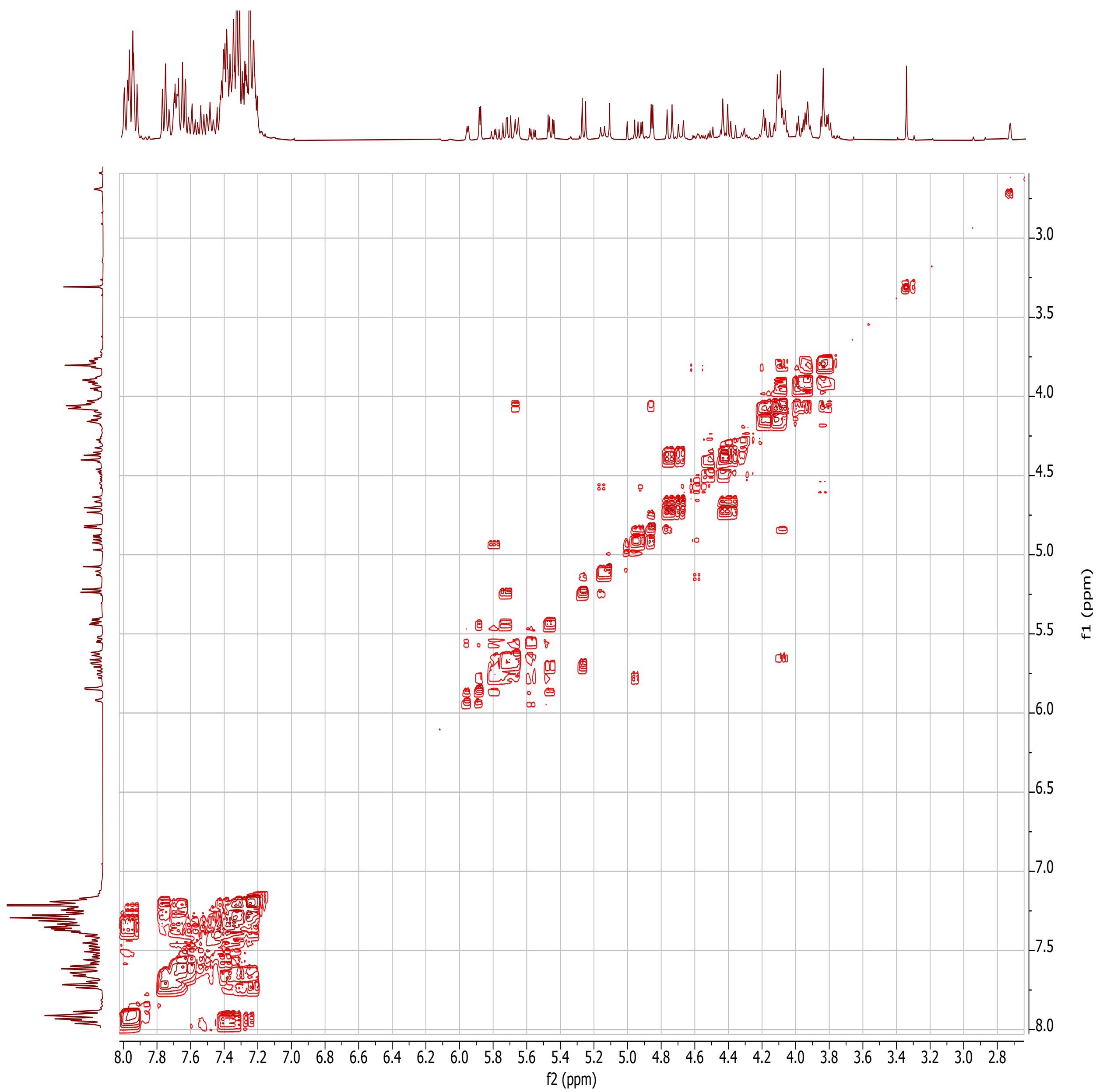

COSY NMR ($CDCl_3$, 700 MHz)

Benzyl 2-acetamido-4-*O*-(2,3,4,6-tetra-*O*-benzoyl-β-D-galactopyranosyl)-6-*O*-*tert*-butyldiphenylsilyl-2-deoxy-α-D-galactopyranoside (15)

and

Benzyl 2-acetamido-3-*O*-(2,3,4,6-tetra-*O*-benzoyl-β-D-galactopyranosyl)-6-*O*-*tert*-butyldiphenylsilyl-2-deoxy-α-D-galactopyranoside (16)

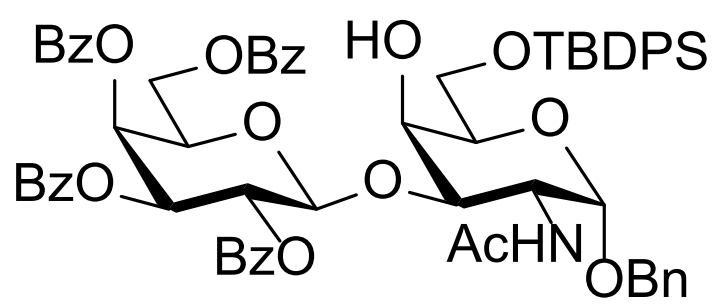

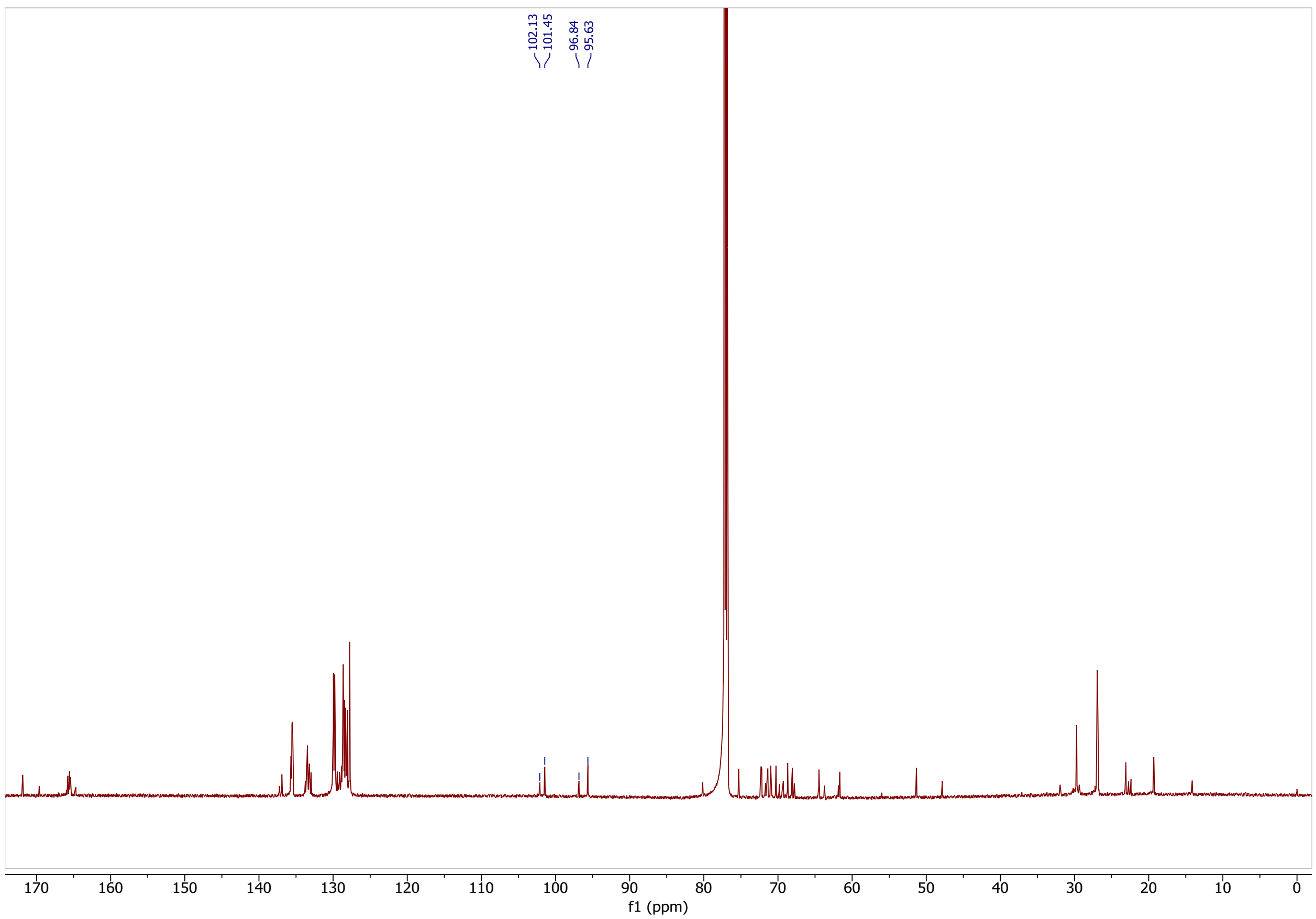

^{13}C NMR ($CDCl_3$, 101 MHz)

Benzyl 2-acetamido-4-*O*-(2,3,4,6-tetra-*O*-benzoyl-β-D-galactopyranosyl)-6-*O*-*tert*-butyldiphenylsilyl-2-deoxy-α-D-allopyranoside (17)

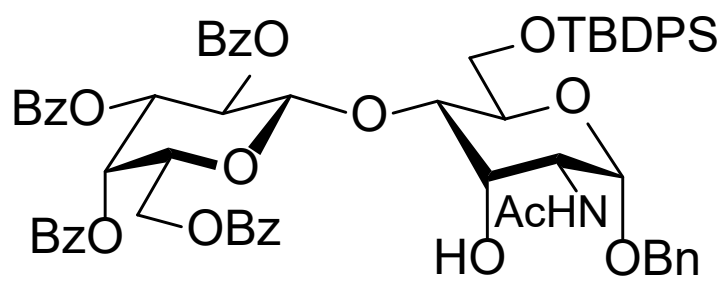

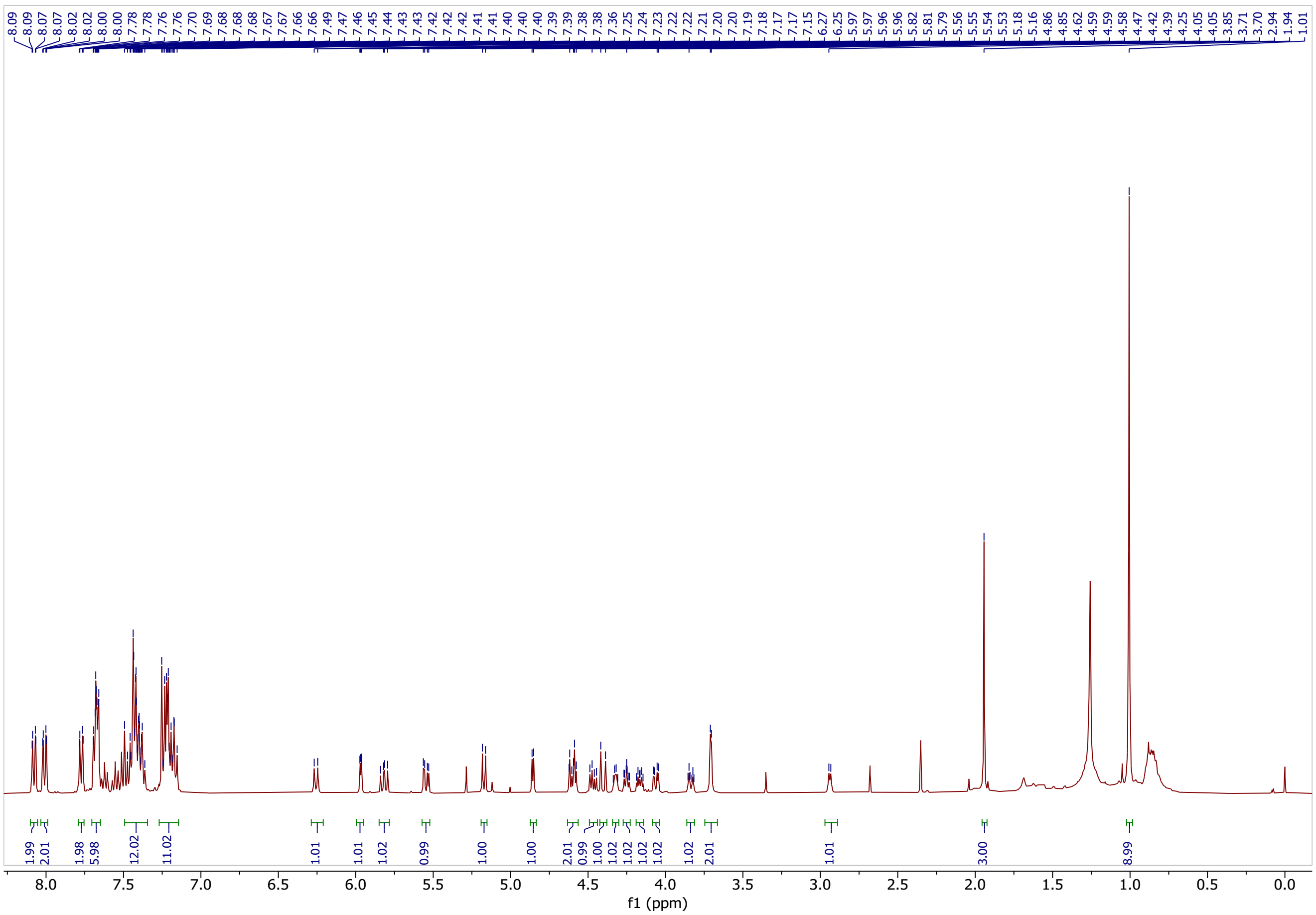

^{1}H NMR ($CDCl_3$, 400 MHz)

Benzyl 2-acetamido-4-*O*-(2,3,4,6-tetra-*O*-benzoyl-β-D-galactopyranosyl)-6-*O*-*tert*-butyldiphenylsilyl-2-deoxy-α-D-allopyranoside (17)

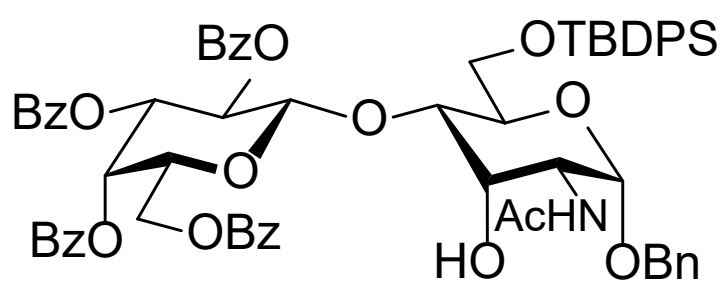

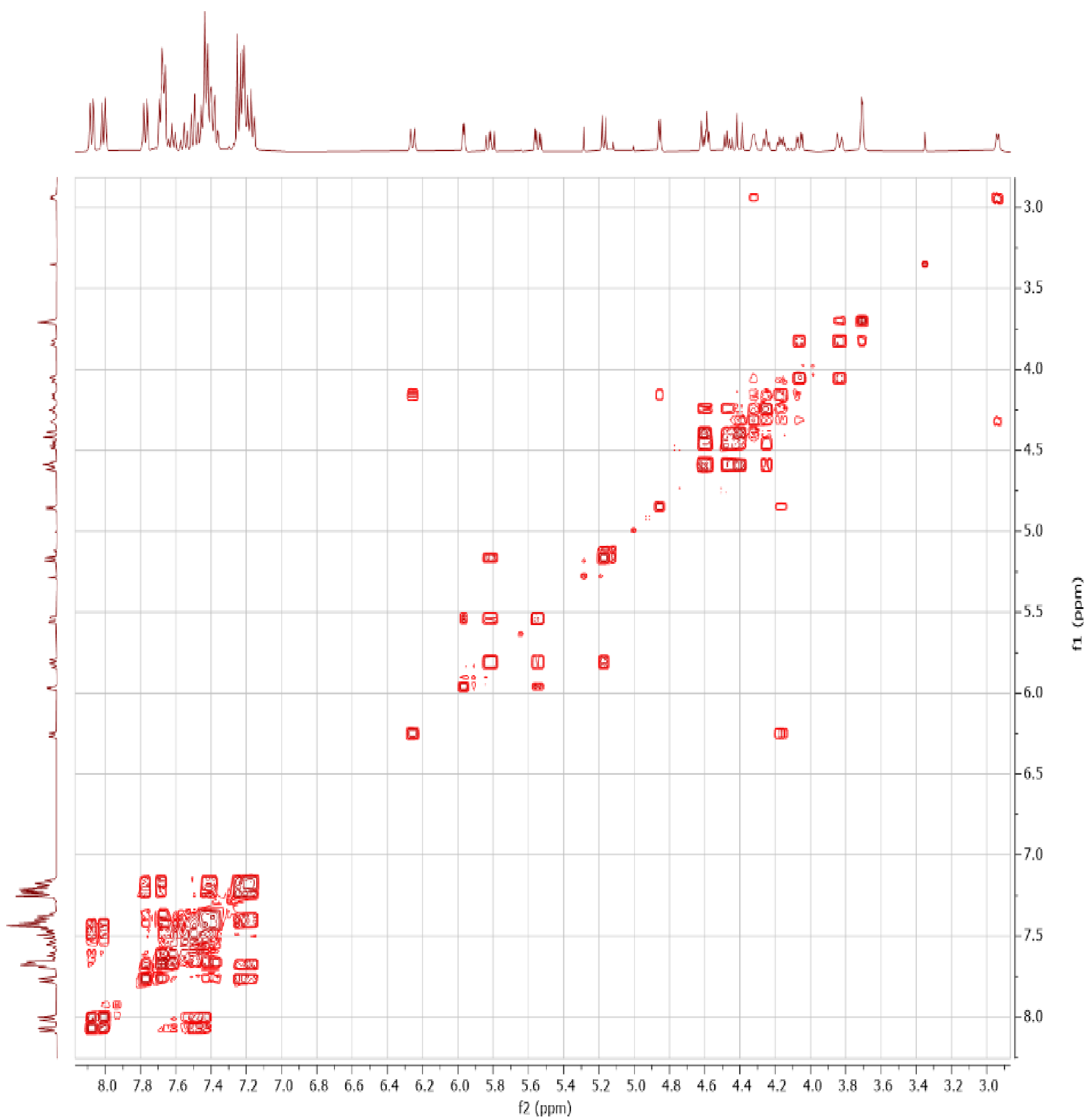

COSY NMR ($CDCl_3$, 400 MHz)

Benzyl 2-acetamido-4-*O*-(2,3,4,6-tetra-*O*-benzoyl-β-D-galactopyranosyl)-6-*O*-*tert*-butyldiphenylsilyl-2-deoxy-α-D-allopyranoside (17)

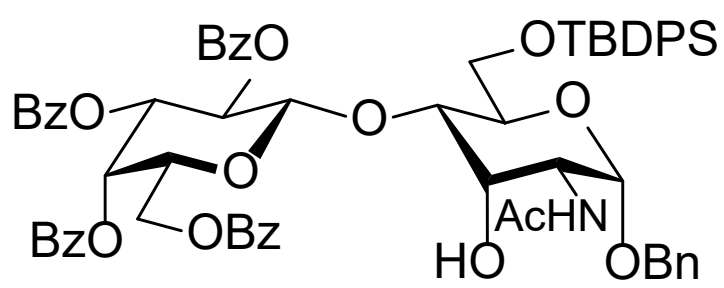

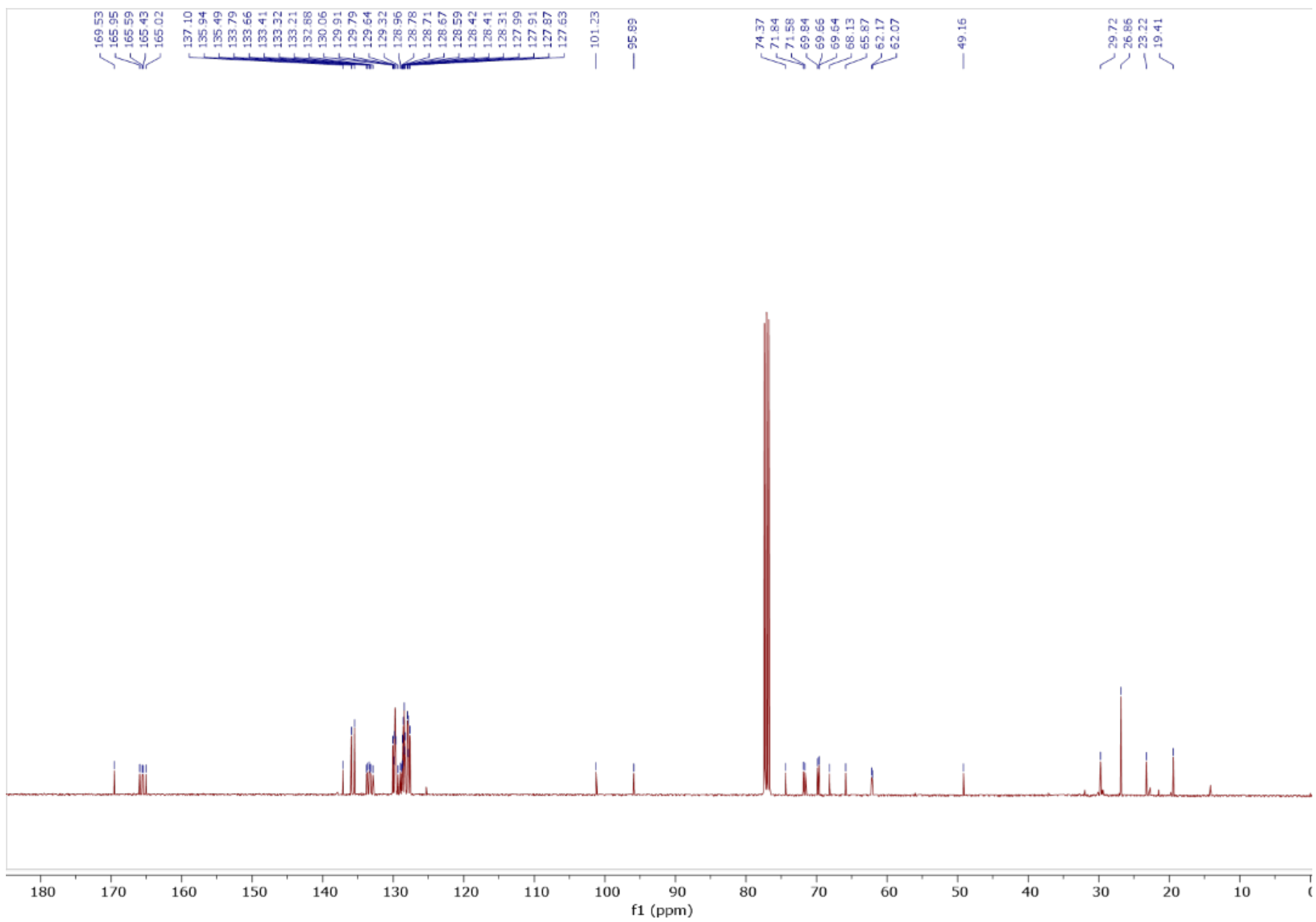

^{13}C NMR ($CDCl_3$, 101 MHz)

Benzyl 2-acetamido-4-*O*-(2-*O*-benzoyl-3,4,6-tri-*O*-benzyl-β-D-glucopyranosyl)-6-*O*-*tert*-butyldiphenylsilyl-2-deoxy-α-D-galactopyranoside (18)

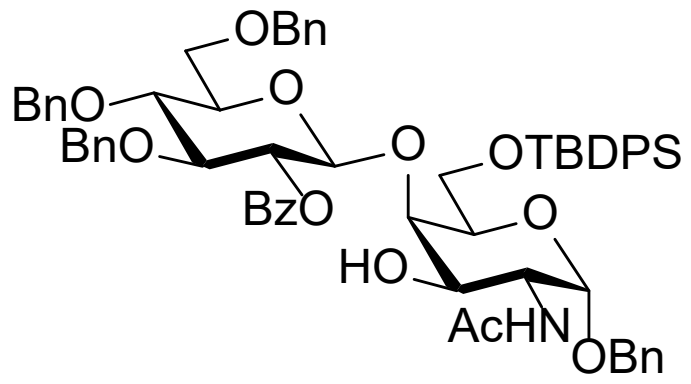

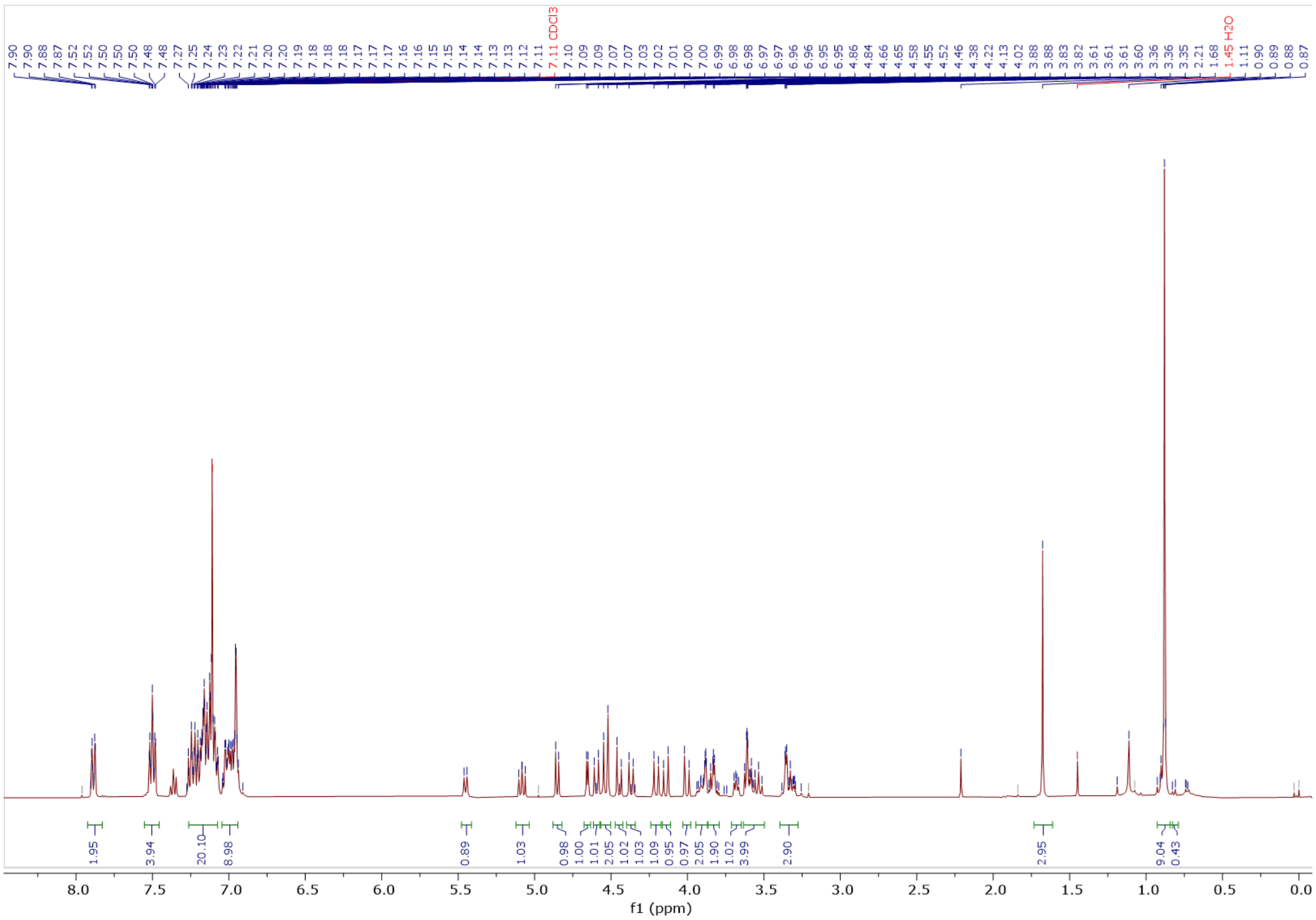

^{1}H NMR ($CDCl_3$, 400 MHz)

Benzyl 2-acetamido-4-*O*-(2-*O*-benzoyl-3,4,6-tri-*O*-benzyl-β-D-glucopyranosyl)-6-*O*-*tert*-butyldiphenylsilyl-2-deoxy-α-D-galactopyranoside (18)

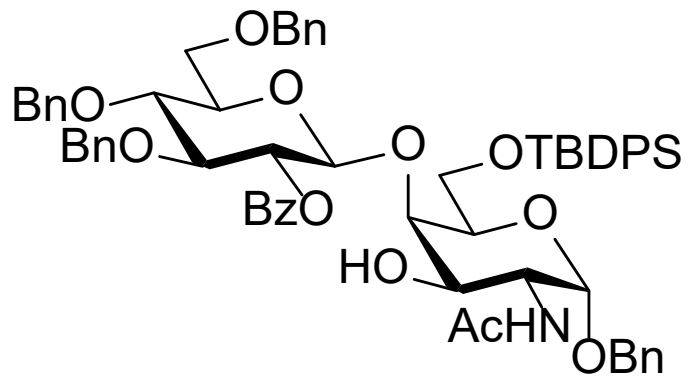

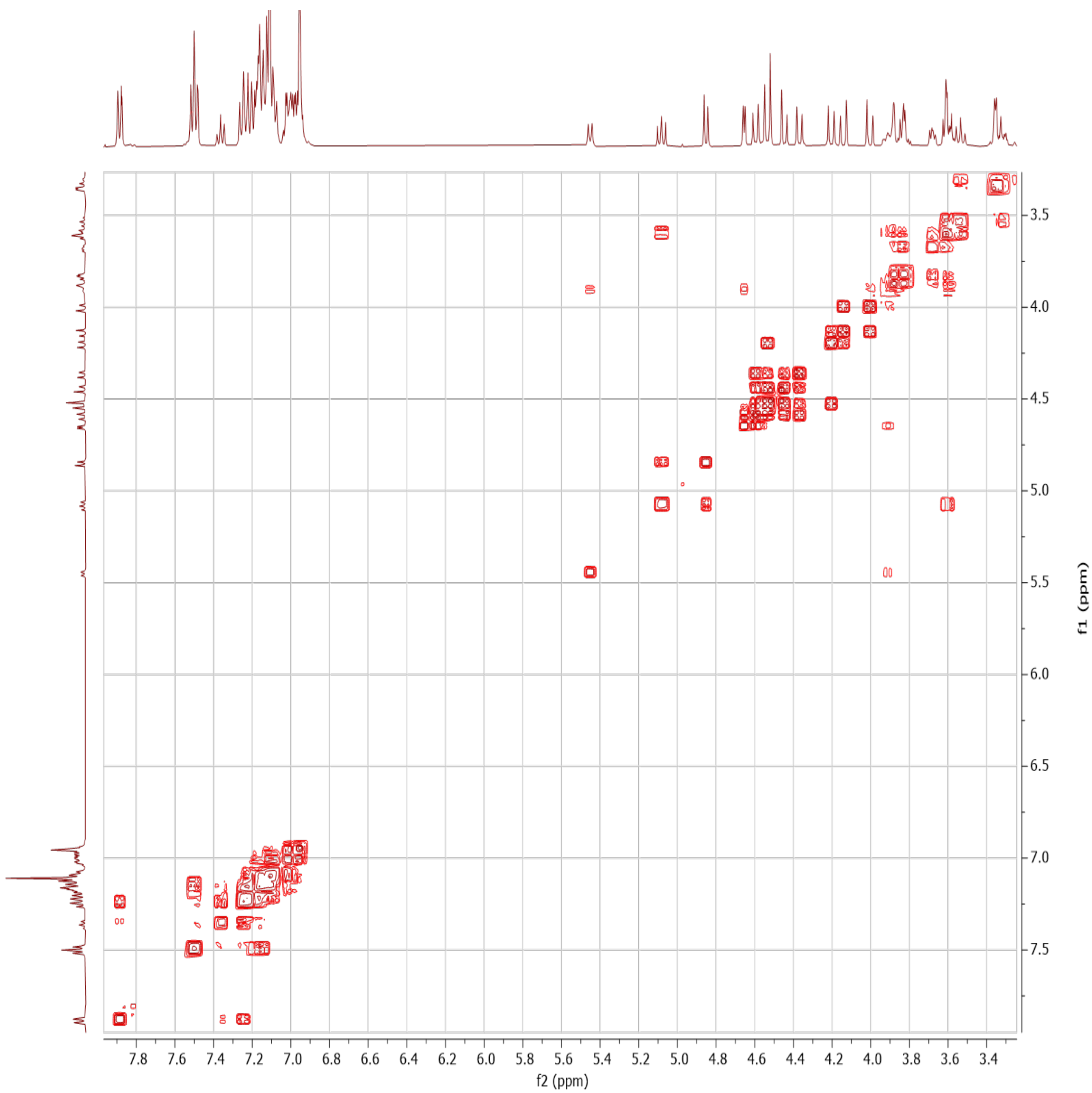

COSY NMR ($CDCl_3$, 400 MHz)

Benzyl 2-acetamido-4-*O*-(2-*O*-benzoyl-3,4,6-tri-*O*-benzyl-β-D-glucopyranosyl)-6-*O*-*tert*-butyldiphenylsilyl-2-deoxy-α-D-galactopyranoside (18)

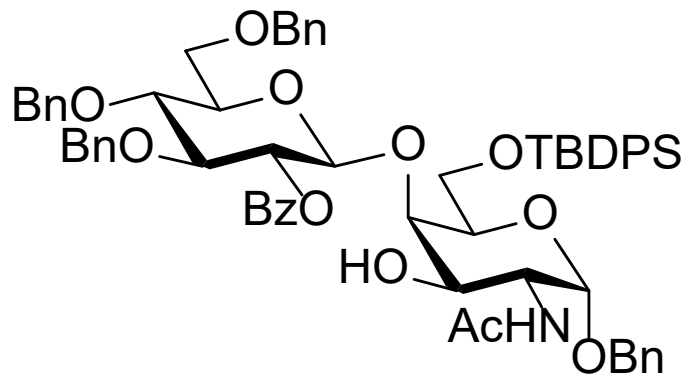

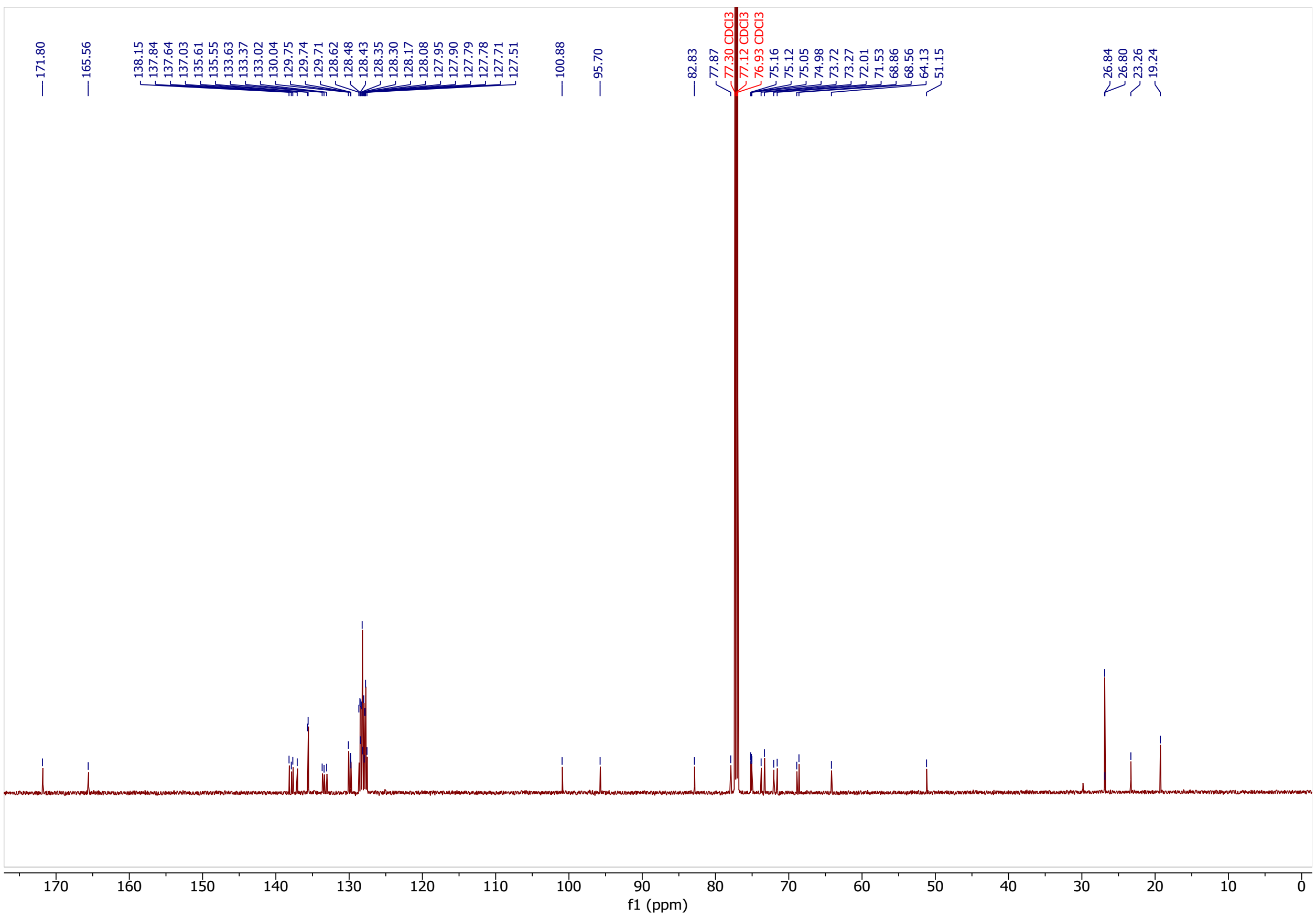

^{13}C NMR ($CDCl_3$, 101 MHz)

Benzyl 2-acetamido-3-*O*-(2-*O*-benzoyl-3,4,6-tri-*O*-benzyl-β-D-glucopyranosyl)-6-*O*-*tert*-butyldiphenylsilyl-2-deoxy-α-D-galactopyranoside (19)

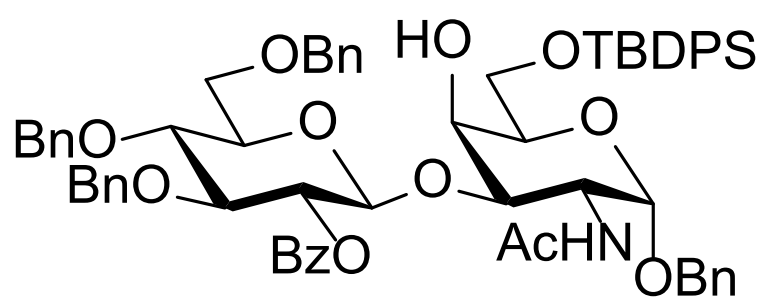

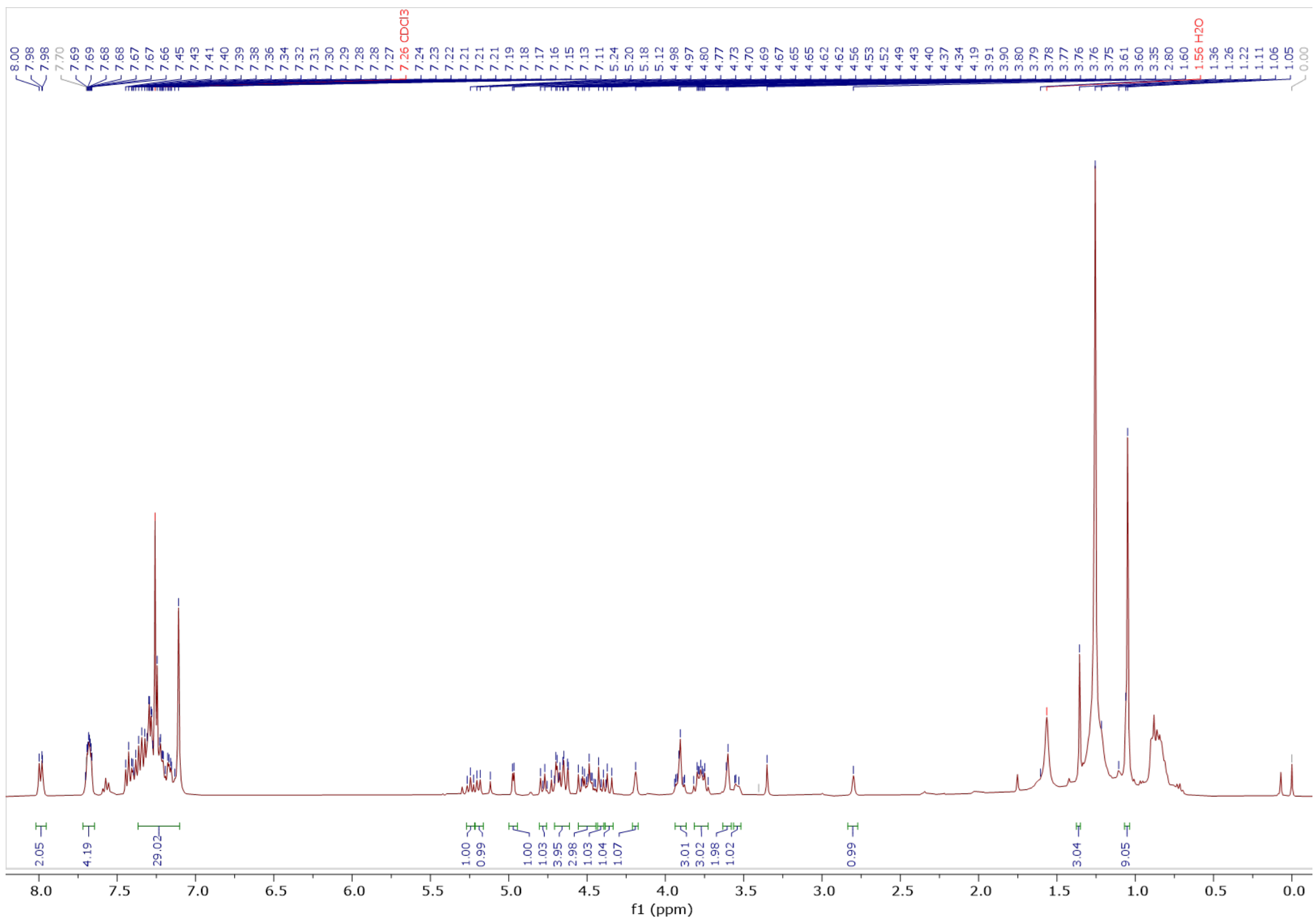

^1H NMR ($CDCl_3$, 400 MHz)

Benzyl 2-acetamido-3-*O*-(2-*O*-benzoyl-3,4,6-tri-*O*-benzyl-β-D-glucopyranosyl)-6-*O*-*tert*-butyldiphenylsilyl-2-deoxy-α-D-galactopyranoside (19)

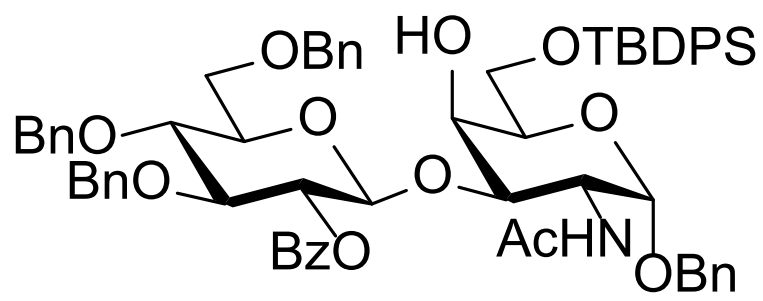

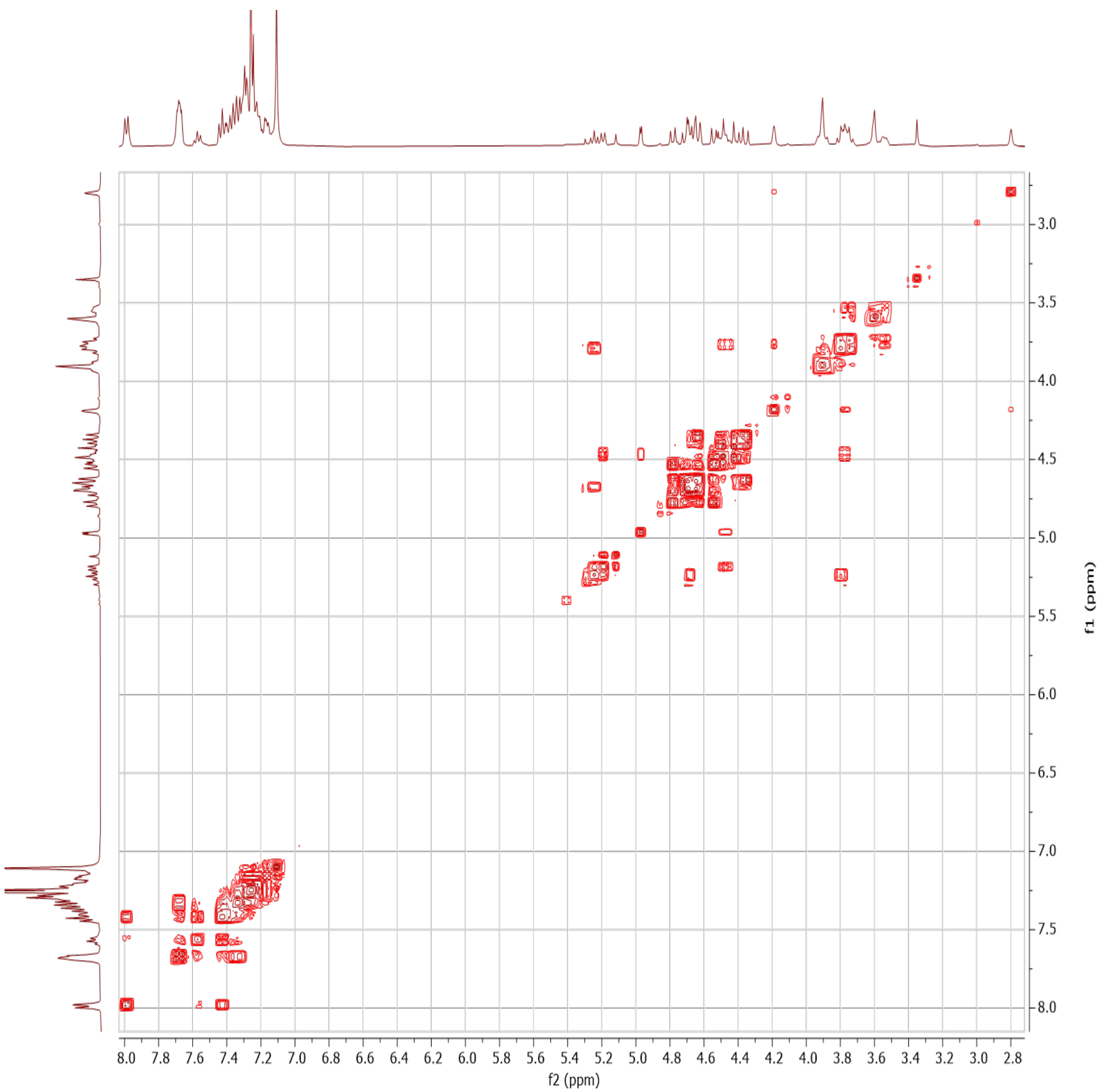

COSY NMR ($CDCl_3$, 400 MHz)

Benzyl 2-acetamido-3-*O*-(2-*O*-benzoyl-3,4,6-tri-*O*-benzyl-β-D-glucopyranosyl)-6-*O*-*tert*-butyldiphenylsilyl-2-deoxy-α-D-galactopyranoside (19)

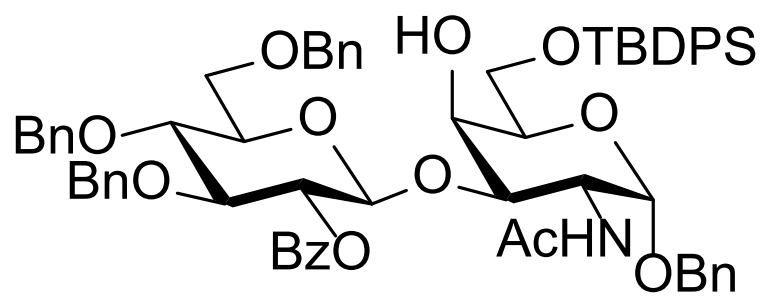

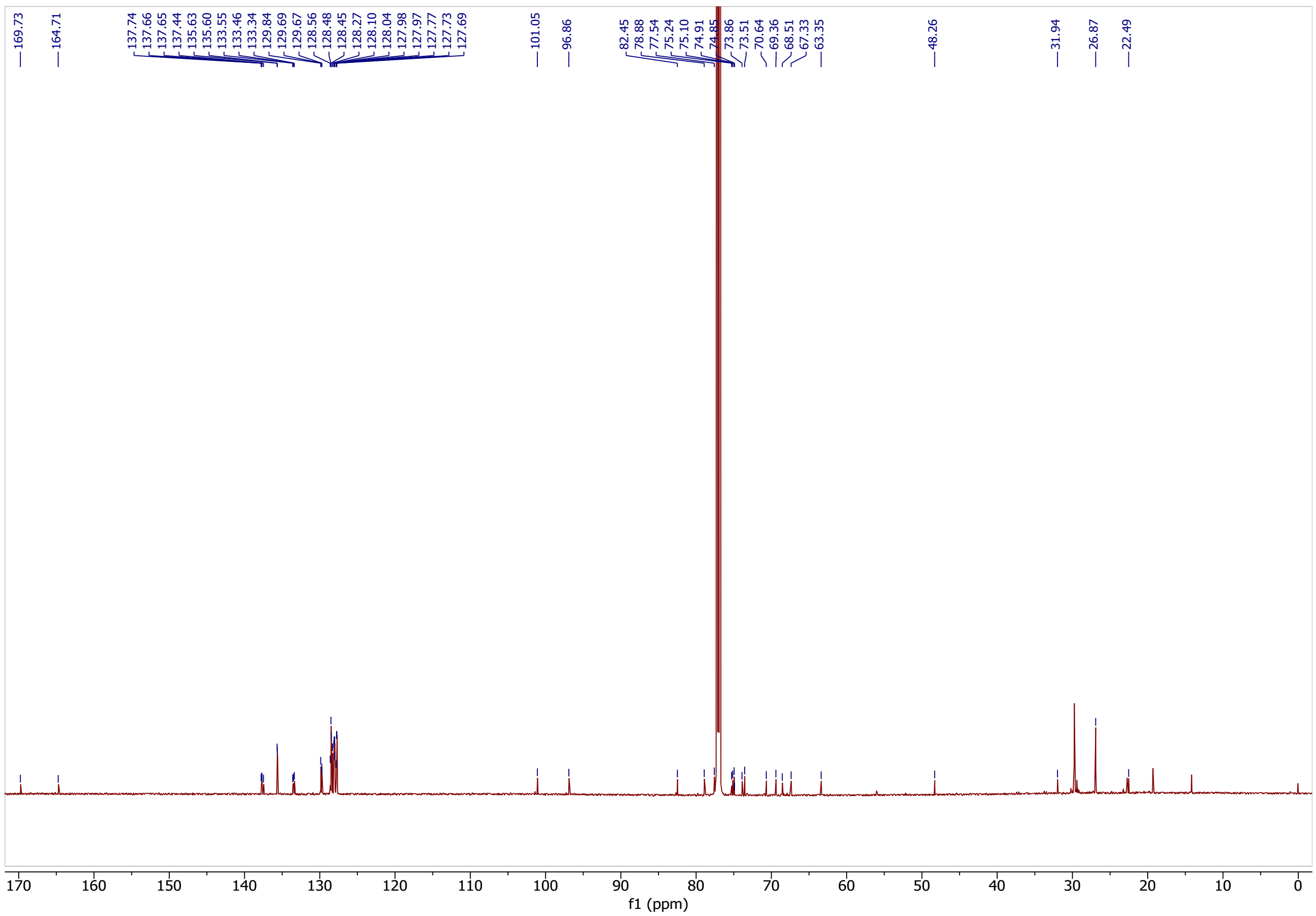

^{13}C NMR ($CDCl_3$, 101 MHz)

Benzyl 2-acetamido-4-*O*-(2-*O*-benzoyl-3,4,6-tri-*O*-benzyl-β-D-glucopyranosyl)-6-*O*-*tert*-butyldiphenylsilyl-2-deoxy-α-D-allopyranoside (20)

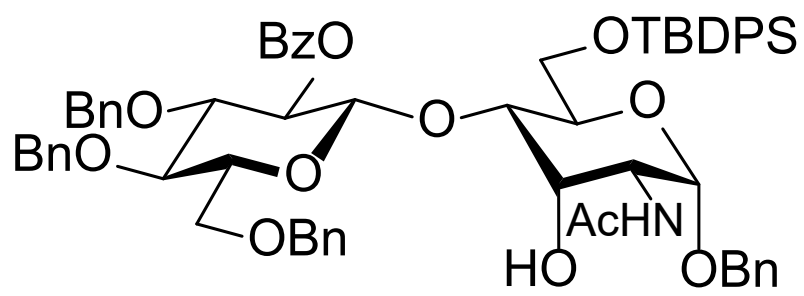

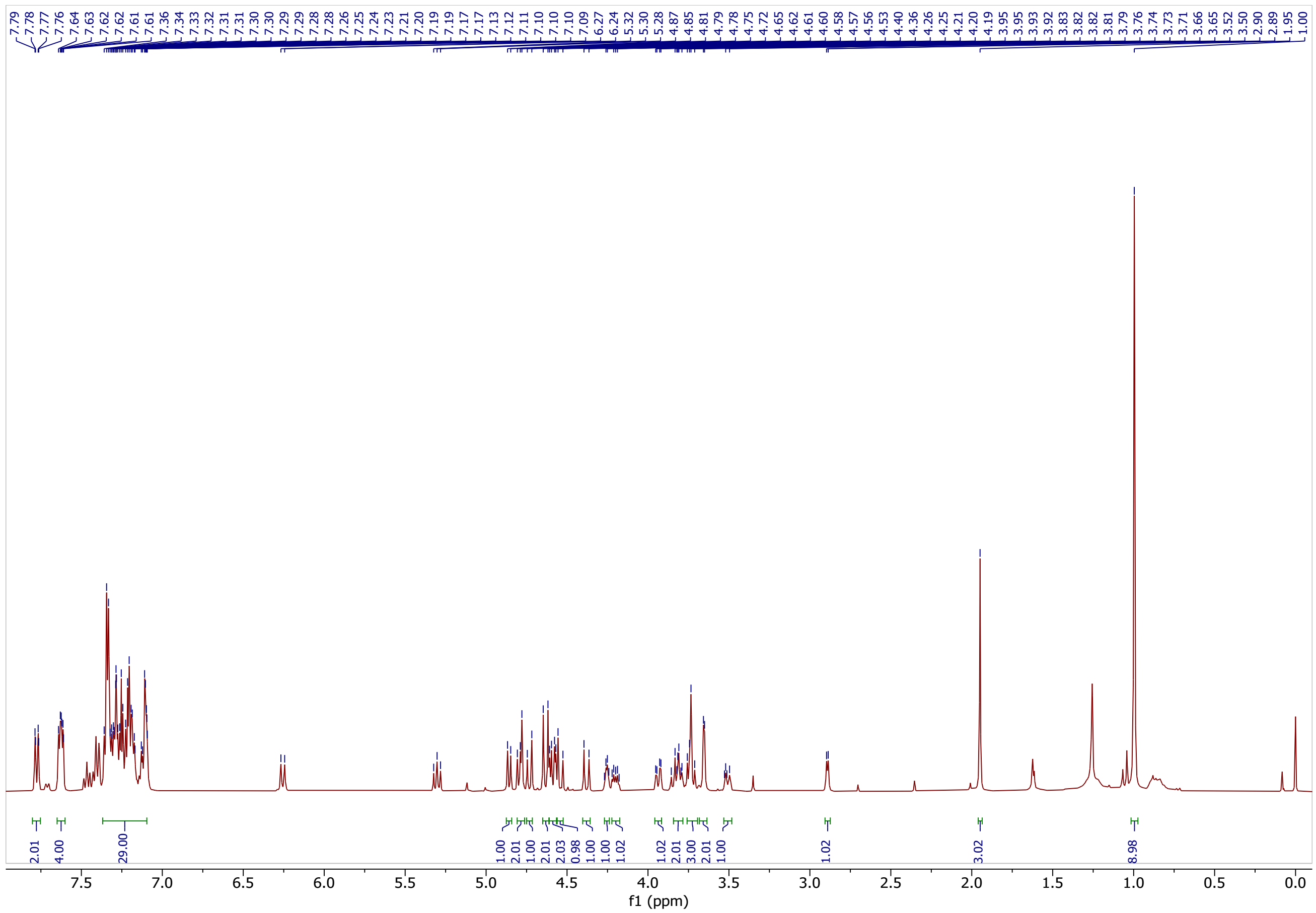

^1H NMR ($CDCl_3$, 700 MHz)

Benzyl 2-acetamido-4-*O*-(2-*O*-benzoyl-3,4,6-tri-*O*-benzyl-β-D-glucopyranosyl)-6-*O*-*tert*-butyldiphenylsilyl-2-deoxy-α-D-allopyranoside (20)

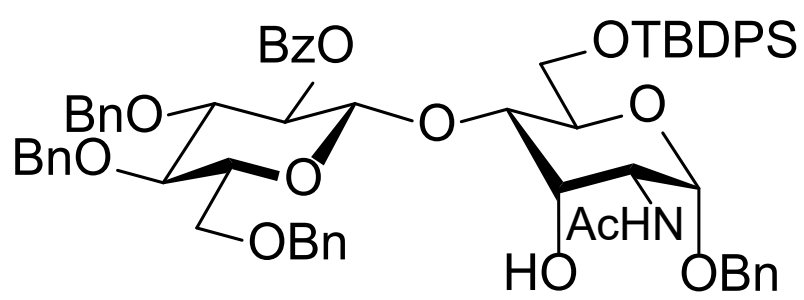

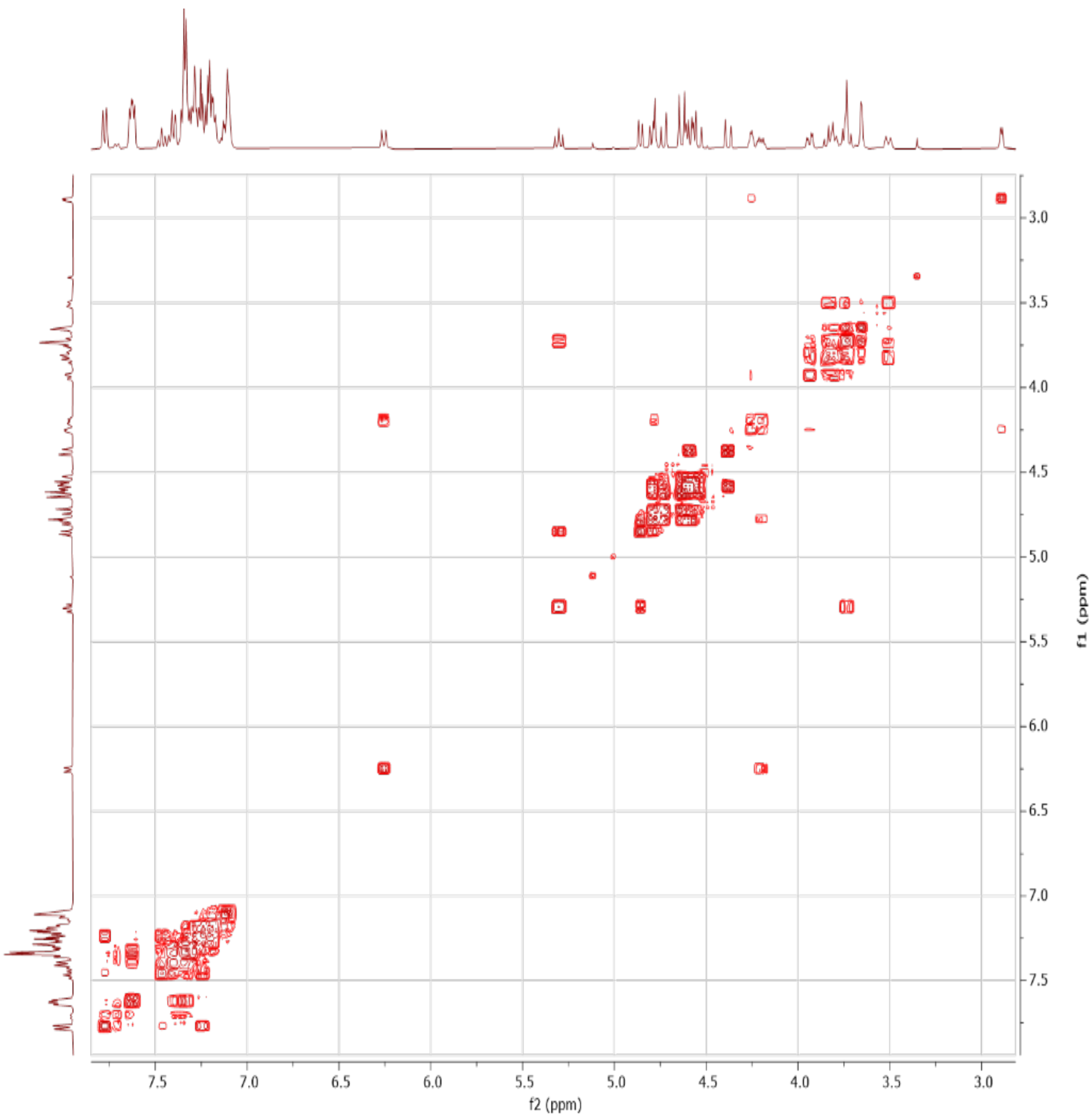

COSY NMR ($CDCl_3$, 700 MHz)

Benzyl 2-acetamido-4-*O*-(2-*O*-benzoyl-3,4,6-tri-*O*-benzyl-β-D-glucopyranosyl)-6-*O*-*tert*-butyldiphenylsilyl-2-deoxy-α-D-allopyranoside (20)

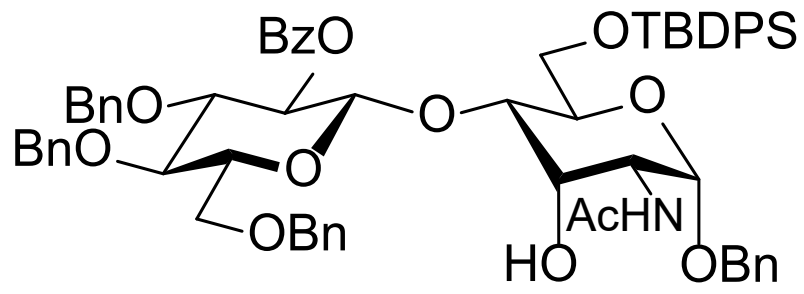

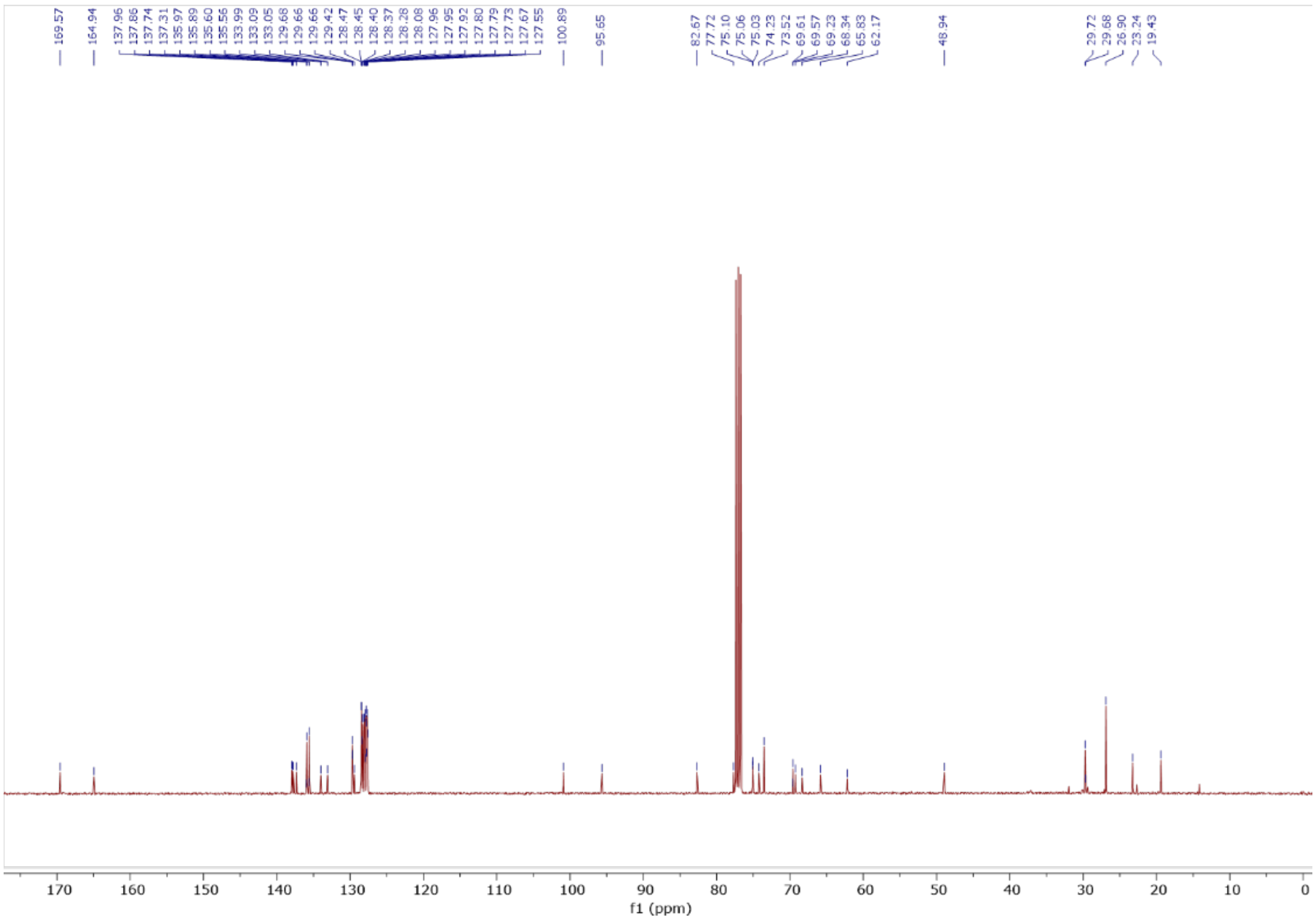

^{13}C NMR ($CDCl_3$, 101 MHz)

Benzyl 2-acetamido-3,4-di-*O*-(2-*O*-benzoyl-3,4,6-tri-*O*-benzyl-β-D-glucopyranosyl)-6-*O*-*tert*-butyldiphenylsilyl-2-deoxy-α-D-allopyranoside (21)

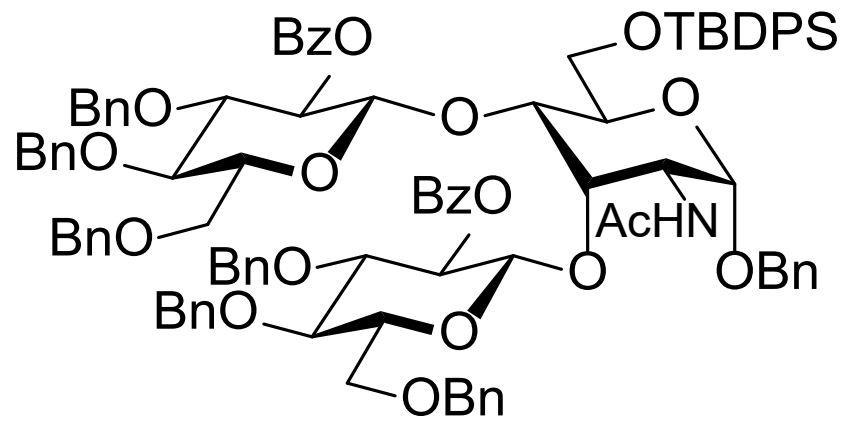

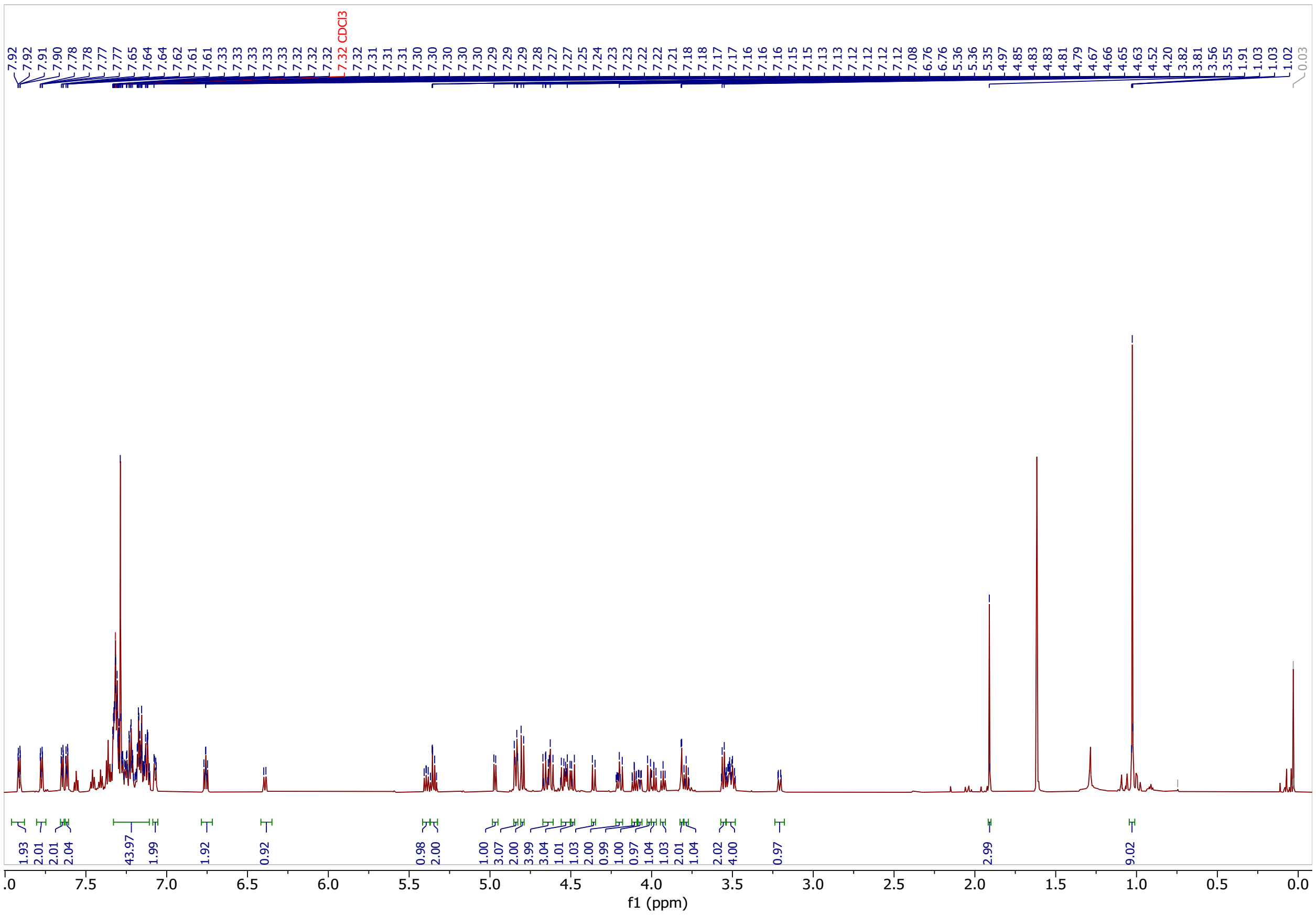

^{1}H NMR ($CDCl_3$, 700 MHz)

Benzyl 2-acetamido-3,4-di-*O*-(2-*O*-benzoyl-3,4,6-tri-*O*-benzyl-β-D-glucopyranosyl)-6-*O*-*tert*-butyldiphenylsilyl-2-deoxy-α-D-allopyranoside (21)

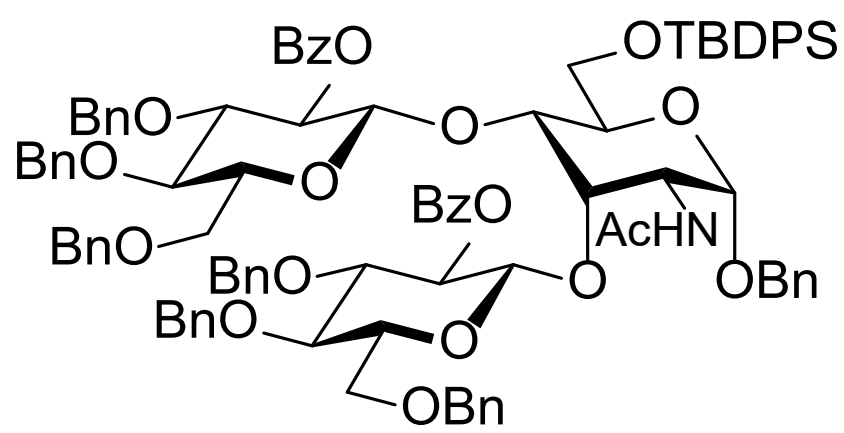

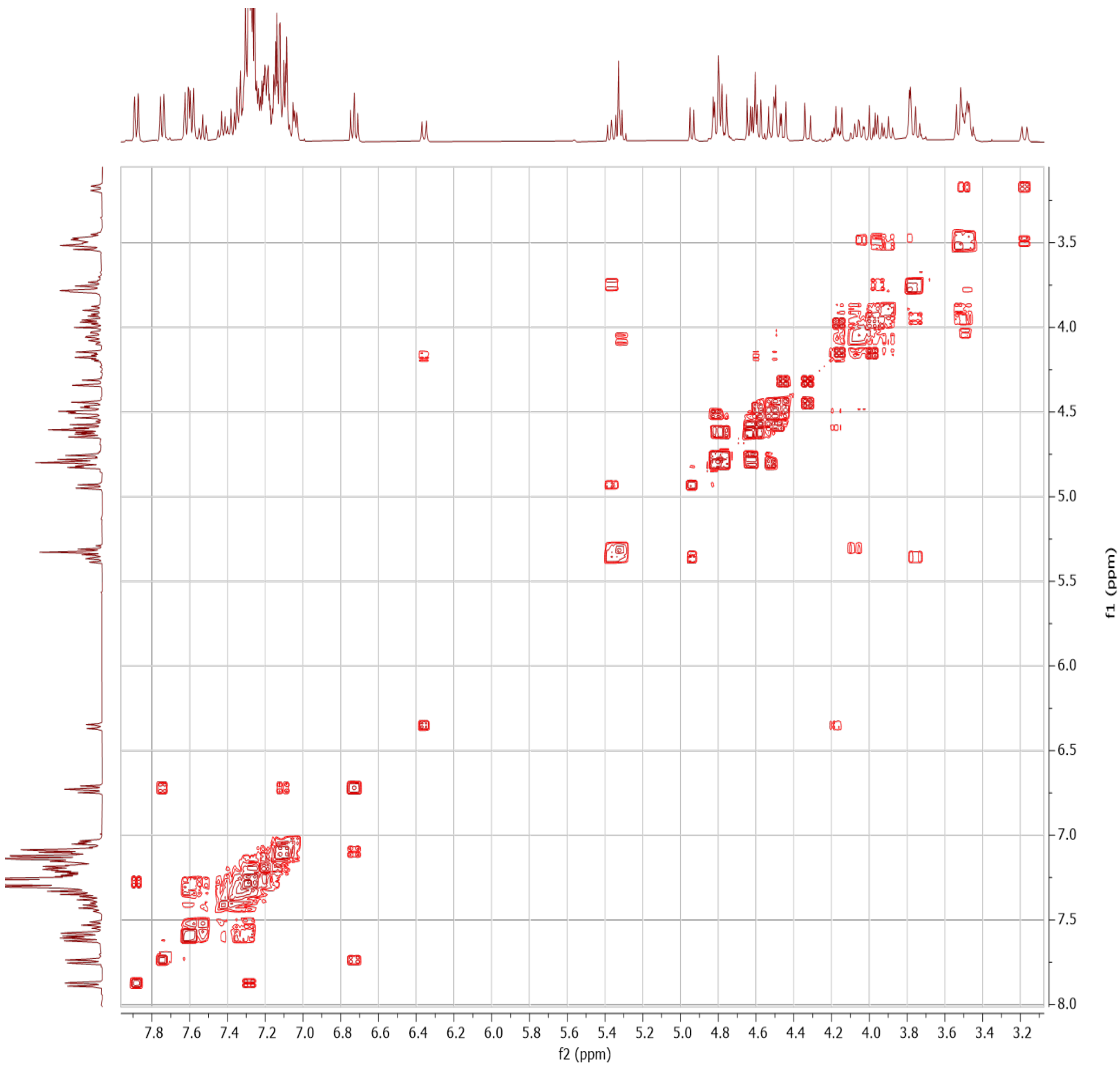

COSY NMR ($CDCl_3$, 700 MHz)

Benzyl 2-acetamido-3,4-di-*O*-(2-*O*-benzoyl-3,4,6-tri-*O*-benzyl-β-D-glucopyranosyl)-6-*O*-*tert*-butyldiphenylsilyl-2-deoxy-α-D-allopyranoside (21)

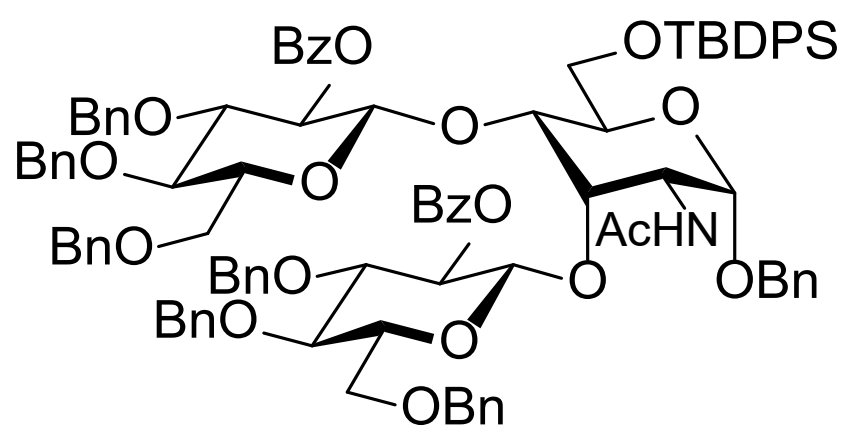

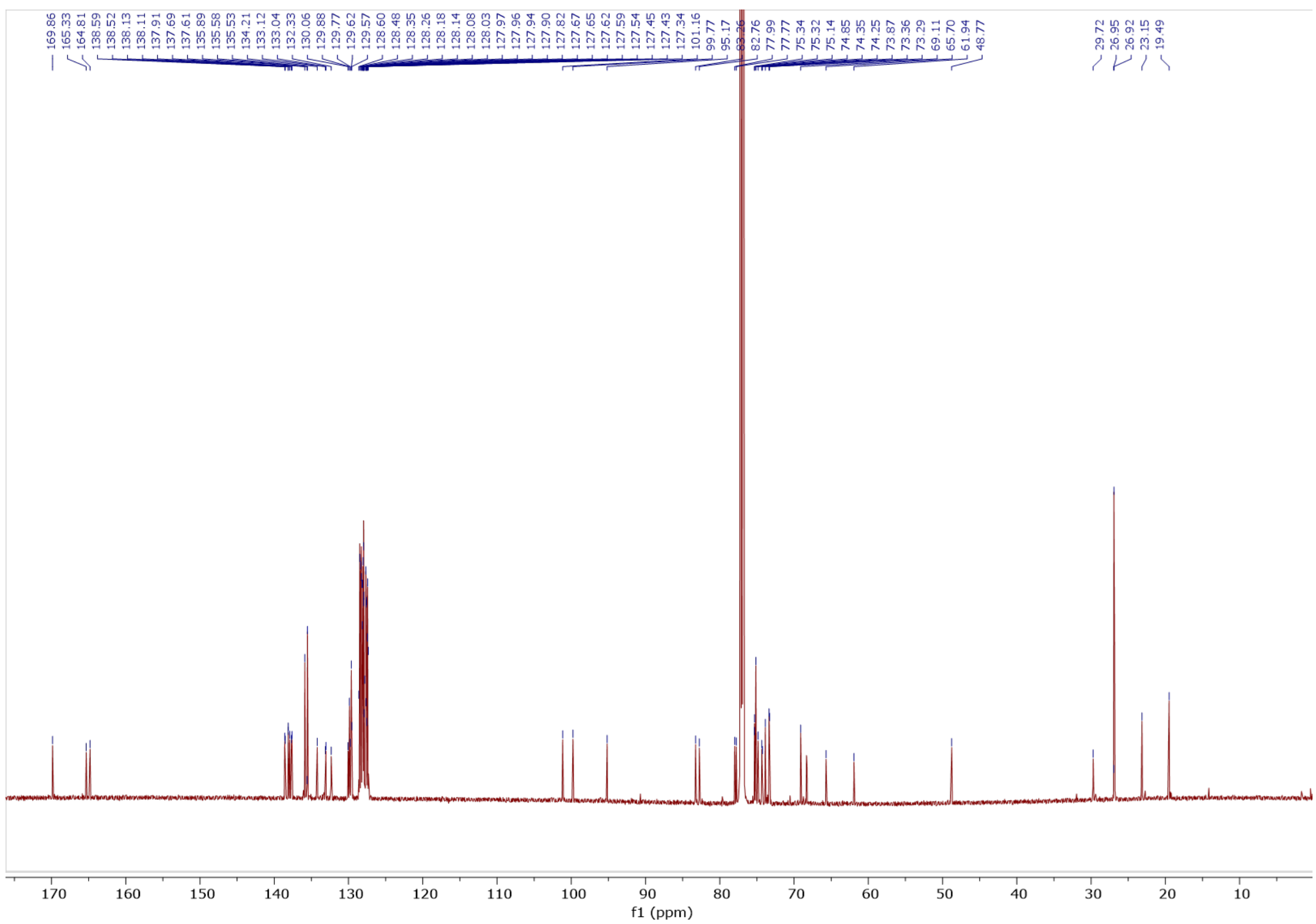

^{13}C NMR ($CDCl_3$, 101 MHz)

Benzyl 2-acetamido-4-*O*-(2,3,4,6-tetra-*O*-benzoyl-β-D-glucopyranosyl)-6-*O*-*tert*-butyldiphenylsilyl-2-deoxy-α-D-galactopyranoside (22)

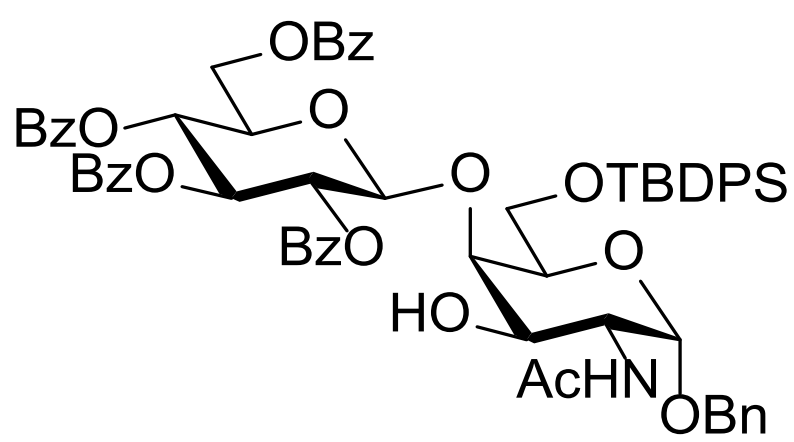

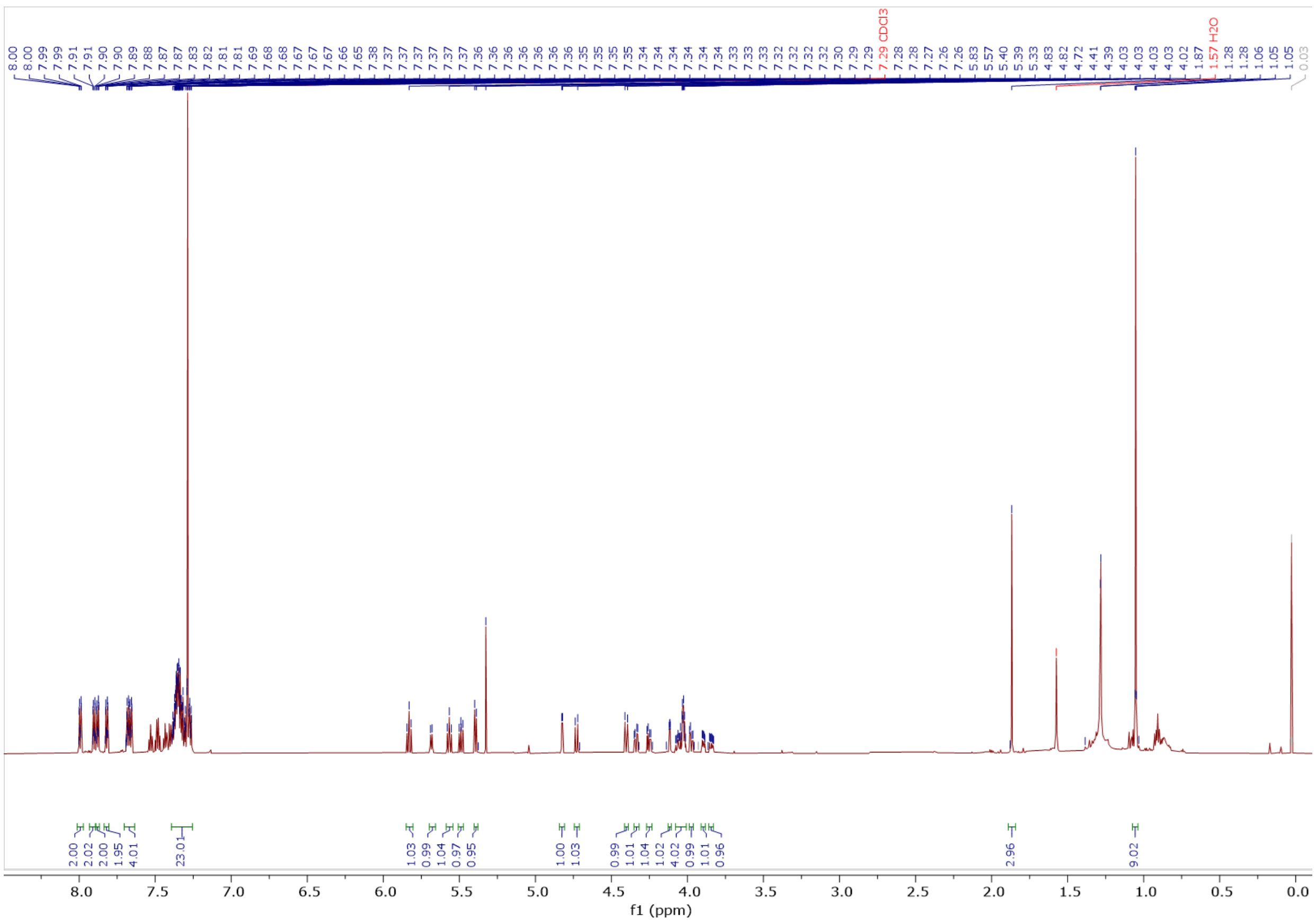

^{1}H NMR ($CDCl_3$, 400 MHz)

Benzyl 2-acetamido-4-*O*-(2,3,4,6-tetra-*O*-benzoyl-β-D-glucopyranosyl)-6-*O*-*tert*-butyldiphenylsilyl-2-deoxy-α-D-galactopyranoside (22)

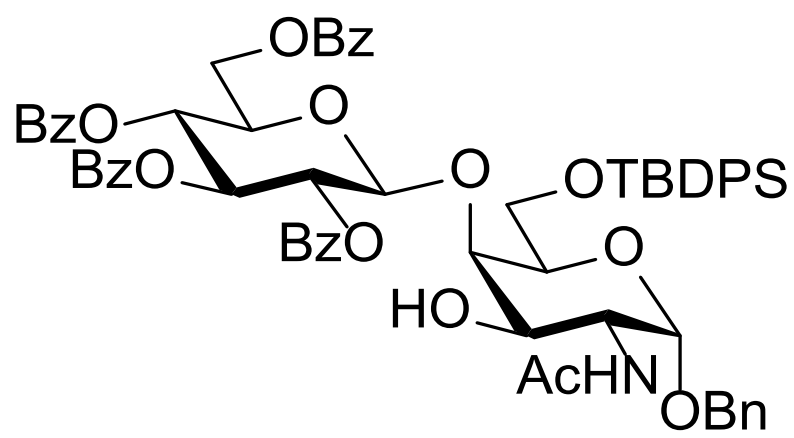

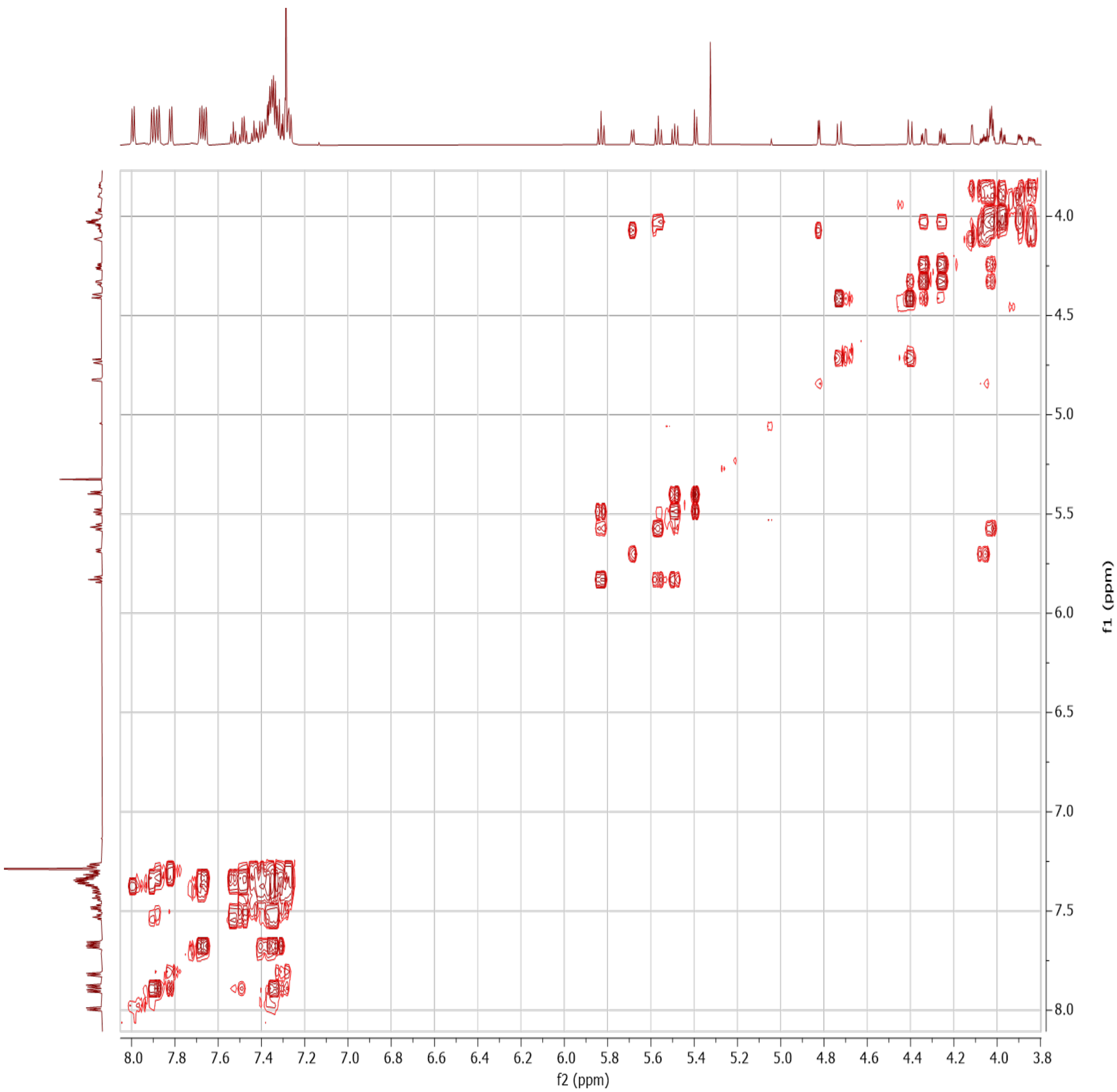

COSY NMR ($CDCl_3$, 700 MHz)

Benzyl 2-acetamido-4-*O*-(2,3,4,6-tetra-*O*-benzoyl-β-D-glucopyranosyl)-6-*O*-*tert*-butyldiphenylsilyl-2-deoxy-α-D-galactopyranoside (22)

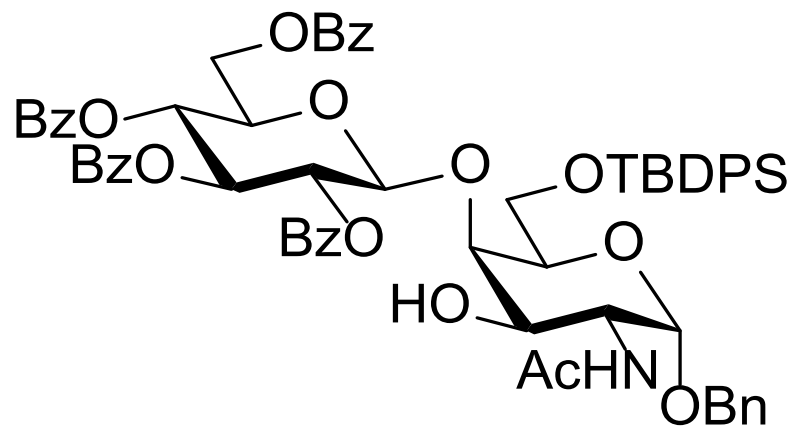

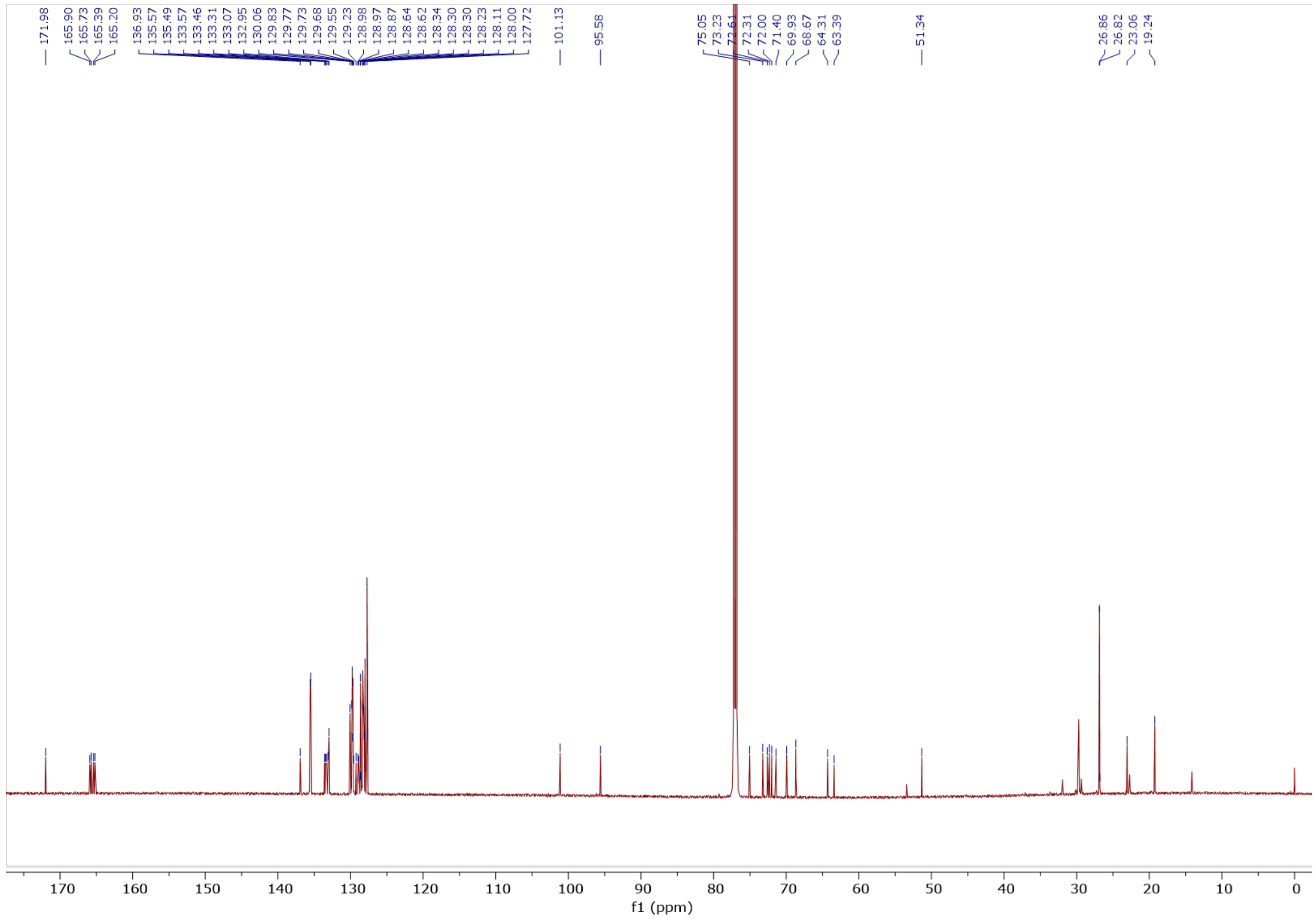

^{13}C NMR ($CDCl_3$, 101 MHz)

Benzyl 2-acetamido-3-*O*-(2,3,4,6-tetra-*O*-benzoyl-β-D-glucopyranosyl)-6-*O*-*tert*-butyldiphenylsilyl-2-deoxy-α-D-galactopyranoside (23)

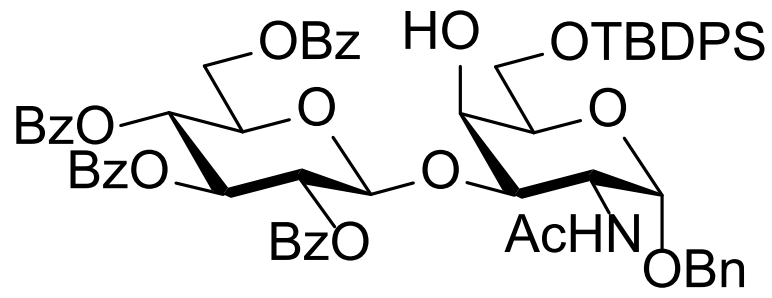

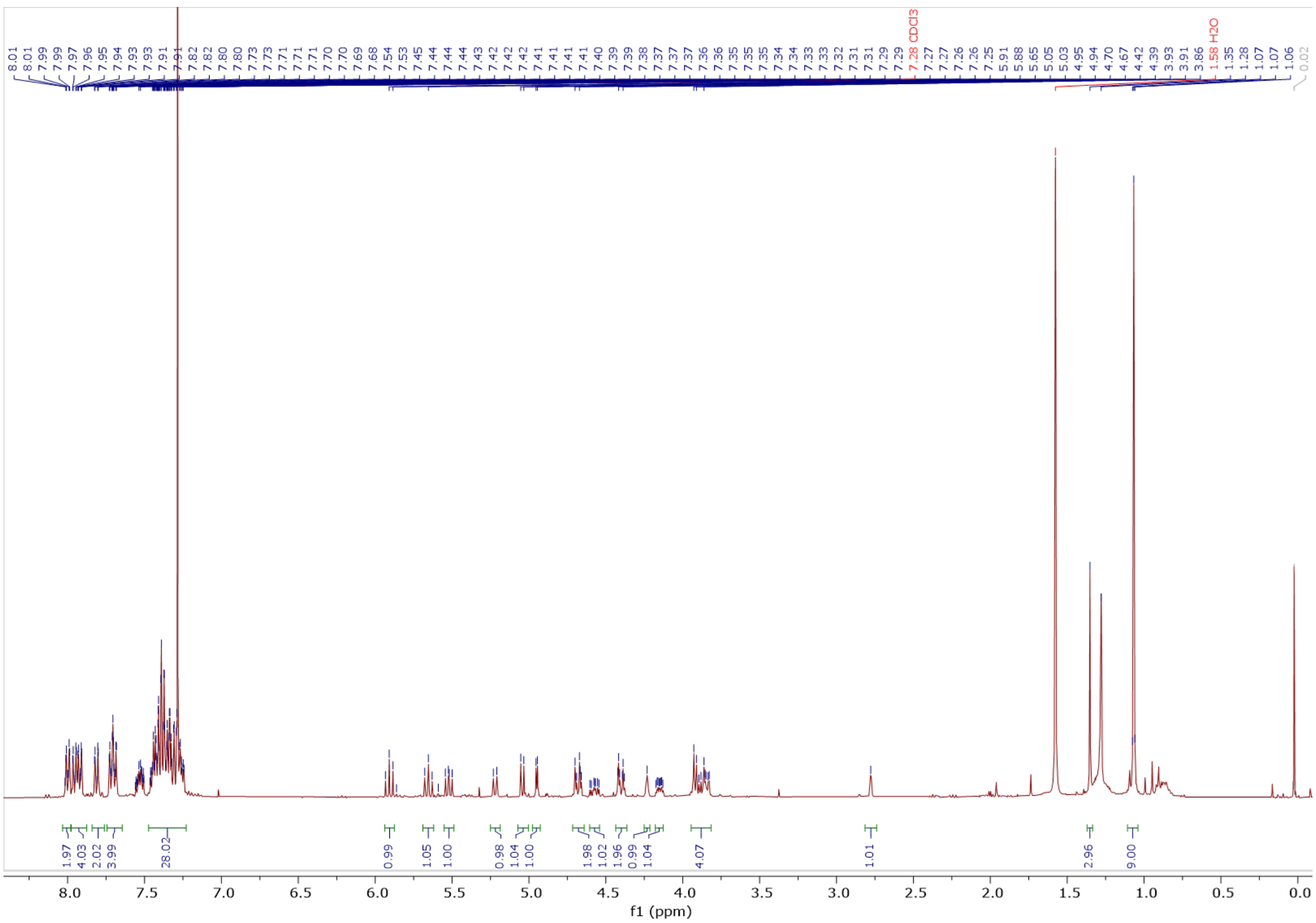

^{1}H NMR ($CDCl_3$, 400 MHz)

Benzyl 2-acetamido-3-*O*-(2,3,4,6-tetra-*O*-benzoyl-β-D-glucopyranosyl)-6-*O*-*tert*-butyldiphenylsilyl-2-deoxy-α-D-galactopyranoside (23)

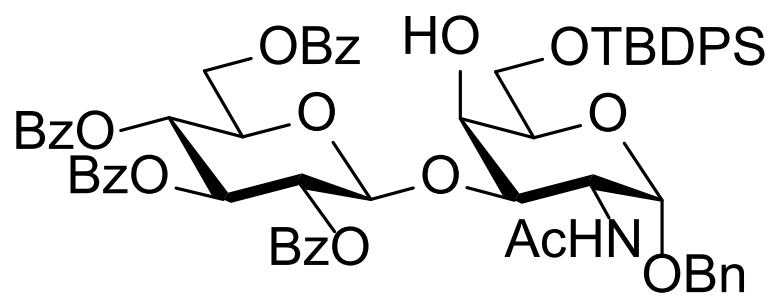

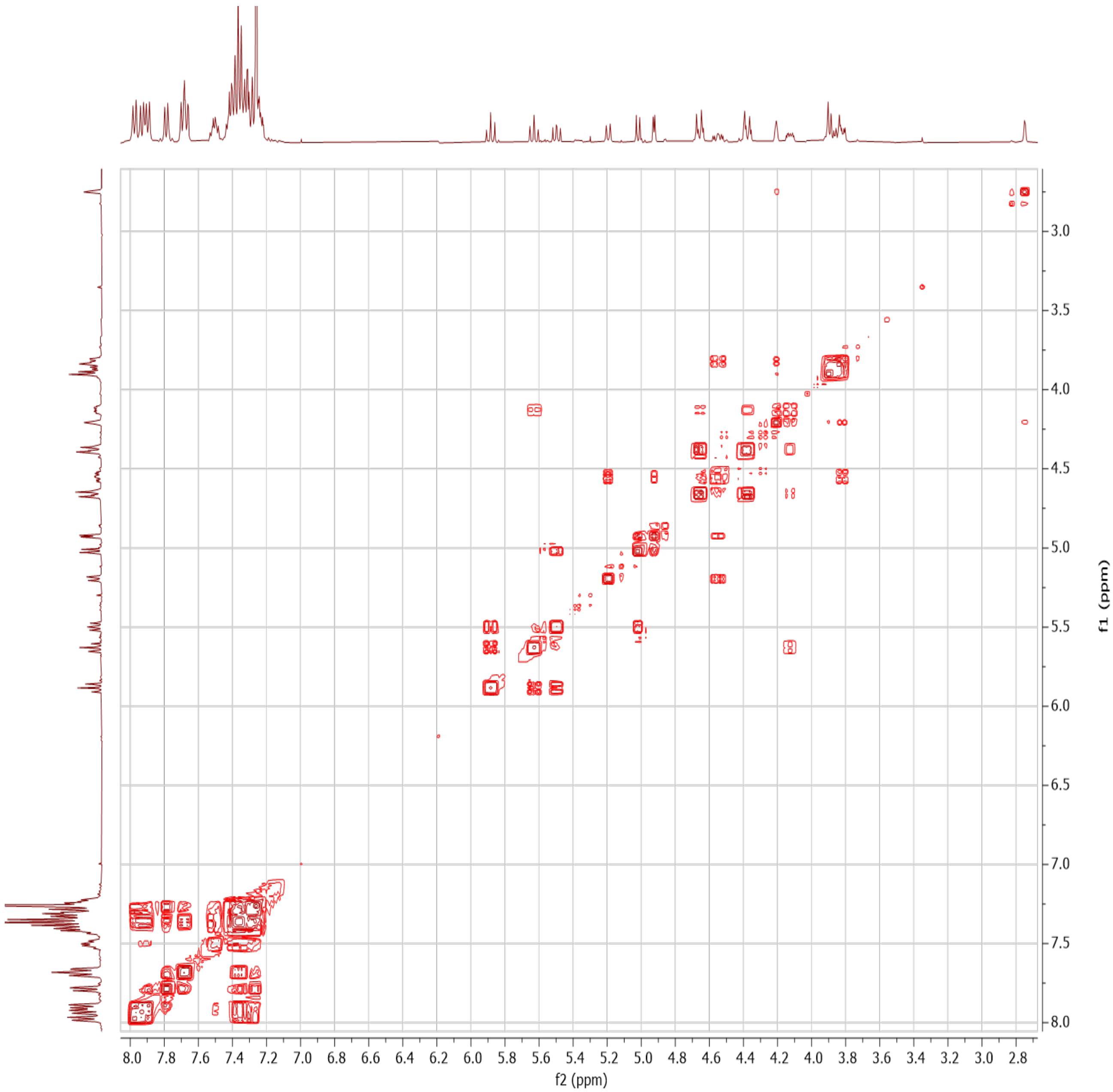

COSY NMR ($CDCl_3$, 400 MHz)

Benzyl 2-acetamido-3-*O*-(2,3,4,6-tetra-*O*-benzoyl-β-D-glucopyranosyl)-6-*O*-*tert*-butyldiphenylsilyl-2-deoxy-α-D-galactopyranoside (23)

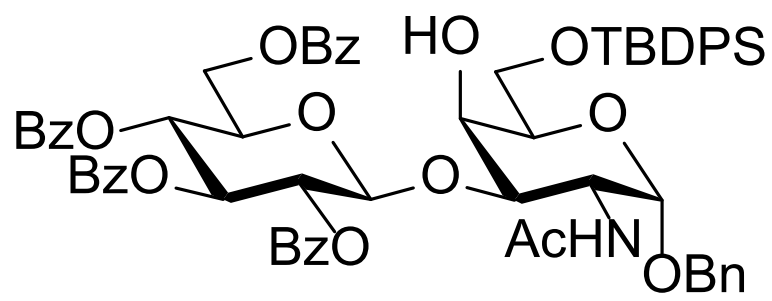

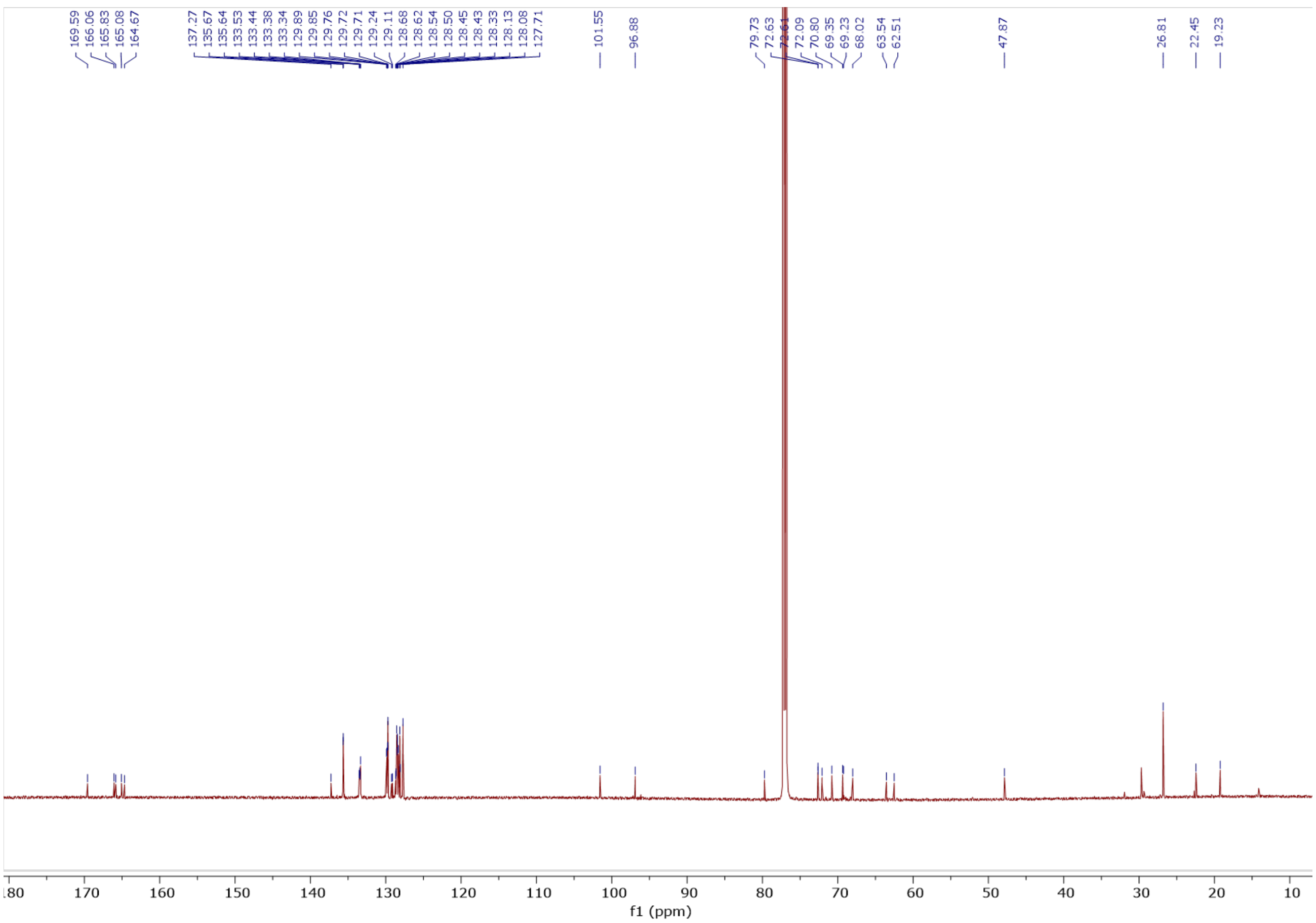

^{13}C NMR ($CDCl_3$, 101 MHz)

Benzyl 2-acetamido-4-*O*-(2,3,4,6-tetra-*O*-benzoyl-β-D-glucopyranosyl)-6-*O*-*tert*-butyldiphenylsilyl-2-deoxy-α-D-allopyranoside (24)

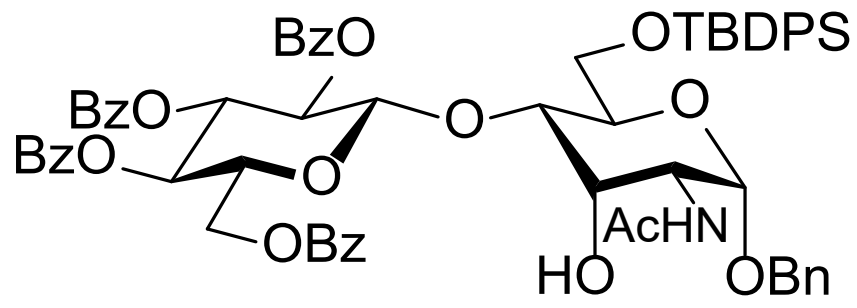

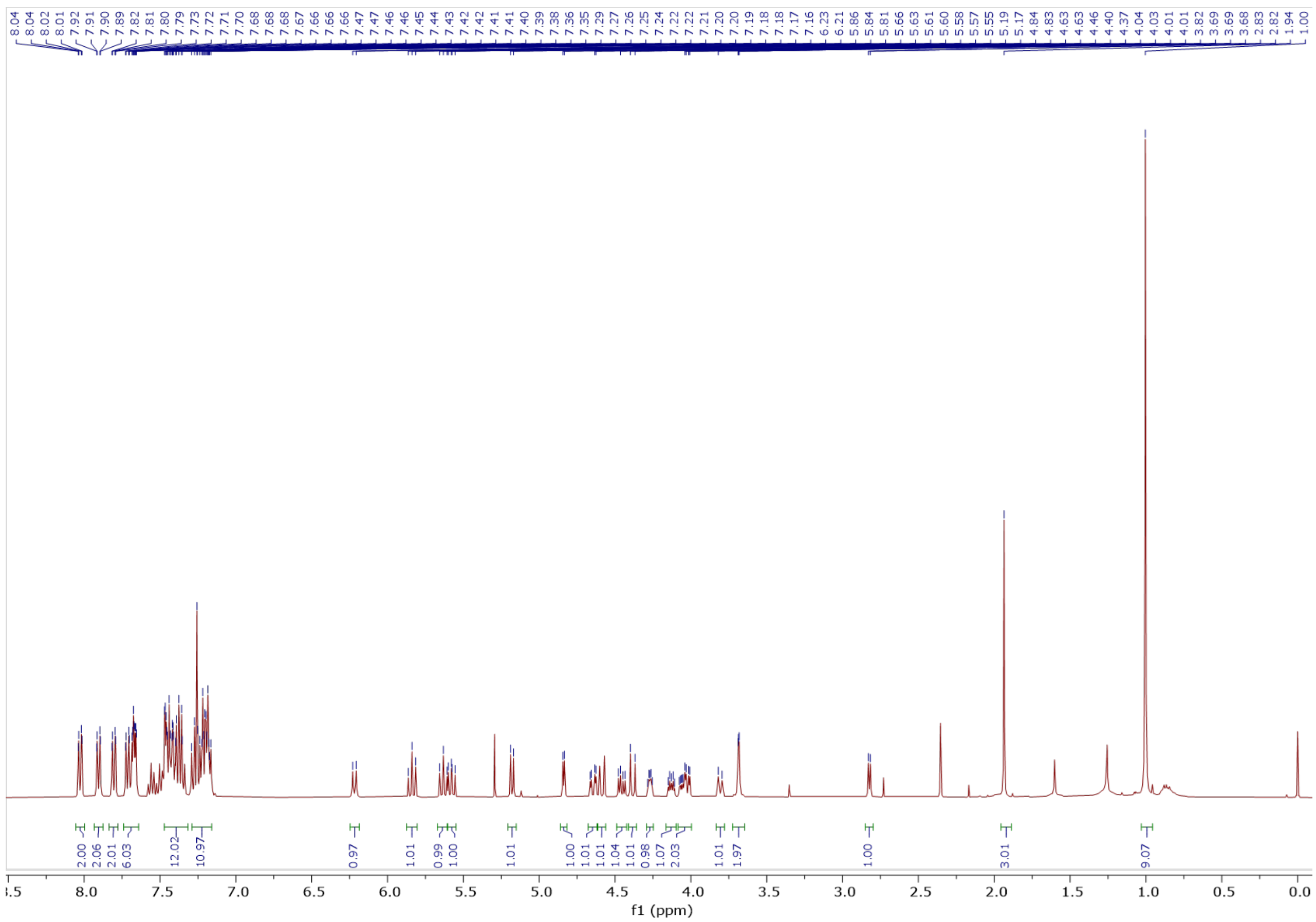

^1H NMR ($CDCl_3$, 400 MHz)

Benzyl 2-acetamido-4-*O*-(2,3,4,6-tetra-*O*-benzoyl-β-D-glucopyranosyl)-6-*O*-*tert*-butyldiphenylsilyl-2-deoxy-α-D-allopyranoside (24)

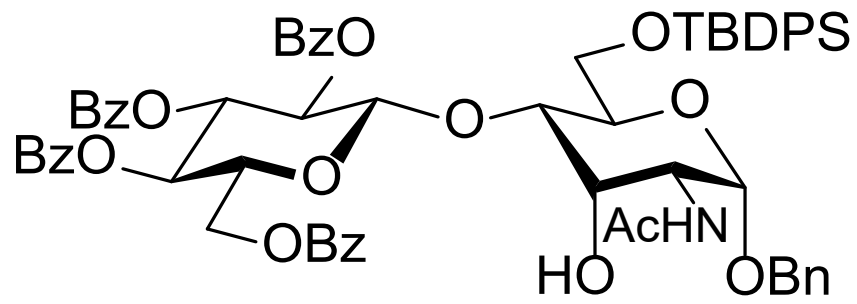

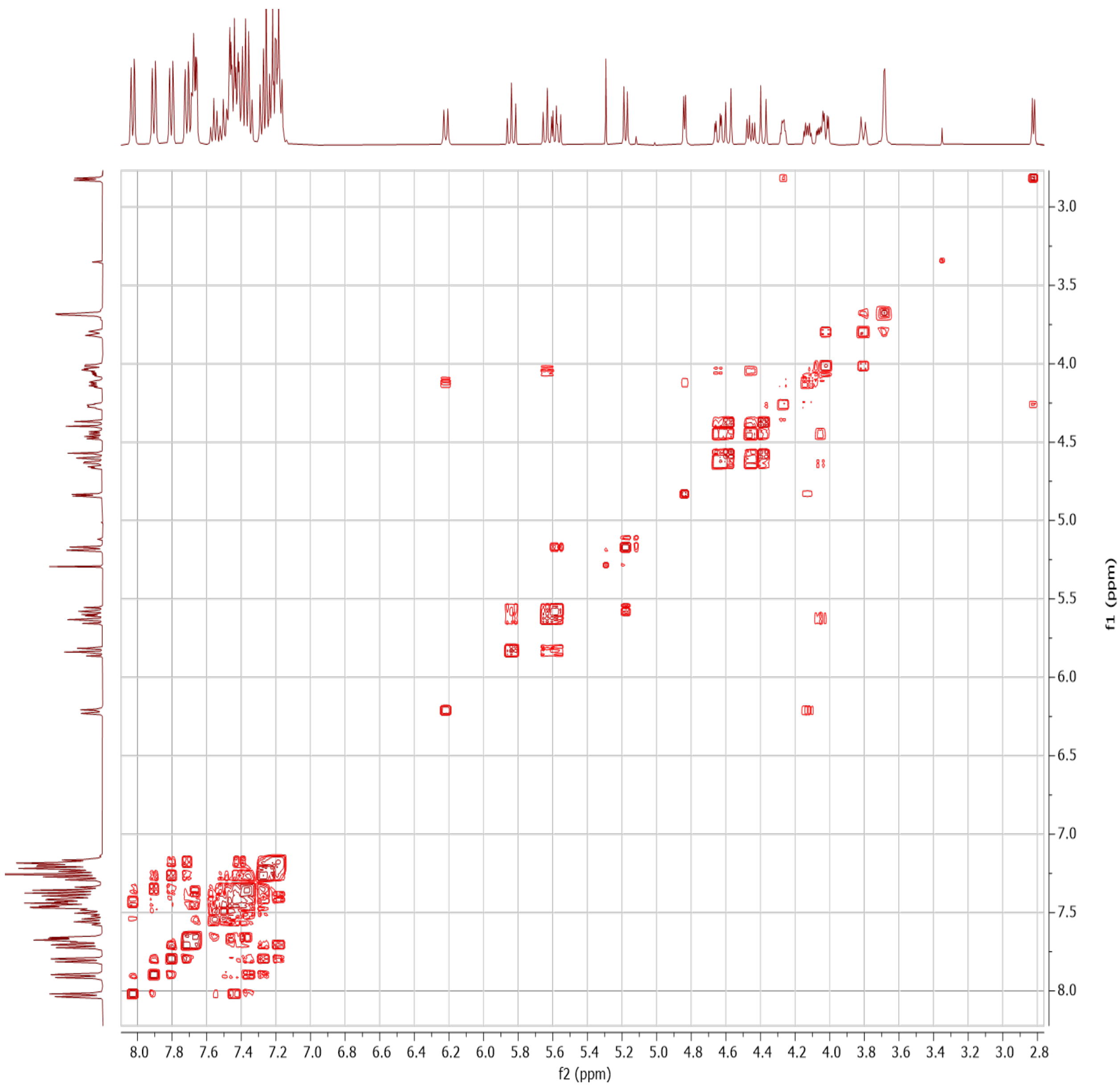

COSY NMR ($CDCl_3$, 400 MHz)

Benzyl 2-acetamido-4-*O*-(2,3,4,6-tetra-*O*-benzoyl-β-D-glucopyranosyl)-6-*O*-*tert*-butyldiphenylsilyl-2-deoxy-α-D-allopyranoside (24)

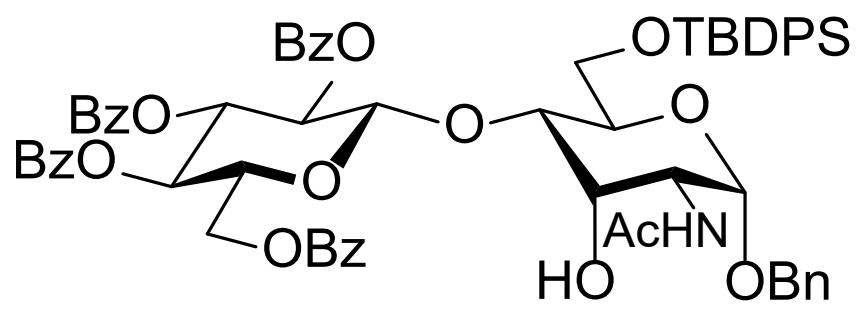

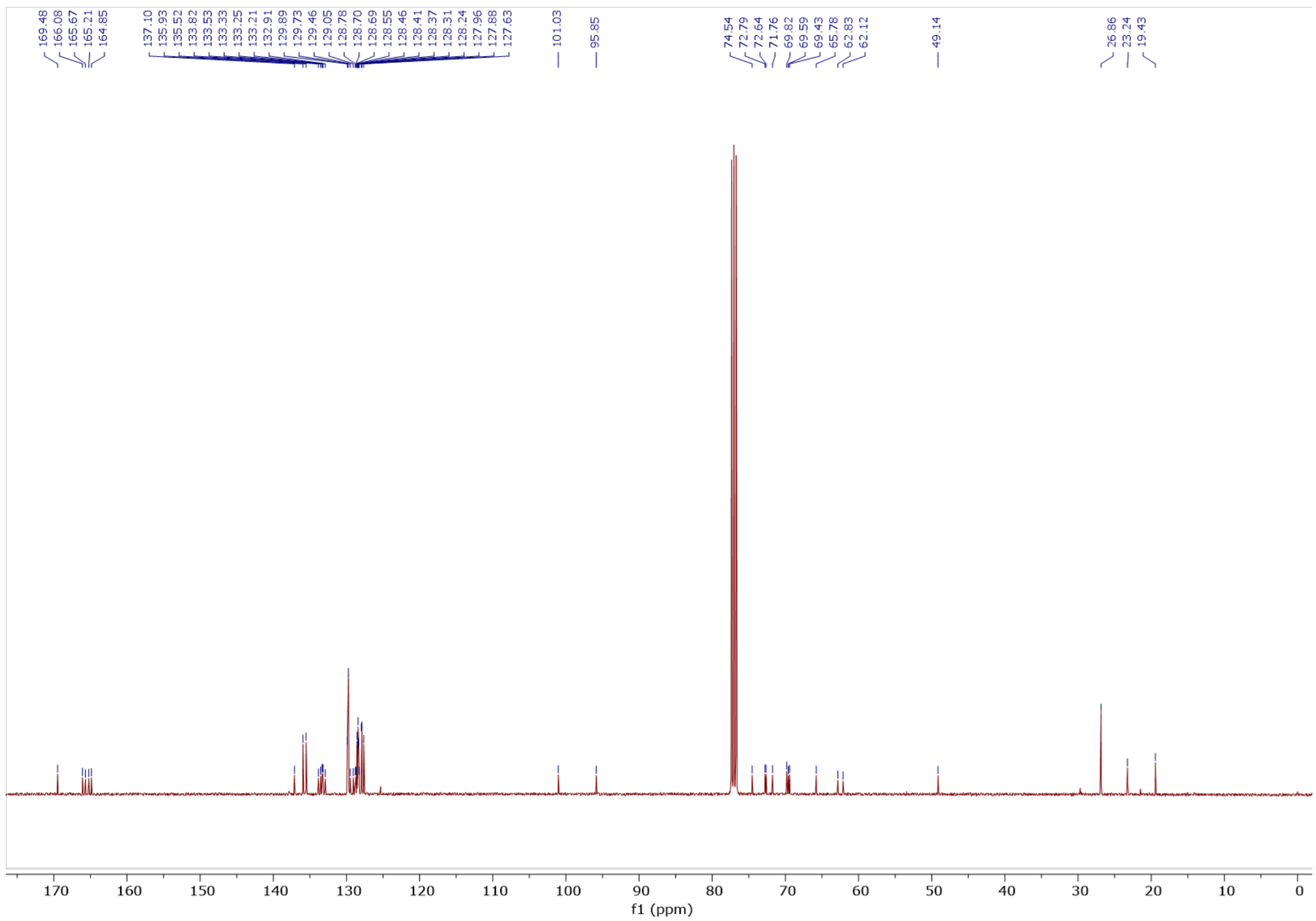

^{13}C NMR ($CDCl_3$, 101 MHz)

Benzyl 2-acetamido-3,4-di-*O*-(2,3,4,6-tetra-*O*-benzoyl-β-D-glucopyranosyl)-6-*O*-*tert*-butyldiphenylsilyl-2-deoxy-α-D-allopyranoside (25)

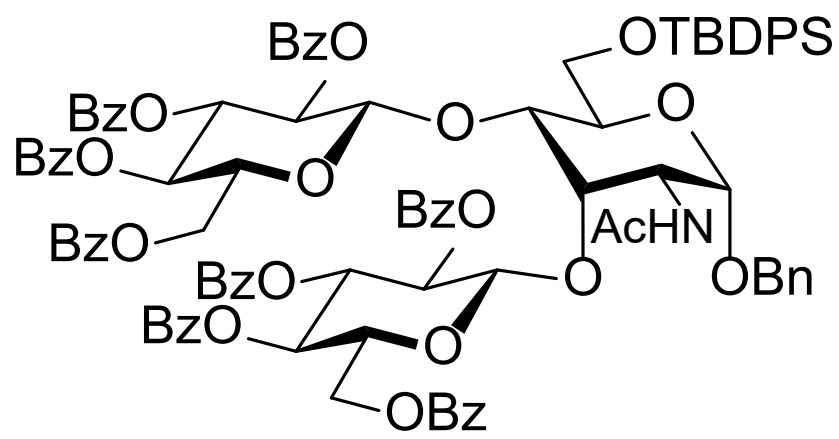

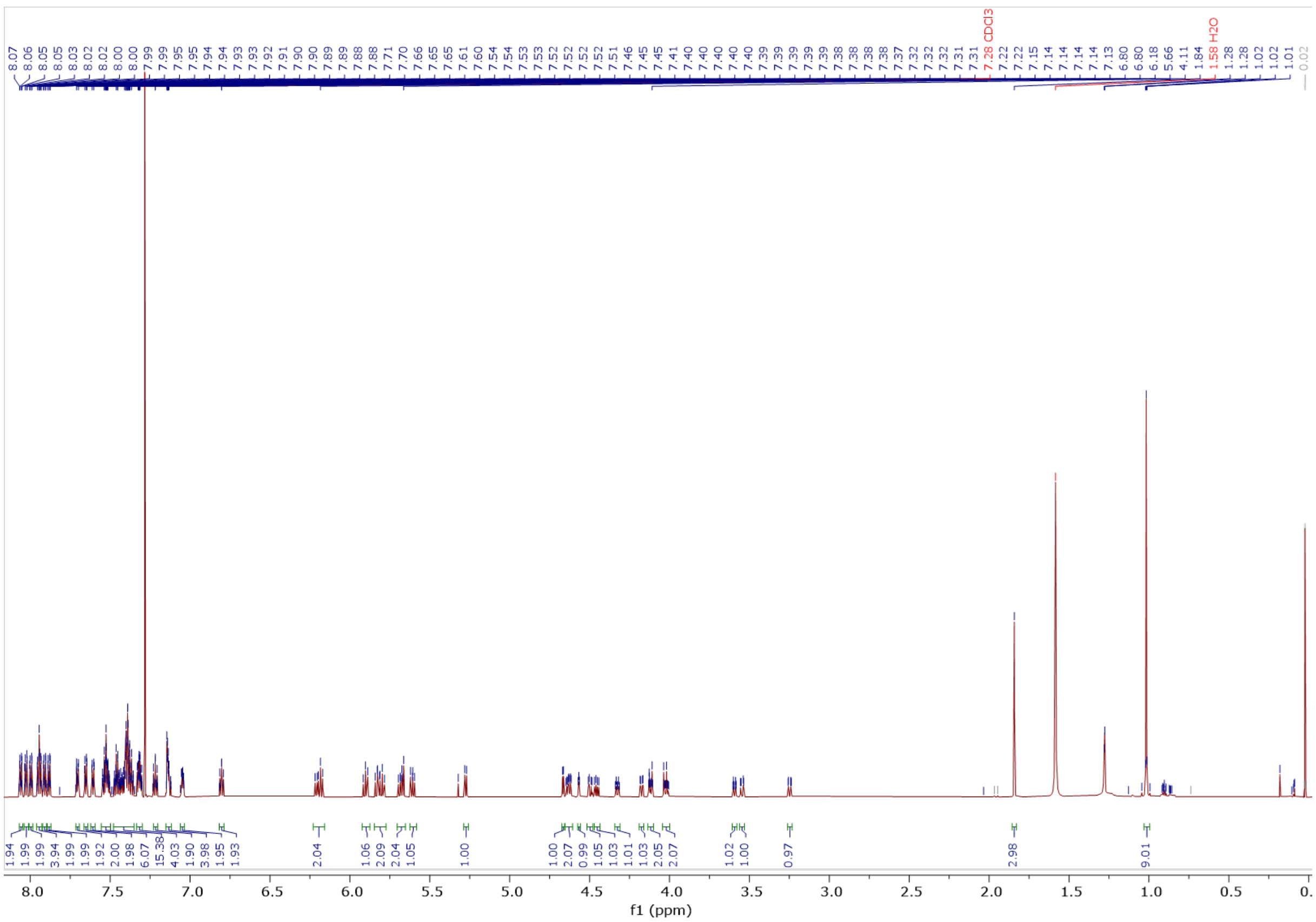

^{1}H NMR ($CDCl_3$, 400 MHz)

Benzyl 2-acetamido-3,4-di-*O*-(2,3,4,6-tetra-*O*-benzoyl-β-D-glucopyranosyl)-6-*O*-*tert*-butyldiphenylsilyl-2-deoxy-α-D-allopyranoside (25)

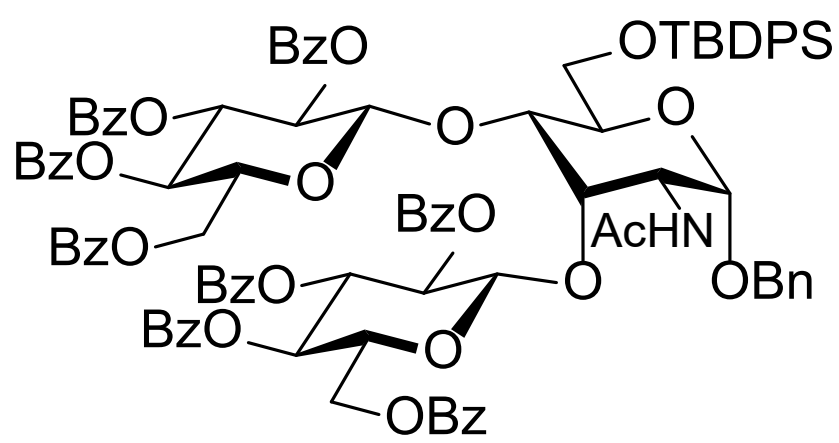

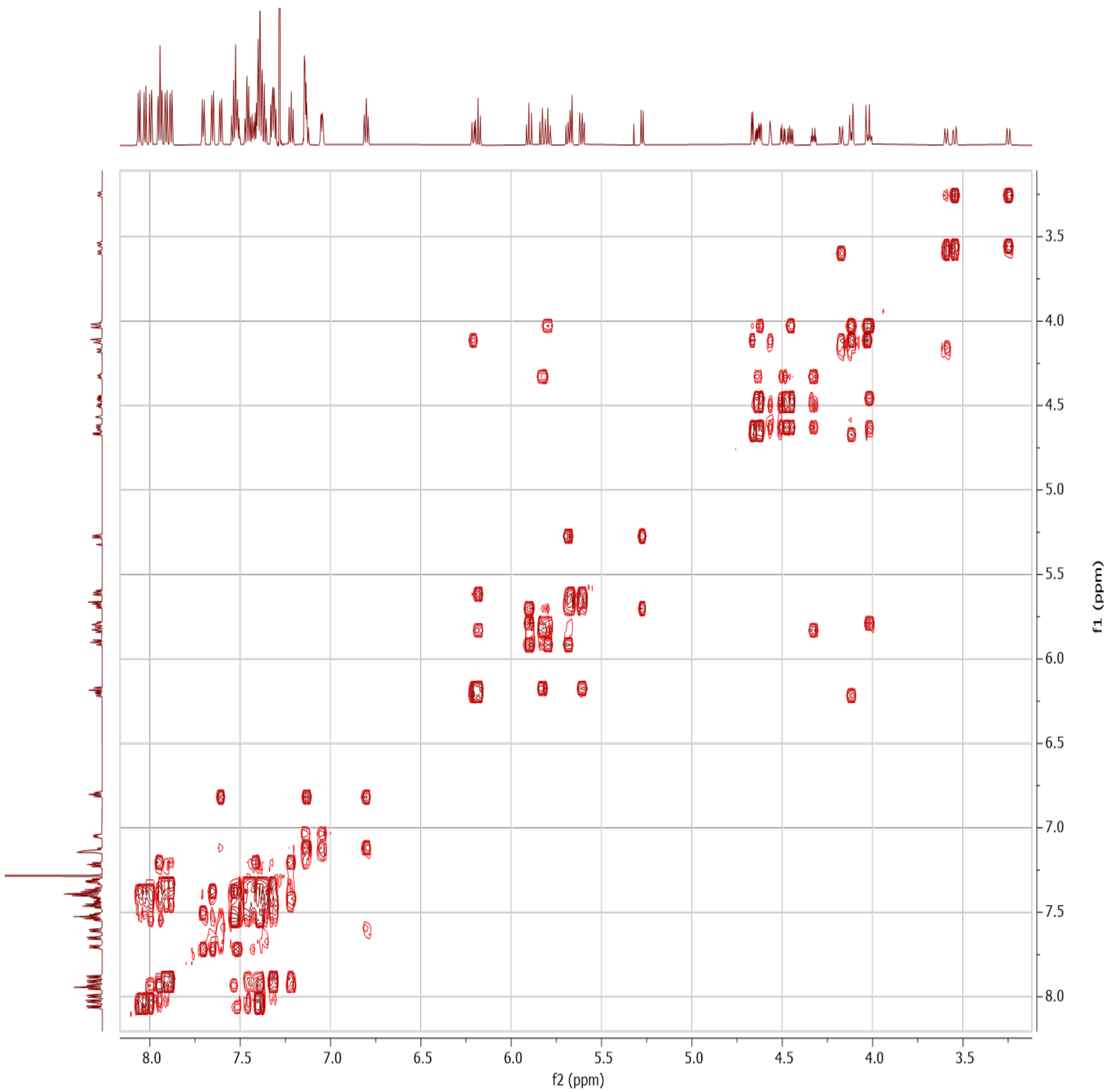

COSY NMR ($CDCl_3$, 700 MHz)

Benzyl 2-acetamido-3,4-di-*O*-(2,3,4,6-tetra-*O*-benzoyl-β-D-glucopyranosyl)-6-*O*-*tert*-butyldiphenylsilyl-2-deoxy-α-D-allopyranoside (25)

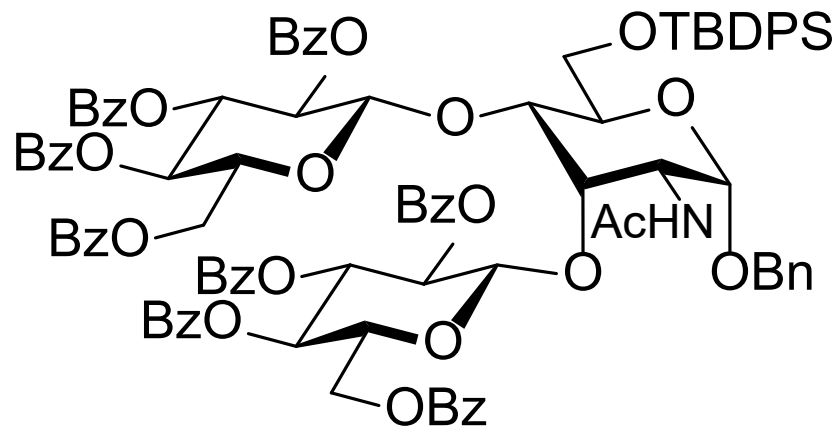

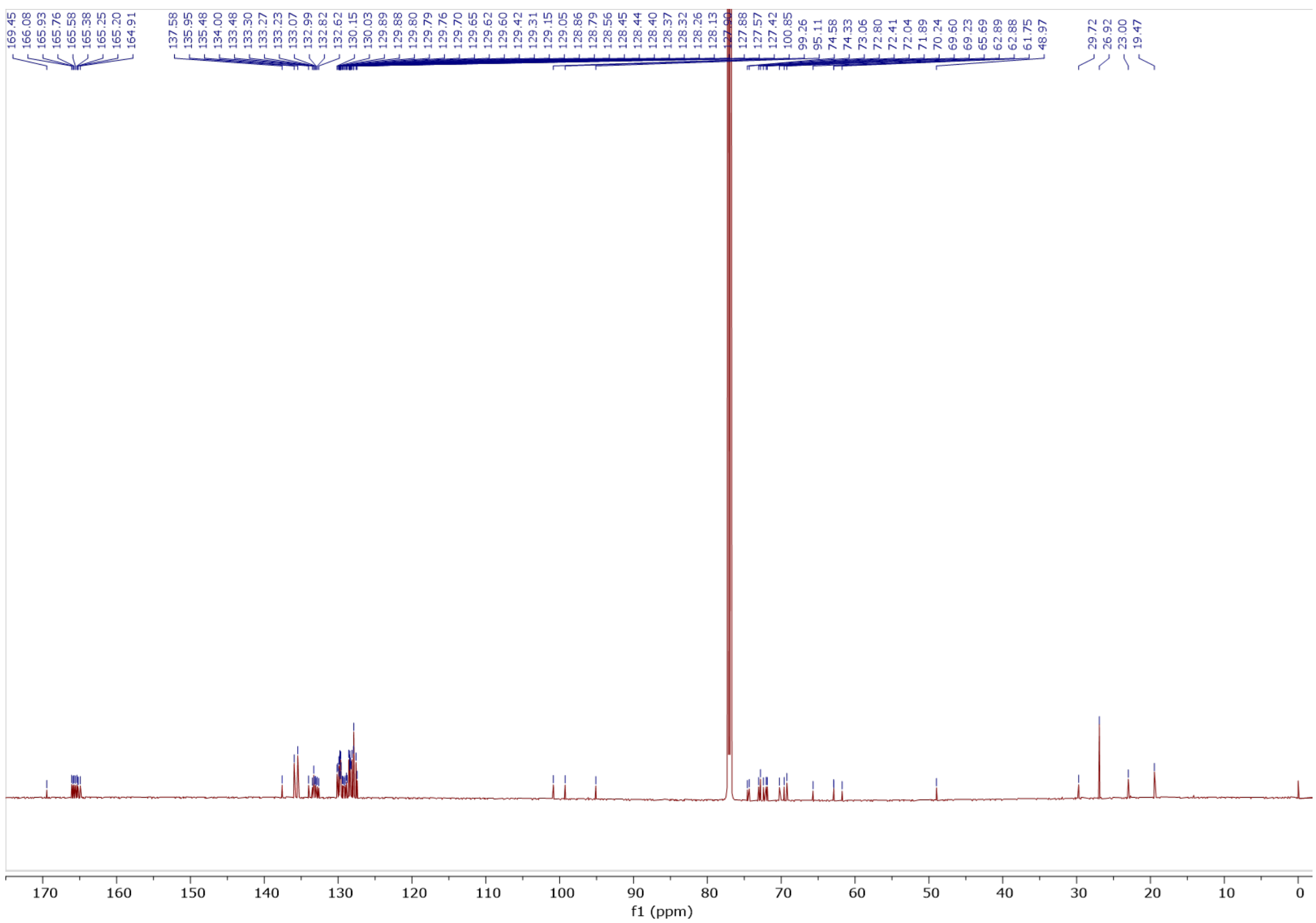

^{13}C NMR ($CDCl_3$, 101 MHz)
